# Amyloid Cross-Interactions through the Lens of Simulations: The Case of Aβ–IAPP

**DOI:** 10.1021/acs.jpcb.5c04536

**Published:** 2025-10-06

**Authors:** Xenophon Xenophontos, Anastasia Vlachou, Ryleigh K. Hunt, Phanourios Tamamis

**Affiliations:** † Artie McFerrin Department of Chemical Engineering, College of Engineering, 14736 Texas A&M University, College Station, Texas 77843, United States; ‡ Department of Materials Science and Engineering, College of Engineering, Texas A&M University, College Station, Texas 77843, United States

## Abstract

While significant progress has been made in developing approaches to study amyloid self-assembly leading to homomeric fibril formation by identical proteins, our understanding of heteromeric cross-interactions formed by different proteins is limited. Understanding such cross-interactions, resulting from cross-seeding and/or coaggregation, is undeniably key due to their occurrence in biology and their implication in diseases. We have developed a new computational approach for the study of heteromeric amyloid cross-interactions in axial stacking, biased molecular dynamics (MD) simulations, followed by conventional MD simulations. The biased MD can mimic, facilitate, and accelerate the cross-interaction process, allowing the cross-interacting entities to adapt their conformations and interactions with each other. Our approach has been applied to delineate the amyloid cross-interactions that can be formed by Aβ and IAPP in axial stacking. The computationally derived conformers demonstrate a high degree of compatibility in β-sheet interactions and side chain contacts in the Aβ–IAPP cross-interaction and beyond, forming an amyloid steric zipper nearly throughout the structure. Our results depict that IAPP in the junction can cross-interact intermolecularly with an Aβ fibril nearly as favorably as with an IAPP fibril. At least for the polymorphs examined, while both Aβ and IAPP can adapt to each other in the junction, IAPP has a higher propensity to adopt a polymorph that is formed by homomeric Aβ. This is in line with the notion that an Aβ fibril can be a seed for IAPP. We suggest that the capacity of amyloids to adopt different polymorphs and the types of polymorphs they can adopt determine and drive their cross-interacting capacities; this is valid at least for the cross-interaction investigated here and could hold for other amyloid cross-interactions in general.

## Graphical abstract

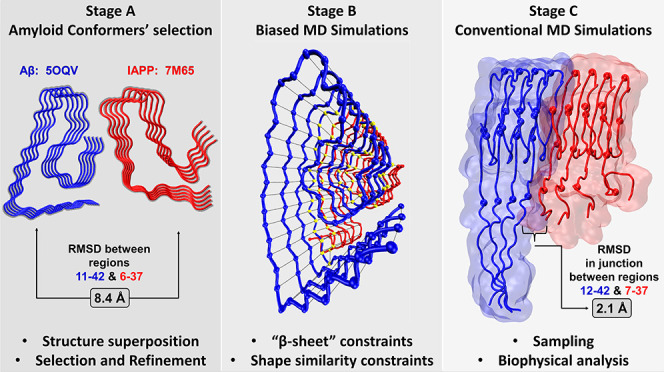

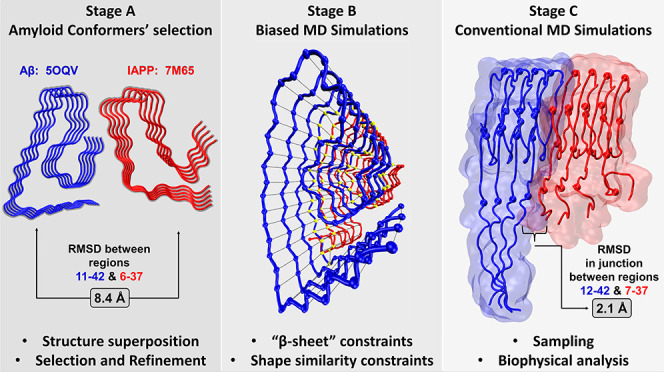

## 1. Introduction

Over the past decade, the field of amyloid structural biology has advanced significantly.(1) Among others, amyloid fibril structures have been resolved for amyloid-β (Aβ),(1) tau,(1) α-synuclein,(1) amylin (IAPP),(1) and TDP-43,(2) which are linked to disorders including Alzheimer’s disease (AD), Parkinson’s disease (PD), type II Diabetes (T2D), and Amyotrophic Lateral Sclerosis (ALS),(2) respectively. Furthermore, amyloid fibril structures have been resolved for functional amyloids, which are involved in biological processes, including hormone storage. Notably, the growing number of amyloid fibril structures resolved has shed light into the significance of amyloid fibril polymorphism, the capacity of a single protein to form structurally diverse fibril conformations. Polymorphic variants, among other implications, have been associated with distinct disease phenotypes, highlighting the need to examine fibril structures under varying environmental conditions and in relation to disease mechanisms.

Hallmarks of most amyloid diseases are multicomponent assemblies, such as plaques, tangles, Lewy bodies, and inclusions.(13) Aβ-linked plaques, huntingtin-linked neuronal intracellular inclusions, tau-linked neurofibrillary tangles, and α-synuclein-linked Lewy bodies are multicomponent systems comprising various other proteins. Experimental studies revealed that when coaggregation and cross-seeding occur during the amyloid formation of multiple proteins and peptides, it results in multicomponent amyloid assemblies.(13) Additionally, the simultaneous presence of different amyloid-associated pathologies within a single individual has been reported, suggesting potential cross-talk between distinct amyloidogenic protein species during their amyloid growth. Despite its significance, cross-amyloid aggregation remains insufficiently investigated, and there is little information regarding the sequence/structure-dependent aggregation mechanisms governing such processes.(24)

Among the most extensively investigated amyloid cross-interactions are Aβ–IAPP, Aβ–tau, Aβ–α-synuclein, and α-synuclein–tau.(24) The amino acid sequence of proteins has fundamentally been regarded as a key factor in determining their propensity to coaggregate or initiate amyloid cross-seeding. Studies on the aggregation of immunoglobulin domains have demonstrated that coaggregating partners exhibiting high sequence similarity (exceeding ∼70%) tend to function more effectively as cross-seeding or copolymerizing pairs than those with lower (30–40%) or no sequence similarity. Nevertheless, numerous experiments have revealed that proteins with minimal or no sequence similarity are also capable of undergoing rapid coaggregation and can initiate amyloid cross-seeding more efficiently than self-seeding in some cases. The complexity of this phenomenon increases further when considering the diversity of cross-interaction mechanisms,(26) as well as the influence of metabolites(13) in such interactions. Metabolite amyloid can additionally induce other soluble proteins to aggregate, which can lead to a higher epidemiological risk of neurodegeneration or cancer in patients inflicted with inborn error metabolism. Moreover, cross-interactions may include cross-seeding,(29) coaggregation(29) or even cross-inhibition, while amyloid structures composed of biologically relevant individual metabolites have been shown to aggressively promote cross-seeding in other protein or peptide species.(13) Furthermore, amyloid cross-interactions could involve several models of association between two different fibrils, including elongation (axial) or lateral stacking.(26)

Notably, Aβ and IAPP coexist in both the human brain and pancreas, and there is a clinical overlap between AD and T2D. Aβ and IAPP have been indicated to form heteromeric interactions with each other, and to possess the capacity to form mixed or heteromeric aggregates. Five short peptide segments of Aβ and IAPP, Aβ(27–32), Aβ(35–40), Aβ(19–22), IAPP(8–18), and IAPP(22–28), were identified as regions of the Aβ–IAPP cross-interaction interface; these peptides are able to self- and to cross-interact and are high-affinity ligands of both Aβ and IAPP.(44) Additionally, peptides were designed to mimic the Aβ core and were demonstrated to act as cross-amyloid inhibitors of both IAPP and Aβ.(45) Extensive research has been conducted to examine the interplay between Aβ and IAPP, particularly in the context of coaggregation and cross-seeding phenomena. Aβ and IAPP interact to form amorphous heterocomplexes with enhanced toxicity in neuronal cells.(38) Moreover, it was shown that IAPP in the presence of Aβ forms inhomogeneous oligomeric aggregates.(47) Additionally, in vitro experiments showed that Aβ–IAPP heteroassemblies adsorbed, aggregated, and permeabilized β-cell membranes system significantly slower than IAPP alone, but much faster than Aβ alone.(48) Fibrils composed of Aβ and IAPP are capable of functioning as seeds, each promoting the other’s amyloid aggregation; however, this mutual cross-seeding is less efficient than their respective self-seeding processes. IAPP fibrils were shown to be poor seeds for Aβ elongation, and Aβ fibrils were shown to be effective seeds for IAPP elongation in vitro(20) and in vivo.(21) Moreno-Gonzalez et al., observed that introducing IAPP seeds accelerated Aβ aggregation in vitro, resulting in fibrils that contained both peptides.(23) Nevertheless, studies have mainly relied on kinetic data as a means to interpolate the likelihood for heteromeric fibrils; thereby, the direct observation of Aβ–IAPP mixed aggregates still eludes.(36) Notably, we still lack in detail structural information on these aggregates.(36) Particularly, a recent study showed that coaggregation of the peptides from the monomeric stage leads to the formation of unique polymorphs, in which both peptides undergo structural deviation from their native states, whereas seeding with preformed IAPP fibrils leads to aggregates similar to native Aβ.(36)

Computational studies have provided additional important insights into molecular interactions driving this cross-talk. A portion of these studies have investigated cross-interactions between fragments or full-length Aβ and IAPP peptides, providing noteworthy results. The continuously increasing number of resolved amyloid fibril structures formed by fragments or full-length Aβ and IAPP has provided new avenues to the problem, allowing the investigation of interactions between fibrils of one type and monomers of the other type, as well as interactions between the two fibrils. Zheng’s group initially found that lateral association, as well as axial elongation, comprise the most probable cross-seeding aggregation pathways when compared with other pathways and provided insightful maps of interactions between amino acids in cross-interacting peptides.(26) An additional study by Zheng’s group implied that Aβ–IAPP assembly might cause damage to the cell by inducing changes to calcium homeostasis and cell membrane phase;(52) hence, it provided us with a better fundamental understanding about a potential pathological association between AD and T2D and of cross-seeding interactions between Aβ and IAPP on cell membranes.(52) Miller’s group depicted that Aβ oligomers show a preference to interact with IAPP oligomers via in-register interactions to form single-layer conformations, rather than double-layer conformations.(50) Also, they observed that IAPP oligomers showed a preference to interact with Aβ oligomers to form single-layer polymorphic conformations.(50) Additionally, in some cases of double-layer conformations of Aβ–IAPP oligomers, cross-seeding, the IAPP oligomers destabilized Aβ, therefore inhibiting Aβ aggregation.(50) Fan et al. suggested that Aβ–IAPP coaggregation does not affect their capacity to recruit additional monomers, thus promoting the growth of larger β-sheet-rich aggregates, while simulations of cross-seeding between Aβ and IAPP showed that both of their fibrils could act as mutual templates, helping each other’s monomers to convert into β-sheets at the fibril elongation ends.(51) Computational studies by Song et al. showed that Aβ monomers had a preference to bind to the elongation ends of IAPP preformed fibrils;(55) according to the authors, because of sequence mismatch, the Aβ monomers could not form multiple stable β-sheets with the exposed ends of IAPP fibrils and directly grow onto them.(55)

Despite the noteworthy results obtained in previous experimental and computational studies, we still lack highly ordered cross-interacting models of full-length Aβ and IAPP, shedding light on how the sequence and shape similarity could potentially contribute to a highly symmetric heteromeric ordered arrangement between the two. This gap of knowledge could be attributed to several factors, including the limited work on the development of approaches to delineate the structures of amyloid cross-interactions within their ordered heteroassemblies. Notably, to the best of our knowledge, there are no experimentally resolved structures of amyloid cross-interactions in axial stacking. Also, despite the noteworthy computational method PACT,(59) capable of predicting the ability of proteins to cross-interact, the models produced by PACT(59) are built using the same template. As a result, this does not take into consideration the fact that different amyloids form different conformers, and even the same amyloid can adopt different polymorphs. The axial cross-interaction problem is even more challenging to solve, as cross-interacting amyloids are expected to “dynamically” interact, influencing each other’s conformations, and adapt to each other through local adjustments within the interaction interface, which is referred to by us as the junction. For example, in amyloid cross-seeding involving axial stacking, it is expected that the two heteromeric structures will preserve their integrity away from the junction, yet while approaching the junction, the heteromeric amyloids are expected to conformationally adapt and optimally interact with each other.

In this study, we present a new computational approach to investigate heteromeric amyloid cross-interactions of axial stacking and demonstrate its application to derive the first, to our knowledge, ordered heteromeric full-length cross-interacting assemblies of Aβ and IAPP. The study of fibril nucleation, axial and/or lateral stacking between Aβ and IAPP has been investigated in several studies, including. The goal of our study was to investigate how the two entities could optimally form axial stacking interactions and thus provide insights into the Aβ and IAPP cross-interaction in general. In this context, our goal was not to compare different forms of stacking but to elucidate how axial stacking interactions between the two can be formed and to what extent these interactions can lead to optimally formed β-sheets in the junction and beyond. To address this, we have developed an approach that considers the ability of two cross-interacting entities to refine their individual assembly and adjust in the presence of the other, resulting in an ordered configuration formed by the two. This is achieved using biased molecular dynamics (MD) simulations, with constraints enhancing the cross-interaction between the two entities. Constraints are used to mimic, facilitate, and accelerate the potential naturally occurring cross-interaction process, allowing the cross-interacting entities to adapt their conformations and interactions with each other. We consider that this approach can enable the study of a series of cross-interacting systems and further expand our overall knowledge on amyloid cross-interactions.

## 2. Methodology

**Figure 1:**
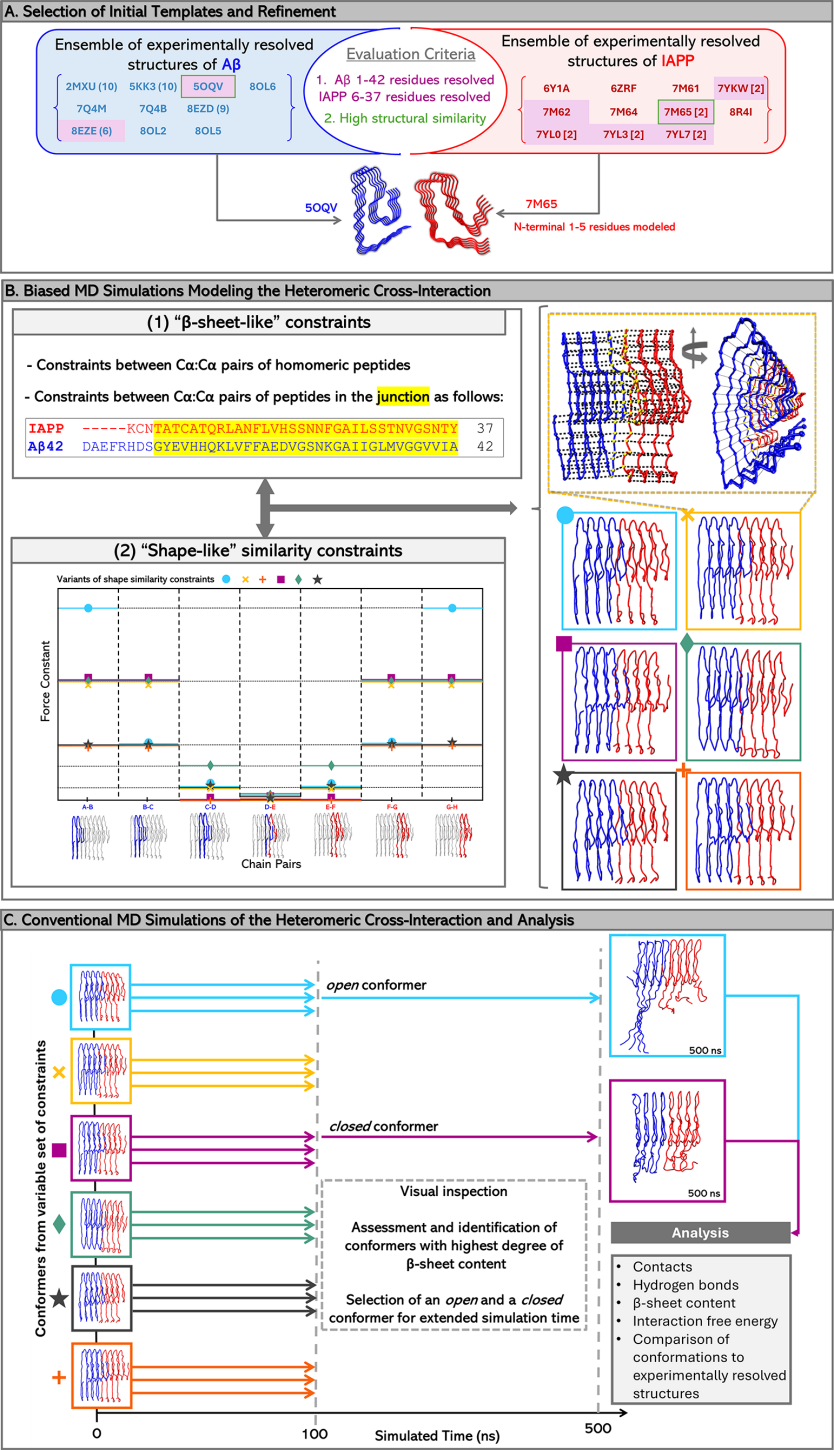
Diagram shows a schematic overview of the computational framework used to investigate the heteromeric fibrils of Aβ and IAPP, decomposed into three stages: Panel A depicts the selection of the initial homomeric templates used for the modeling of the heteromeric conformer. Conformers sufficing the first criterion (i.e., 1–42 residues resolved in Aβ and at least 6–37 residues resolved in IAPP) are highlighted in purple. Subsequently, upon RMSD calculations for each pair of models, the conformers with the highest structural similarity were selected and are enclosed in green boxes. Structures with multiple conformers, along with the number of conformers, are shown in parentheses, while structures with multiple fibrils resolved are shown in brackets. Panel B depicts the short MD simulations under constraints (referred to as biased), which were employed to facilitate and accelerate the formation of the junction between the two cross-interacting conformers. A sequence alignment, with a high gap penalty, was used to determine the most efficient alignment in order for the similar regions of Aβ and IAPP to be aligned (highlighted in yellow). Based on this alignment, β-sheet-like constraints were applied between Cα:Cα atom pairs of the aligned residues. The same type of constraints were also applied between Cα:Cα atom pairs of corresponding residues belonging to neighboring homomeric chains, as shown in the structures outlined with a yellow dashed line. The Cα atoms in the interface of Aβ and IAPP that were forced to interact with constraints are shown in yellow. In addition to β-sheet-like constraints, shape-like similarity constraints were imposed during the biased MD simulations. Six different variations of the shape-like similarity constraints were tested independently, resulting in six different heteromeric conformers. The shape-like similarity constraints variants are shown qualitatively in the plot of Panel B, while the produced heteromeric conformers are shown in blue (circle), yellow (“X”), purple (rectangle), green (diamond), black (star), and orange (cross). The resulting structures served as initial conformers for the subsequent unconstrained MD simulations (referred to as conventional), as shown in Panel C. Each was simulated in triplicate runs for 100 ns to sample heteromeric conformations. Using the degree of β-sheet content of each run as an evaluation metric, two conformers, referred to by us as *open* and *closed*, were extended to 500 ns, and their trajectories were used to conduct structural and energetic analysis. The backbone of the initial template structures and the cross-interaction models is shown in tube representation; blue for Aβ and red for IAPP. The Aβ (5OQV(64)) and IAPP (7M65(65)) structures and the cross-interaction models were visualized and captured using VMD.(68)

Figure 1 summarizes the framework of our methodology to study the amyloid cross-interaction between Aβ and IAPP. The framework can be decomposed into three major stages: (Stage A) The selection of the initial templates and a refinement (if needed) of these templates (see below Section 2.1), shown in Figure 1A; (Stage B) The use of biased (see below Section 2.2) MD simulations for the initial modeling of the heteromeric cross-interaction, as shown in Figure 1B; (Stage C) The use of conventional (see below Section 2.3) MD simulations for the refinement of the initial model and the analysis of the trajectory for the determination of biophysical properties, is shown in Figure 1C.

### 2.1. Selection of the Initial Templates and Refinement

For the particular amyloid cross-interaction between Aβ (1–42) and IAPP, structures PDB: 5OQV (64) and PDB: 7M65 (65) were selected from multiple available structures of Aβ and IAPP using two criteria: (i) We first considered Aβ (1–42), which contained all residues resolved, and IAPP structures that contained at least 6–37 residues resolved; the latter enabled the initial selection of at least 6 IAPP conformers for further consideration (Figure 1A). (ii) Subsequently, we calculated the backbone (Cα, C, N) Root Mean Square Deviation (RMSD) between the remaining Aβ–IAPP pairs of conformers (Table S1). Prior to RMSD calculations, the entire resolved backbone conformations were superimposed such that the 11–42 region of Aβ was aligned with the 6–37 region of IAPP without considering any gaps before or after these regions. The structural alignment was guided by sequence alignment performed using Clustal Omega(66) without gaps (e.g., high gap penalty), shown in Figure S1. The same criterion was used for all pairs for consistency.

**Figure 2:**
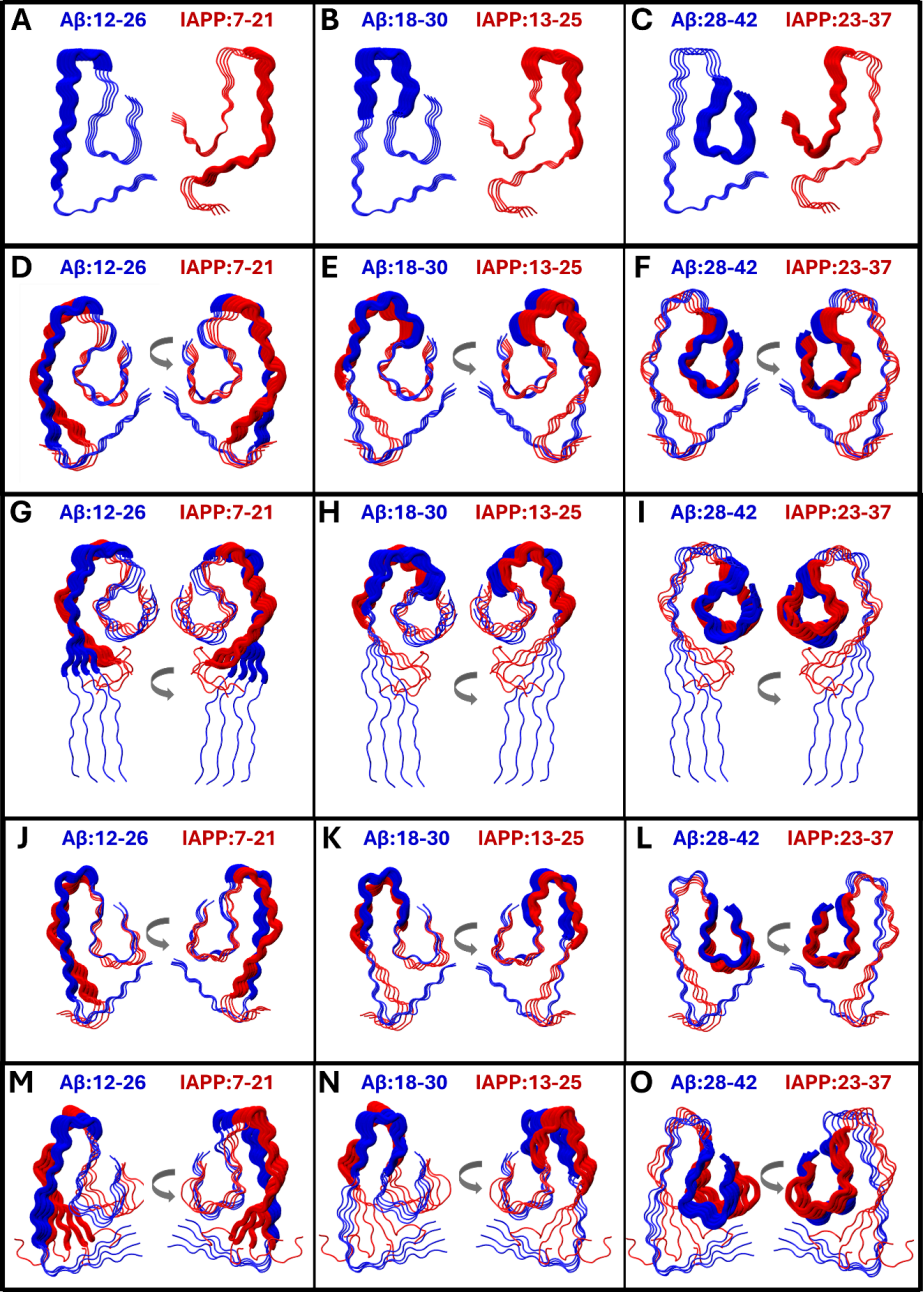
Panels A–C show the initial structures of the tetramer homomeric models of Aβ (left) and IAPP (right). Panels D–F show the structure of the *open* heteromeric conformer after the biased MD simulations, while panels G-I show the *principal open conformer* from the conventional MD simulations. Panels J–L show the structure of the *closed* heteromeric conformer after the biased MD simulations, while panels M-O show the *principal closed conformer* from the conventional MD simulations. Panels A–O show the initial homomeric structures and the heteromeric conformers, emphasizing partly overlapping regions Aβ 12–26 and IAPP 7–21 (panels A, D, G, J, and M), Aβ 18–30 and IAPP 13–25 (panels B, E, H, K, and N), and Aβ 28–42 and IAPP 23–37 (panels C, F, I, L, and O). For Panels D–O, different viewing angles are depicted, with Aβ on the front (left) or IAPP on the front (right). The backbone of the emphasized regions is shown in thick tube representation; blue for Aβ and red for IAPP, while the backbone of the nonemphasized regions is shown in thin tube representation; blue for Aβ and red for IAPP. The structures were visualized and captured using VMD.(68)

The Aβ tetramer (corresponding to four peptides in axial stacking from PDB: 5OQV (64)) was used in part to produce a heteromeric Aβ/IAPP fibril model. Additionally, using the same Aβ tetramer, we produced an octamer Aβ fibril model, which was used as the initial structure for the homomeric Aβ simulations, which served as a control for the Aβ fibril model. The IAPP tetramer (corresponding to four peptides in axial stacking from PDB: 7M65 (65)) was refined to produce the heteromeric Aβ/IAPP fibril model; in particular, the unresolved residues 1–5 from that PDB were generated by appending coordinates of IAPP residues 1–5 from PDB: 2L86 (67) using VMD.(68) This resulted in a refined IAPP tetramer with complete residues 1–37 and a disulfide bridge between residues 2 and 7. The complete IAPP tetramer was used with the aim to produce the heteromeric Aβ/IAPP fibril model. Additionally, using the refined IAPP tetramer, we produced an octamer IAPP fibril model, which was used as the initial structure for the homomeric IAPP simulations, which served as a control for the IAPP fibril model. In the computations (initial modeling and simulations), disulfide bridges between cysteine residues at positions 2 and 7 of each chain of IAPP were introduced, and the C-termini were amidated. In the case of the cross-interaction models, Aβ monomers are referred to as chains A-D, with A being the outermost external chain and D being the one to form the junction with IAPP. Similarly, IAPP monomers are referred to as chains E–H, with E being the one to form the junction with Aβ and H being the outermost external chain. In the case of the control runs, Aβ and IAPP monomers are termed chains A–H, with chains A and H being the outermost external monomers. The initial structures of the tetramer Aβ and IAPP fibril models are shown in Figure 2A–C, showing partly overlapping regions.

Additional details are provided in Supporting Information, S.2.1.

### 2.2. Biased MD Simulations Modeling the Heteromeric Cross-Interaction

The aim of this stage was to model the heteromeric cross-interaction between the two entities using short, biased MD simulations that could facilitate the heteromeric cross-interaction process. The short-biased MD simulations used two sets of constraints concurrently, which aimed to enhance the cross-interaction between the two entities, as shown in Figure 1B. The two sets of constraints were chosen to mimic, facilitate, and accelerate the potential naturally occurring cross-interaction process, allowing the cross-interacting entities to adapt their conformations and interactions with each other. The first set of constraints was prompted by the postulation that in a cross-interaction involving axial stacking, amyloid cross-β interactions exist throughout the structure, within the junction, and beyond. Thus, the first set of constraints comprised constraints between corresponding Cα:Cα atom pairs of neighboring monomers, both identical (i.e., homomeric) and within the junction (i.e., heteromeric). These constraints are referred to as “β-sheet-like” constraints, as they facilitated the formation of hydrogen bonding interactions within β-sheets. Within the junction, β-sheet-like constraints were introduced between corresponding Cα:Cα atom pairs of the 9–42 Aβ region and 4–37 IAPP region, as shown in Figures 1B and S1. In the consideration of β-sheet-like constraints, the N-terminal domain residues of IAPP (1–3) were not aligned with the Aβ N-terminal due to steric constraints imposed by the disulfide bridge between cysteine residues at positions 2 and 7 of IAPP. Furthermore, the second set of constraints was prompted by the postulation that in a cross-interaction (e.g., potentially resulting from cross-seeding), homomeric monomers away from the junction are nearly identical, while as approaching the junction, homomeric monomers would be perhaps less similar. Thus, the second set of constraints comprised six variations of shape-like similarity constraints, which were all applied independently and aimed to facilitate shape similarity across homomeric monomers, with different sets of force constants, variably reduced as approaching the junction, as shown in Figure 1B. The force constants of the shape-like similarity constraints for each variation are shown in Table S2.

We performed short, biased MD simulations, concurrently combining the first set and second set (comprising six variations, independently) of constraints. The input files were initially generated from CHARMM-GUI Implicit Solvent Modeler using the GBSW(73) implicit solvent model and were later modified accordingly. Upon completion of the six biased simulations, the β-sheet content within the junction and beyond was verified. Additionally, RMSD calculations were performed to compare the conformational properties of the Aβ and IAPP modeled fibrils, in different regions and overall, compared to the initial structures prior to the biased short simulations, enabling us to understand to what extent and how the particular simulations affected certain homomeric regions, independently. The final simulation structures from each of the six independent runs were extracted and used as starting conformations in longer (unbiased conventional) MD simulations, to allow further refinement and explore their properties.

Additional details are provided in Supporting Information, S.2.2.

### 2.3. Conventional MD Simulations of the Heteromeric Cross-Interaction and Analysis

We performed three simulations for every extracted structure from the biased simulations (six in total), as shown in Figure 1C, aimed at sampling different heteromeric conformers. These resulted in 18 simulations of 100 ns, which were performed without any use of constraints, aiming to further refine and explore the properties of the initially modeled systems. These simulations are referred to as “conventional,” as they comprise state-of-the-art MD simulations in explicit solvent with no constraints, with simulation input files generated by CHARMM-GUI Solution Builder.

We evaluated the degree of β-sheet content within the 18 trajectories of 100 ns each and observed that even in the absence of constraints, overall β-sheet interactions within the junction and beyond the junction were highly maintained during the simulations (Table S3). Small differences were observed across different runs, with the degree of β-sheet content varying per run and also with the N-terminal domain of Aβ being open-exposed or closed-contracted (Table S3). Subsequently, we identified the conformer(s) with the highest degree of β-sheet content within the junction and one or two peptides beyond the junction and selected the top two conformers, highlighted in yellow in Table S3. One of the selected conformers had an “*open*” Aβ N-terminal domain, while the other one had a “*closed*” Aβ N-terminal domain, and they are referred to as *open* and *closed* heteromeric conformers, respectively. The two runs of the selected conformers were investigated further, considering that they could correspond to two naturally occurring possibilities. The initial structures of these two runs (corresponding to the final configurations from the biased MD simulations they were derived from) are shown in Figure 2D–F,J–L. Upon selection of these conformers, their corresponding simulations were extended to 500 ns. The β-sheet content within both the junction and other chain pairs for the extended trajectories was verified. In addition to the aforementioned simulations investigating heteromeric cross-interactions, we performed three control runs investigating a homomeric Aβ modeled fibril and three control runs investigating a homomeric IAPP modeled fibril.

During the course of the simulation of the *open* heteromeric conformer, we observed the “opening” of the N-terminal domain occurring within the first 50 ns. Thus, the first 50 ns for both the simulations of the *open* and *closed* heteromeric conformers were considered as extra equilibration time. Simulation snapshots within the time window of 50–500 ns were used to perform clustering structural analysis, using Wordom with the quality threshold algorithm and a cutoff of 3.5 Å. This enabled us to determine the most representative conformers for both heteromeric models, corresponding to the cluster centers of the unique cluster in the case of the *open* heteromeric conformer, and the main─most populated cluster in the case of the *closed* heteromeric conformer. The cluster centers are referred to by us as the *principal open* and *closed* conformers and are shown in Figure 2G–I,M–O, respectively. Additionally, we calculated the hydrogen bond occupancies between atom pairs of neighboring chains, as well as the contact probabilities and the interaction free energy between residue pairs of neighboring chains. The hydrogen bond occupancies were calculated using VMD,(68) the contact probabilities were calculated using in-house FORTRAN scripts, while the interaction free energies were calculated using in-house CHARMM(69) scripts employing the GBMV implicit solvent model method II, as provided by CHARMM-GUI Implicit Solvent Modeler. The setup of calculations for the residue-pairwise interaction free energy followed previous studies in our lab.

Additionally, we performed several structural RMSD analyses. We compared the structures of Aβ and IAPP, individually, of the *principal open* and *closed* conformers to the structures of the biased MD simulations from which they originated (Section 2.2), as well as their initial/refined conformations (Section 2.1). Furthermore, we compared the structures of different chains of both Aβ and IAPP within the *principal open* and *closed* conformers. Moreover, we compared the structures of Aβ and IAPP of the *principal open* and *closed* conformers to experimentally resolved structures of both Aβ and IAPP. The aforementioned analyses were performed for different regions, which could also be partly overlapping, and different structural superpositions, which are provided in detail.

Additional details are provided in Supporting Information, S.2.3.

## 3. Results and Discussion

### 3.1. Formation and Refinement of the *Open* and *Closed* Heteromeric Conformers

Within the conventional MD simulations of the *open* heteromeric conformer, apart from the opening of the N-terminal domain observed in all Aβ peptides, occurring during the first 50 ns, the conformer’s structural properties remain highly preserved, and consequently all simulation snapshots fall within one cluster (containing all frames), the center of which─*principal open conformer*─is shown in Figure 2G–I. The opening of the N-terminal domain, which occurs in all Aβ peptides, results in large backbone RMSD values recorded for the simulation snapshots compared to the initial conformation, while the high-preservation of the conformation upon opening is reflected by the low backbone RMSD values recorded for the simulation snapshots compared to the average conformation of all simulation snapshots, as shown in Figure S2A,C,E,G. Within the conventional MD simulations of the *closed* heteromeric conformer, the conformer’s structural properties also remain highly preserved within the internal peptides, which is reflected by the low backbone RMSD values recorded for the simulation snapshots with regard to both the initial conformation as well as the average conformation, as shown in Figure S2B,D,F. In the case of a *closed* heterometric conformer, within the simulations, the outermost Aβ peptide (chain A) encounters partial detachment in the ∼1 to 10 N-terminal moiety (around ∼350 ns) and partial reattachment after ∼450 ns. This is potentially attributed to edge effects, as analogous partial detachment is also observed in the three control runs of Αβ (also extending to 500 ns). This partial detachment contributes to larger RMSD values when considering the outer peptides, as shown in Figure S2H. The main cluster of the *closed* heteromeric conformer comprises 78% of the total snapshots/conformations, and the *principal closed* conformer is shown in Figure 2M–O. The second and third clusters (∼22%) comprise snapshots/conformations primarily during the period of the peptide partial detachment. Interestingly, within the simulations of the control homomeric Aβ fibril, an opening of the N-terminal domain in any simulation has not been observed, at least within the simulation duration, in contrast to the opening observed in the *open* heteromeric conformer. This could potentially indicate that the opening may be partly attributed to less stability conferred by the IAPP N-terminal domain. The structures of *principal open* and *closed* conformers are provided as supporting material in the PDB (CHARMM) format.

We decomposed the structures into three partially overlapping regions to better explain the changes induced by biased and conventional MD simulations. Backbone RMSD calculations were used to compare the initial conformers to the conformers resulting from biased MD simulations, as well as the *principal open* and *closed* conformers from the conventional MD simulations. These calculations were performed individually for Aβ and IAPP and enabled us to uncover the importance of each of the two types of simulations, in relevance to their effect on altering the two conformers and also differentiating between alterations in certain regions of the conformers (Tables S4 and S5). Upon completion of the stage of biased MD simulations, major changes, especially in the core and C-termini of the fibrils (e.g., regions 18–30, 28–42 of Aβ, respectively, and regions 13–25, 23–37 of IAPP) were induced for both Aβ and IAPP of the *open* heteromeric conformer (Figure 2E,F), and primarily IAPP of the *closed* heteromeric conformer (Figure 2K,L) in comparison with their respective initial structures (Table S4A,B). Additionally, changes in the same regions, predominantly for Aβ in the *open* heteromeric conformer, were induced in the stage of conventional MD simulations (Tables S4A,B and S5A,B), as shown in Figure 2H,I. The key changes between conformations after biased MD simulation to the *principal open* and *closed* conformers occur within the N-terminal domain of the *open* heteromeric conformer, due to its opening (Tables S4A,B and S5A,B), as shown in Figure 2G–I. The *principal open* and *closed* conformers are different primarily in the N-terminal domains of Aβ and IAPP (Table S6A,B), as shown in Figure 2G–I,M–O. This is due to the fact that while there is no such opening of the N-terminal domain of IAPP, the opening of Aβ in the *open* conformation affects the N-terminal domain of IAPP too. The different conformations of the termini between the *principal open* and *closed* conformers have an effect on the superposition, resulting in relatively large structural deviations for individual regions, too, between open and closed regions. Yet, when considering superposition in each of the different regions individually, it is evident that the open and closed states are to some extent structurally similar in different regions beyond their termini (Table S6A,B). The changes induced by the biased MD simulations enabled the two entities to significantly adapt to each other, while alterations during the stage of conventional MD simulations also played a role in the refinement of the structures, inducing changes primarily to the termini, especially for the *open* heteromeric conformer.

Figure S3A,B present the number of backbone–backbone hydrogen bonds at the junction as a function of time during the biased and conventional MD simulations, respectively, for both the *open* and the *closed* heteromeric conformers. Notably, within the biased MD simulations, the combination of two types of constraints results in the rapid formation of hydrogen bonding interactions. Additionally, the number of hydrogen bonds further increases within the *open* heteromeric conformer while it remains constant overall for the *closed* heteromeric conformer. A detailed biophysical characterization of the interactions formed is provided below.

### 3.2. Biophysical Characterization of *Op* *en* and *Closed* Heteromeric Conformers

**Figure 3:**
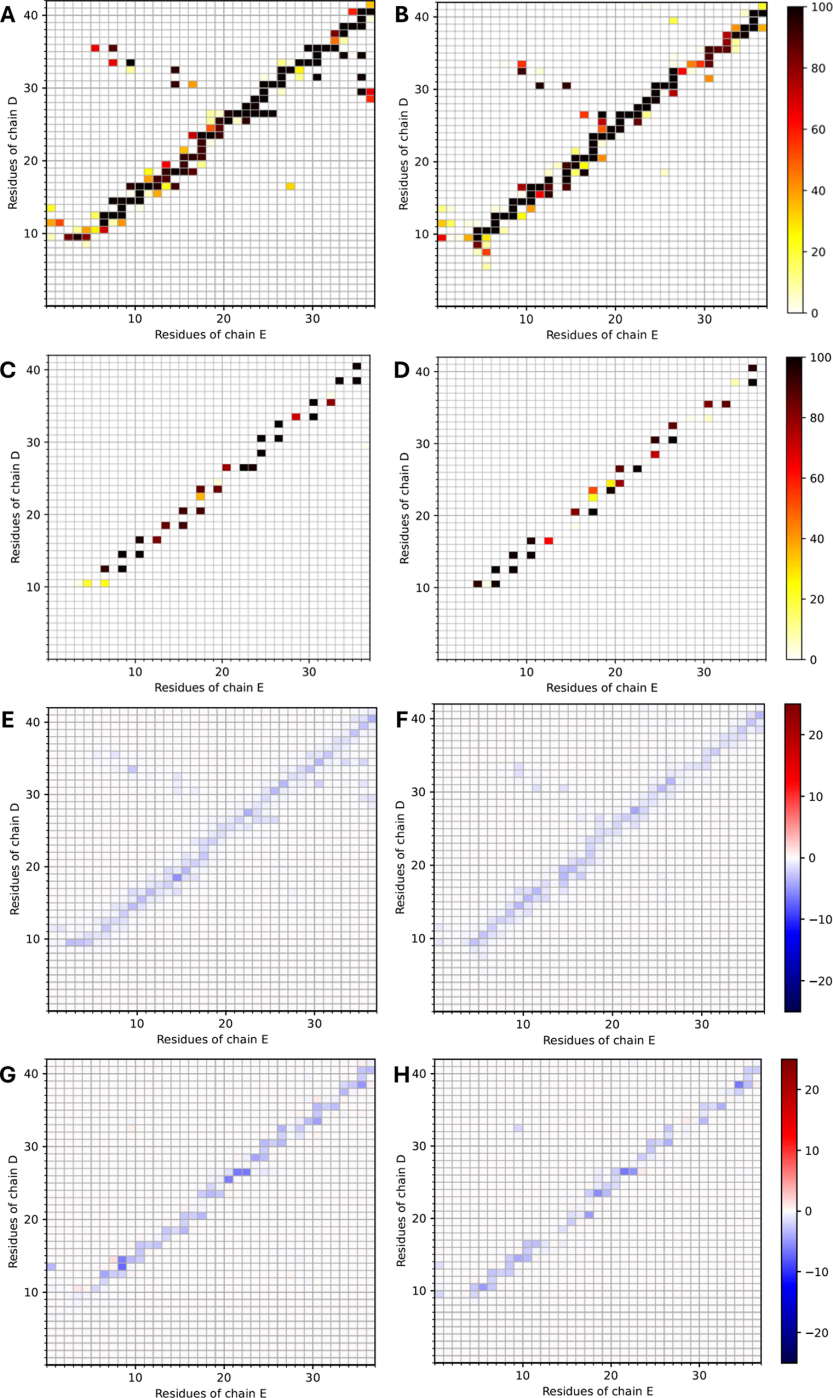
Panels A−B, C−D, E−F, and G−H show contact maps, backbone–backbone hydrogen-bond maps, nonpolar interaction free energy maps, and polar interaction free energy maps, respectively, between chains D and E (junction) of the two heteromeric conformers. Panels on the left correspond to the *open* heteromeric conformer, while panels on the right correspond to the *closed* heteromeric conformer. The values in panels A–D correspond to percentage probabilities/occupancies, while the values in panels E–H correspond to interaction free energies in kcal/mol.

**Figure 4:**
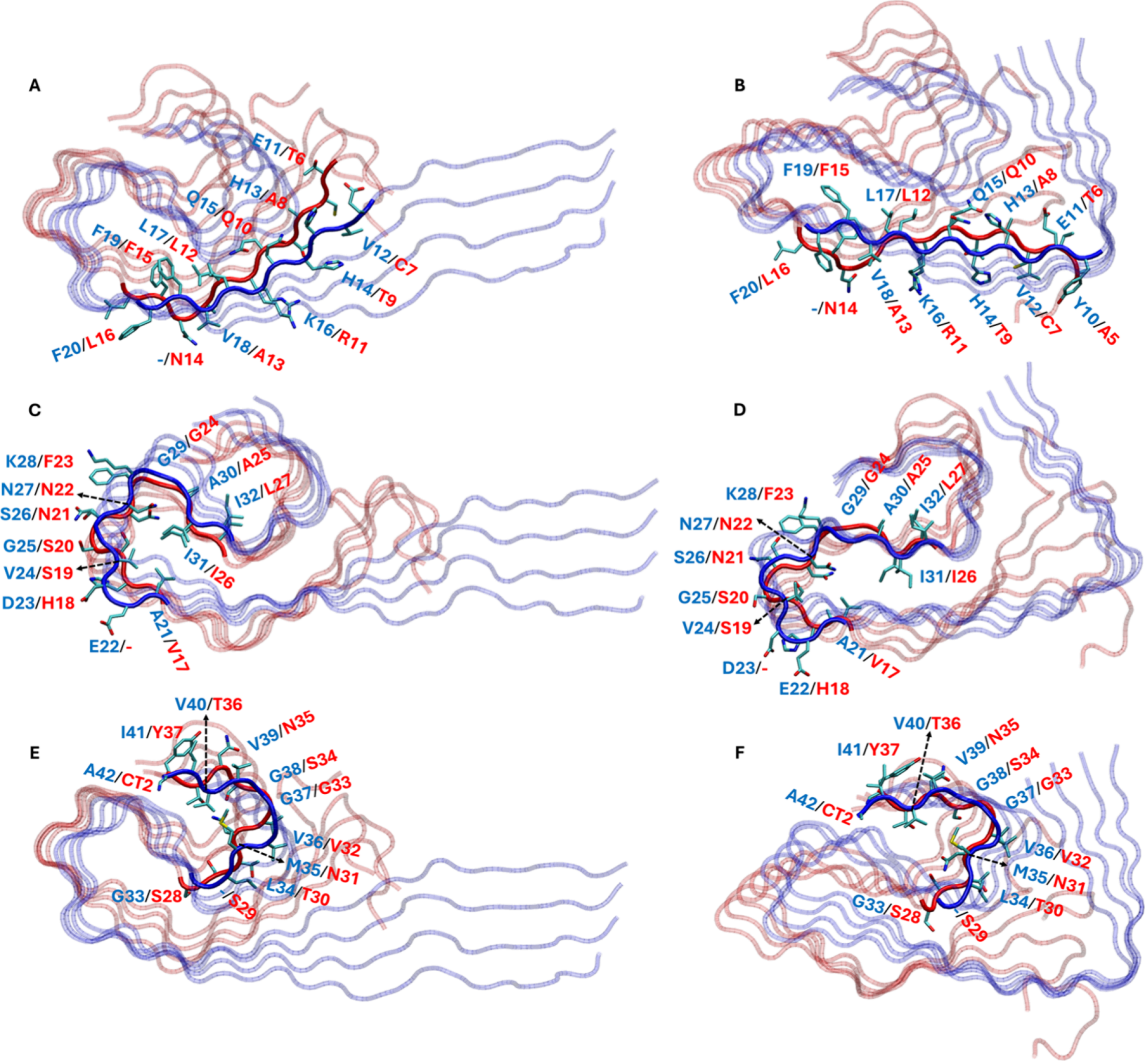
Panels on the right (A, C, and E) show the *principal open conformer*, while the panels on the right (B, D, and F) show the *principal closed conformer*. Panels A and B emphasize the regions 10–20 for Aβ and 5–16 for IAPP. Panels C and D emphasize the regions 21–32 for Aβ and 17–27 for IAPP. Panels E and F emphasize the regions 33–42 for Aβ and 28–37 for IAPP. The labels in the emphasized regions indicate the residues of Aβ (blue font) and IAPP (red font) that have complementary side chains. The backbone of the emphasized regions is shown in opaque tube representation; blue for Aβ and red for IAPP, while the backbone of the nonemphasized regions is shown in transparent tube representation; blue for Aβ and red for IAPP. The side chains in the emphasized regions are shown in licorice representation and are colored by name, while the hydrogen atoms are omitted for clarity. The structures were visualized and captured using VMD.(68)

**Figure 5:**
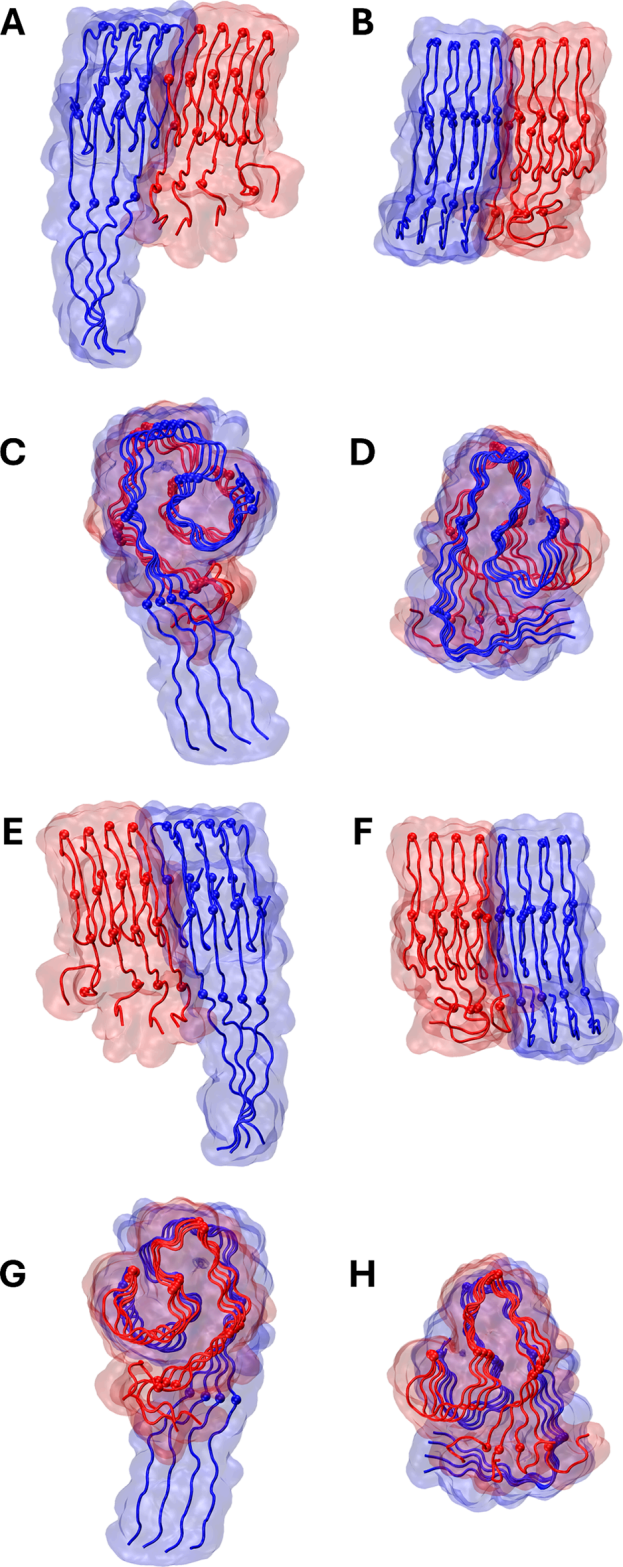
Panels on the left (A, C, E, and G) and on the right (B, D, F, and H) show the steric zipper of the *principal open* and *closed conformers*, respectively, in different viewing angles. The backbone of the structures is shown in tube representation; blue for Aβ and red for IAPP. The side chains of the structures are shown in Glass I–QuickSurface representation; blue for Aβ and red for IAPP. The Cα atoms of residues V12, V18, G25, I31, and I41 of Aβ and the Cα atoms of residues C7, A13, S20, I26, and Y37 of IAPP are colored in blue and red, respectively, and shown in vdW representation. The structures were visualized and captured using VMD.(68)

In both *open* and *closed* heteromeric conformers, we observe a nearly exceptional β-sheet hydrogen bonding (Figure 3) and side chain complementarity (Figure 4) within the junction of the two heteromeric conformers, and beyond. In other words, our results suggest the presence of an amyloid steric zipper spanning nearly throughout the structure, both in the individual heteromeric conformers and in the junction, as shown in Figure 5. We consider that this is one of the key novelties resulting from the approach followed, demonstrating that Aβ–IAPP can form favorable interactions, as reflected by their corresponding polar and nonpolar interaction free energies spanning from 10 to 41 for Aβ and 7 to 37 for IAPP (Figure 3). The N-terminal moiety of IAPP did not participate in β-sheets with the N-terminal domain of Aβ, presumably due to steric constraints imposed by the disulfide bridge between cysteine residues at positions 2 and 7 of IAPP. In the simulations of the *closed* model, we observed contacts between a portion of residues of the 2–7 moiety of IAPP and the corresponding region of Aβ (Figure 3), which probably enhanced the stability of the *closed* heteromeric conformer. On the contrary, such contacts were not observed in the simulations of the *open* heteromeric model upon opening.

The biophysical analysis focuses on 1 ns simulation increments of the main clusters of the *open* and *closed* heteromeric conformers’ simulations and inner chains B-G. Side chain misalignments in positions IAPP:N14 in both *open* and *closed* conformers, Aβ:E22 in the *open* and Aβ:D23 in the *closed* conformer, and additionally IAPP:S29 in both *open* and *closed* conformers were observed (Figure 4). The complementary interactions within the junction are presented in Figure 4, and the sequence superposition in the junction is shown in Figure S4. Notably, out of the three misalignments that are observed in the junction, two are common for both *open* and *closed* heteromeric conformers, and one is different, as shown in Figure S4. Apart from these side chain misalignments in the junction, β-sheet hydrogen bond interactions exist between residue moieties 12–17, 19–21, 23–24, 26–32, 34–36, and 38–41 of Aβ and 7–12, 15–17, 18–19, 21–27, 30–32, and 34–37 of IAPP, respectively, for the *open* heteromeric conformer. Similarly, β-sheet hydrogen bond interactions exist between residue moieties 10–17, 20–21, 24–27, 29–32, 35–36, and 39–41 of Aβ and 5–12, 16–17, 19–22, 24–27, 31–32, and 35–37 of IAPP, respectively, for the *closed* heteromeric conformer.

Beyond the junction and excluding any edge effects on the exterior chains, β-sheet hydrogen bond interactions between neighboring chains exist nearly throughout residue moieties 1–42 of Aβ and 6–37 of IAPP for both the *open* and *closed* heteromeric conformers. Overall, these are reflected both in the contact maps (Figure 3A,B), hydrogen bond maps (Figure 3C,D), as well as in the residue-pairwise polar and nonpolar interaction free energy maps (Figure 3E–H). Notably, the β-sheet hydrogen bonds along with the side chain contacts in the junction contribute to an amyloid steric zipper spanning nearly the entire structure, including the junction and beyond, contributing to the stabilization of the two conformers.

Interactions between side chains of corresponding Aβ–IAPP residue pairs in the junction for the *open* and *closed* heteromeric conformers contribute significantly to the stability. These interactions occur between identical as well as mostly nonidentical residues, which interact predominantly through their corresponding nonpolar moieties (Table S7A,B). In addition to nonpolar interactions stabilizing the side chains of the junction and beyond (Figure 3E,F), particular side chain atoms are hydrogen bonded to other side chain or main-chain atoms both in the junction and between neighboring chains in the homomeric regions (Tables S7A,B and S8). Side chain-side chain or side chain-backbone hydrogen bonds between atom pairs of residues paired in β-sheets additionally contribute to polar interactions (Figure 3G,H). These include hydrogen bonds in the junction of the *open* heteromeric conformer between H14(ND1) of Aβ and T19(OG1) of IAPP, S26(OG) of Aβ and N21(O) of IAPP, N27(ND2) of Aβ and N22(OD1) of IAPP, and I41(O) of Aβ and Y37(NT-amide) of IAPP (Table S8). Similarly, these include hydrogen bonds in the junction of the *closed* heteromeric conformer between Q15(OE1) of Aβ and Q10(NE2) of IAPP, V24(O) of Aβ and S19(OG) of IAPP, N27(ND2) of Aβ and N22(OD1) of IAPP, V39 (N) of Aβ and N35(OD1) of IAPP, and I41(O) of Aβ and Y37(NT-amide) of IAPP (Table S8). Additional side chain-side chain or side chain-backbone interactions are formed between atoms in residue pairs nonpaired in β-sheets, further contributing to the stability of the heteromeric conformers (Table S8). These include Q15(NE2) of Aβ and T9(O) of IAPP, G25(O) of Aβ and S19(OG) of IAPP, and N27(ND2) of Aβ and A25(N) or A25(O) of IAPP, for the *open* heteromeric conformer, as well as N27(N) of Aβ and S19(OG) of IAPP, G33(O) of Aβ and Q10(NE2) of IAPP, and L34(O) of Aβ and N31(ND2) of IAPP, for the *closed* heteromeric conformer.

Furthermore, additional contacts between residues of neighboring chains, which are not sequentially proximal to each other in the homomeric regions (Figures S5A, S6A, S7A, S8A, S5B, S6B, S7B, and S9B), or sequentially proximal to corresponding residues in the junction, contribute to the stabilization of the two conformers (Figure 3A,B). Interestingly, in the case of the *closed* heteromeric conformer, additional contacts are observed between the N-terminal and C-terminal domains of neighboring chains of Aβ in the homomeric region, attributed to the fact that the N-terminal domain of Aβ remains in close proximity to the C-terminal domains (Figures S5B and S6B). Overall, cross-interaction contacts between Aβ and IAPP residues, which are not sequentially proximal comprise─in part─a combination of contacts formed between the corresponding homomeric Aβ and IAPP.

Interestingly, the cross-interaction between the two entities does not appear to negatively affect the β-sheet hydrogen bonding and the side chain complementarity beyond the junction (Figures S5–S8; Panels A–D), as interactions beyond the junction are highly preserved. This is also reflected by the highly favorable polar and nonpolar residue-pairwise interaction free energies of the interacting residues both in the junction (Figure 3E–H) and beyond (Figures S5–S8; Panels E–H). This suggests that cross-interactions in both conformers do not appear to compete or negatively affect homomeric interactions, but rather, it can be viewed as an interaction coexisting to a significant extent in harmony with homomeric assemblies beyond the junction.

**Figure 6:**
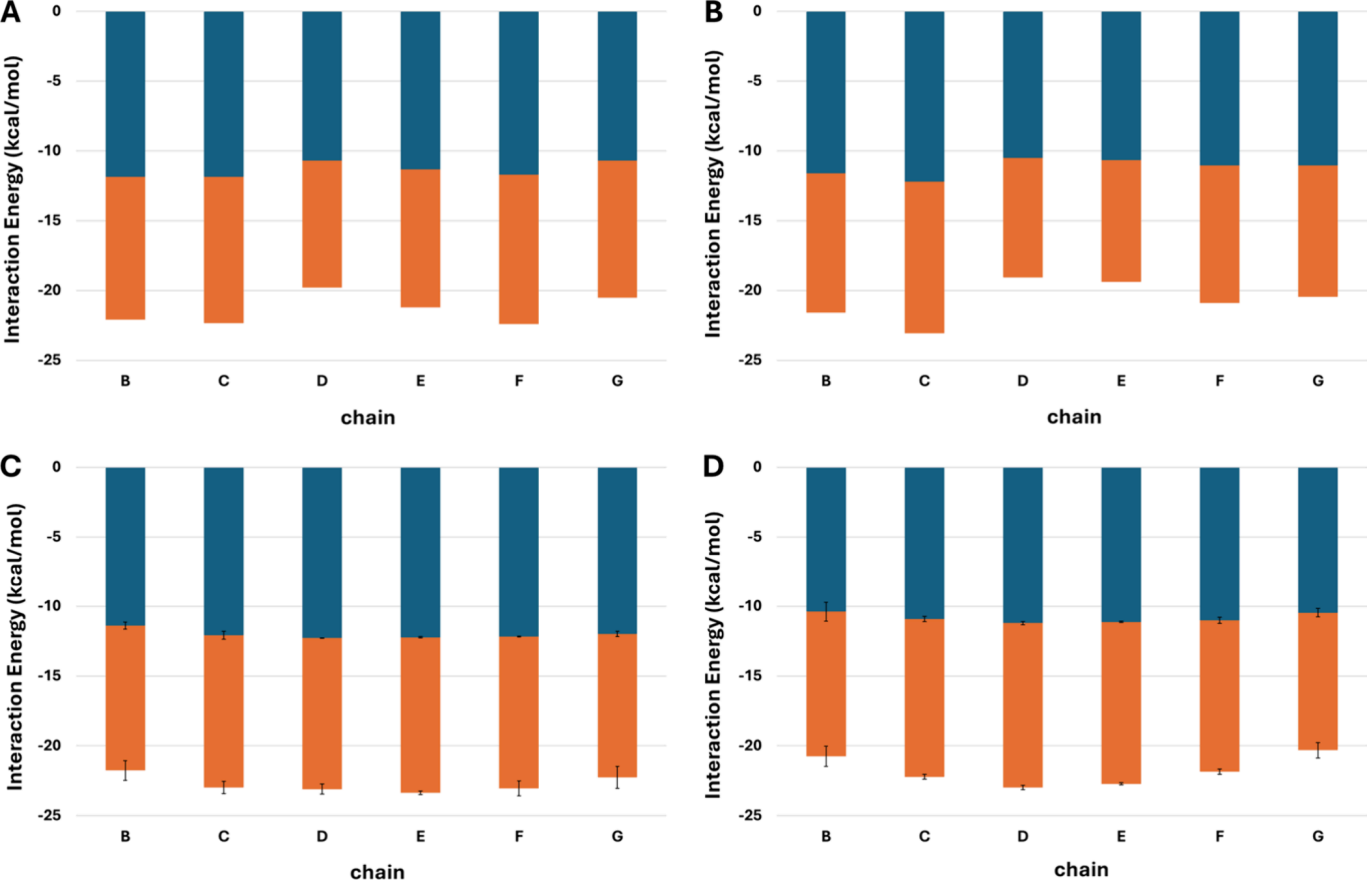
Panels A–D show the residue-normalized interaction free energy of chains B–G, for the *open* heteromeric conformer, the *closed* heteromeric conformer, the homomeric Aβ control, and the homomeric IAPP control, respectively. Residue-normalized interaction free energy is decomposed into polar and nonpolar contributions, shown in orange and blue, respectively. The values demonstrated for the controls are averages over the triplicate runs, and error bars denote the standard deviation.

The residue-pairwise interaction free energy values were summed for every residue, considering its interactions with other residues in neighboring chains. Subsequently, we summed up all interaction free energy values of every residue per chain and then divided by the total number of residues per chain to compare evenly between Aβ and IAPP peptides in the *open* and *closed* heteromeric conformers (Figure 6). This residue-normalized interaction free energy aimed to evenly compare interaction energies per chain, in an “independent of molecule size”(1) manner. Additionally, residue-normalized interaction free energy values were compared to the control simulations of homomeric octamers of Aβ and IAPP. Edge peptides were omitted due to the fact that they have only one neighboring chain, and additionally, understanding any edge effects is beyond the scope of our investigation. Chains B and C of Aβ have comparable residue-normalized interaction free energy in the heteromeric vs homomeric control runs, and similarly, chains G and F of IAPP have comparable residue-normalized interaction free energy in the heteromeric vs homomeric control runs. This agrees with the aforementioned observation on β-sheet hydrogen bonds and contacts on the fact that interactions beyond the junction are preserved, depicting that the cross-interaction does not have a negative impact on the interaction free energy between Aβ homomeric chains, as well as IAPP homomeric chains. Thus, there seems to be minimal energetic penalty for homomeric assemblies beyond the junction due to the cross-interaction. However, there is a small penalty due to cross-interactions in the junction which appears to be slightly higher for Aβ to IAPP (∼1.8 kcal/mol and ∼0.7 kcal/mol in the *open* and *closed* conformers, respectively); this is reflected by the comparison between *open* and *closed* heteromeric conformers in the junction, in reference to the particular corresponding homomeric conformers in control runs (Figure 6). Additionally, this complies with the fact that in both *open* and *closed* heteromeric conformers, IAPP in the junction (chain E) has a residue-normalized interaction free energy closer to that of IAPP beyond the junction (chains F and G) in comparison to that of Aβ in the junction (chain D) and to that of Aβ beyond the junction (chains B, C). This suggests that IAPP in the junction may participate in cross-interactions with an Aβ fibril nearly as favorably as with an IAPP fibril. Notably, Aβ in the junction may be less favorable to participate in cross-interactions with an IAPP fibril in comparison to an Aβ fibril.

### 3.3. Comparison of Heteromeric Conformers to Homomeric Polymorphs

The following analysis is performed on the *principal open* and *closed* conformers and focuses on their key overlapping regions 12–42 of Aβ and 7–37 of IAPP. Overall, for both heteromeric conformers, the RMSD between homomeric monomers increases with neighboring distance, and interestingly does not exceed 2.0 Å, as shown in Table S9A,B, with the exception of a single pair: chain E of IAPP to chain H of IAPP, of which its value is equal to 2.3 Å, as shown in Table S9B. The above denotes that the difference in the structures of the homomeric monomers within the heteromeric conformers does not significantly vary, and small differences stem from the fact that the peptides in the junction tend to adapt more toward improving interactions with their heteromeric neighbor. Interestingly, our study depicts that the two heteromeric peptides in the junction of the *principal open* and *closed* heteromeric conformers vary by 2.1 and 2.6 Å, respectively, as shown in Table S9A,B, which are also relatively low, especially when considering that the two peptides are different. This further suggests that their cross-interaction property in the axial stacking can be attributed to their ability to adapt to each other, adopting a conformation that will allow favorable interactions in the junction. Earlier observations suggest that IAPP may have a higher propensity to adapt, and thus adaptation may be primarily attributed to IAPP, which also has overall higher structural differences to its corresponding monomers compared to Aβ, in both *principal open* and *closed* heteromeric conformers, as shown in Table S9A–C. Notably, as expected, the structural differences between heteromeric peptides, Aβ and IAPP, are notably higher compared to homomeric peptides and increase with neighboring distance but do not exceed ∼3 Å in the *principal open* and ∼4 Å in the *principal closed* heteromeric conformers, as shown in Table S9A,B, respectively.

In what follows, we assessed the degree of similarity between tetramers of Aβ and IAPP in both *principal open* and *closed* heteromeric conformers to polymorphic configurations of both Aβ and IAPP, questioning to what extent Aβ and IAPP in our heteromeric conformations resemble experimentally resolved polymorphs. The relatively reasonable similarity between the Aβ and IAPP tetramer homomeric peptides individually in both the *principal open* and *closed* heteromeric conformers was a key factor enabling this assessment. Driven by the fact that our aforementioned analysis showed that in the junction, Aβ and IAPP peptides show a tendency to adapt to each other, we questioned whether cross-interaction could be possible at least partly due to the ability of Aβ and IAPP to adopt polymorphs resembling each other, enabling and favoring the interaction between the two. In this endeavor, we considered investigating similarity only in regions 18–42 of Aβ and the corresponding 13–37 of IAPP. The selection of regions was based on the fact that if the region was expanded to include more residues (e.g., 6–37 of IAPP), a significantly smaller number of IAPP polymorphs could be considered and would limit the number of structures considered for the investigation of this important question.

**Figure 7:**
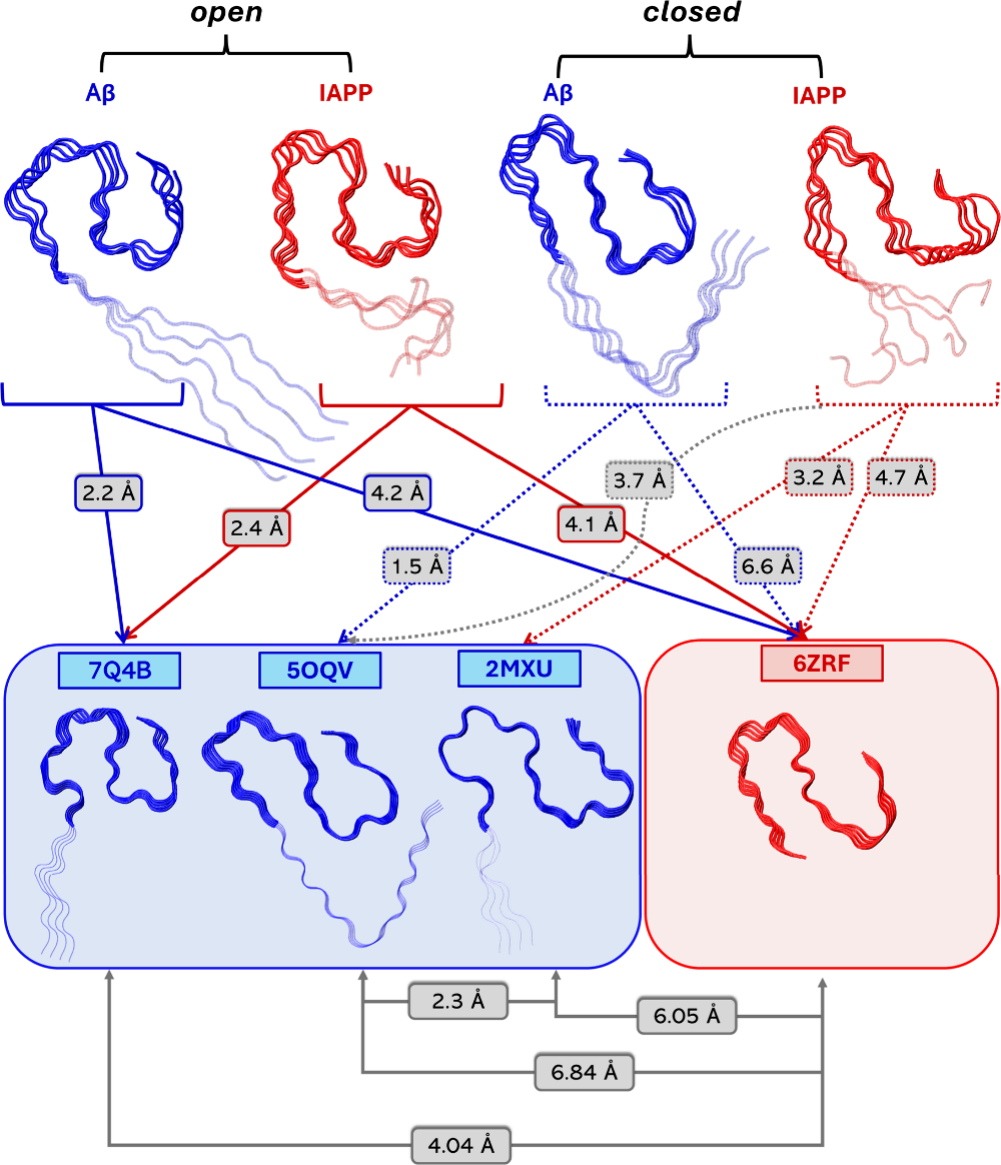
Aβ and IAPP entities of the *principal open* and *closed* heteromeric conformers were superimposed in the regions 18–37 for Aβ, and 13–37 for IAPP, with experimentally resolved structures of Aβ and IAPP. Shown here are the experimentally resolved Aβ and IAPP structures with the lowest backbone RMSD relative to the corresponding homomeric entities of the *principal open* and *closed* heteromeric conformers, with the exception of the additional comparison of IAPP in the *principal closed* heteromeric conformer to PDB: 2MXU.(86) The respective RMSD values are indicated for each comparison. Additionally, the RMSD between the experimentally resolved structures themselves is also shown, as well as the RMSD between the IAPP of the *closed* heteromeric conformer in comparison with 5OQV. The backbone of the superimposed regions is shown in opaque tube representation; blue for Aβ and red for IAPP, while the backbone of the nonsuperimposed regions is shown in transparent tube representation; blue for Aβ and red for IAPP. The structures were visualized and captured using VMD.(68)

A comparative analysis of the homomeric tetramers in the *principal open* and *closed* heteromeric conformers to the experimentally resolved structures of Aβ and IAPP showed that the cross-interacting conformations of Aβ and IAPP share significant similarities with particular experimentally resolved polymorphs in the region 18–42 of Aβ and 13–37 of IAPP (Figure 7 and Table S10). Interestingly, in the *principal open* heteromeric conformer, there is a striking similarity between both the Aβ tetramer (∼2 Å) and the IAPP tetramer (∼2 Å) with Aβ PDB: 7Q4B,(84) as shown in Figure 7. Interestingly, the similarity of the IAPP tetramer in the *principal open* heteromeric conformer with Aβ PDB: 7Q4B (84) is higher than its more similar IAPP conformer PDB: 6ZRF (85) (∼4 Å), with the difference between PDB: 7Q4B (84) and PDB: 6ZRF (85) equal to ∼4 Å, as shown in Figure 7. This suggests that the ability of Aβ to adopt the particular polymorph PDB: 7Q4B,(84) along with the capacity of IAPP to adopt the particular polymorph PDB: 6ZRF (85) (which is ∼4 Å different from 7Q4B(84)), as well as the ability of Aβ and IAPP tetramers to further adapt to each other (with IAPP showing higher adaptability), is a key factor for the cross-interaction between the two, at least in the particular *principal open* heteromeric conformer. Analogously, in the *principal closed* heteromeric conformer, there is a striking similarity between the Aβ tetramer (∼1.5 Å) with Aβ PDB: 5OQV.(64) IAPP tetramer is most similar to Aβ PDB: 2MXU (86) (∼3 Å) and Aβ PDB: 5OQV (64) (∼4 Å), in comparison to IAPP PDB: 6ZRF (85) (∼5 Å). Notably, the difference between Aβ PDB: 2MXU (86) (∼3 Å), and Aβ PDB: 5OQV (64) is relatively low (∼2 Å). This suggests that the ability of Aβ to adopt the particular polymorph PDB: 5OQV,(64) along with the capacity of IAPP to adopt a conformation somewhat similar to polymorphs 5OQV(64) and 2MXU,(86) is a key factor for the cross-interaction between the two, at least in the particular *principal closed* heteromeric conformer. The aforementioned results highlight the importance of polymorphism in cross-interactions, as axial stacking cross-interactions could potentially occur due to the ability of the two entities to adopt different conformations and also adapt in the presence of each other, and in the particular case, IAPP seems to have a higher tendency to adopt Aβ-like conformations. Nevertheless, this needs additional investigation and can be solidified with the potentially experimental resolution of additional IAPP polymorphic structures.

## 4. Concluding Remarks

Understanding amyloid cross-interactions, related to amyloid cross-seeding and/or amyloid coaggregation, holds key importance in biology and diseases. Interactions between amyloid proteins comprise the basis of several pathogenic pathways.(87) Amyloid cross-interactions have been significantly underexplored compared to homoamyloid aggregation. Although different experimental and computational methods can provide distinct information on amyloid cross-interactions, notably, no method can fully capture the complexity of this process, at least at the moment.(87)

Heterotypic aggregation is associated with major challenges for current structure determination techniques. According to Baghel and Ghosh, while nuclear magnetic resonance (NMR) spectroscopy and cryo-electron microscopy (cryo-EM) can be used to derive high-resolution structures, these methods need a degree of homogeneity within the aggregation ensemble, which may inherently not be present in heteromeric ensembles, and thus, it is impossible to determine a priori.(36) As for in silico approaches, AmyloGraph is the first database developed on amyloid interactions, which has facilitated the development of tools that predict how different amyloids might interact. PACT uses Modeler-based molecular threading to assess cross-interaction potential. AmyloComp evaluates the general cross-seeding potential and also estimates the structural compatibility of sequences for heterofibril formation. Despite the valuable knowledge gained by both predictive approaches, deriving a precise understanding of how two amyloids cross-interact has remained a challenge.(87) For example, the predicted structures produced by PACT for any cross-interaction assume a particular homomeric conformer of IAPP, under the assumption that most of the currently known amyloid fragments, which are longer than a few amino acids, share a similar beta-sheet turn architecture, commonly known as the beta arch.(59) Nevertheless, this could at least partly limit the derivation of highly accurate models for a diversity of amyloid cross-interactions.

Fundamentally, understanding amyloid cross-interactions, either associated with cross-seeding or coaggregation, could be decomposed into two problems: elucidating the mechanisms of the process of cross-seeding or coaggregation and elucidating the structural and biophysical properties of the formed cross-interaction. Here, we developed a computational approach combining biased and conventional MD simulations addressing the latter. Notably, our approach has the capacity to facilitate and accelerate the process, overcoming at least in part limitations of computational approaches using conventional MD simulations only. Inherently, our approach does not address the former, and it comprises certain limitations, which predominantly are associated with the lack of information on the process it takes for such structures to be formed, either through cross-seeding or even coassembly. Notably, it is possible that the particular studied pattern may be less possible over other more predominant mixed patterns that could result from coaggregation; as such, our computationally derived structures may correspond to models more likely to result from cross-seeding. An additional relevant limitation is the fact that our approach starts by assuming the formation of a particular pattern and does not examine different amyloid cross-interacting axial stacking patterns or even lateral stacking patterns. However, our approach could pave the way for future studies studying both axial and lateral stacking patterns, as both can be concurrently present in heteromeric interactions. Nevertheless, our approach can pave the way toward the examination of different cross-interacting patterns, e.g., alternating Aβ–IAPP peptides, especially in axial stacking, extending the scope of the current study. Notably, instead of using conventional methods only, such as all-atom simulations or enhanced sampling techniques, we went beyond the current methods commonly considered in molecular simulations to study amyloid cross-interactions. This falls within the notion that going beyond what is commonly considered in molecular simulations can probe effects and facilitate understanding of assembly properties(107) that may be challenging to do otherwise. Furthermore, upon derivation of a diversity of potential cross-interacting models, thermodynamic thought pathways of formation can be studied to delineate the potential naturally occurring formation mechanisms.(102)

Our computationally derived structures demonstrate the capacity of Aβ and IAPP to cross-interact by forming favorable interactions between each other (i.e., in the junction) and beyond (i.e., with their corresponding homomeric peptides), which is at least relevant to their capacity to cross-interact. It is important to note that the possibility of the two peptides to form favored elongated cross-interactions was also initially suggested to the best of our knowledge by studies of Zheng et al.(26) Nevertheless, we consider that the development of the computational approach in this study was a critical step enabling us to examine and demonstrate the computationally predicted high degree of compatibility in β-sheet interactions and side-chain contacts in the junction and beyond. Overall, this results in an amyloid steric nearly throughout the amyloid-cross-interacting structures. Additionally, our results from structural analysis and in part from energetic analysis suggest that an Aβ fibril can be a seed for IAPP rather than the opposite. In this context, our results suggest that (i) IAPP in the junction can cross-interact with an Aβ fibril nearly as favorably as with an IAPP fibril; (ii) at least for the polymorphs examined, while both Aβ and IAPP adapt to each other in the junction, IAPP has a higher propensity to adopt a polymorph that is formed by homomeric Aβ. In this context, additional investigations are needed to solidify this, potentially exploring additional IAPP polymorphs. Importantly, our results comply with the fact that polymorphism is highly interrelated with amyloid cross-interactions, which is in line with previous observations.(26) Additionally, the *principal open* and *closed* heteromeric conformers presented share significant similarities but also interestingly suggest that the N-terminal domain of Aβ can be either extended or not, respectively. Particularly, we suggest that the capacity of amyloids to adopt different polymorphs, and the types of polymorphs they can adopt, determine and drive their cross-interacting capacities; this is valid at least for the cross-interaction investigated here, but could potentially hold across other amyloid cross-interactions. In this context, we consider axial stacking cross-seeding is presumably dependent on the capacity of two entities to adopt polymorphs of high shape similarity. Thus, the analytical investigation and consideration of different polymorphs should be considered critical in the study of cross-interactions in axial stacking.

Interestingly, the cross-interacting polymorphs of either Aβ or IAPP within the *principal open* and *closed* conformers are in the range of ∼2 to 3 Å of polymorphs that Aβ has been resolved, at least in the region investigated. Nevertheless, it is important to note that they can vary significantly from other polymorphs of Aβ. Therefore, the cross-interacting polymorph(s) that each of the two peptides has the capacity to adopt, appears to be closer to the ones that homomeric Aβ can adopt, at least considering the current experimentally resolved structures of Aβ and IAPP fibrils. In this context, we consider that our results also comply with Baghel and Ghosh's findings on the coaggregation of the peptides from the monomeric stage leads to the formation of unique polymorphs,(36) taking into consideration that the comparison of spectra was performed for individual unique polymorphs of each of the two peptides in that study. Furthermore, the authors showed that seeding with preformed IAPP fibrils leads to aggregates similar to native Aβ.(36) This could partly be attributed to the lower favorability of IAPP to serve as a seed, while the presumably little contribution of IAPP in the aggregates,(36) in conjunction with IAPP's capacity to adjust to Aβ according to our findings, could lead to the formation of conformations resembling significantly the Aβ fibril conformation.(36) Importantly, due to the abundance of experimental studies investigating the cross-interaction between Aβ and IAPP, we consider that the choice of the particular amyloid cross-interacting system has played a key role in both partially validating our results and additionally predicting properties and structures that can be validated in the future by experimentalists.

It is important to note that the novelty of our findings on studying the particular cross-interaction could be attributed to the combination of the stage of biased MD simulations in conjunction with conventional MD simulations. Our findings clearly indicate the role of both stages, while it is important to highlight the efficiency of the biased MD simulations stage, which should be largely attributed to the combined use of constraints, facilitating and accelerating the potential naturally occurring cross-interaction. We consider that our approach can be significantly improved in many ways, including but not limited to the investigation of several patterns of cross-interactions and several possibilities of matching residue pairs in the cross-interactions, using a combination of both sequence and shape similarity between the two components. Notably, our approach’s simplicity suggests its capacity to be applied in the study of a series of cross-interacting systems beyond the example case investigated in this study, both in the field of health and biology, as well as for biomaterial applications. These can include (i) studying cross-seeding of polymorphs of the same protein, e.g., α-synuclein fibril elongation by self-seeding or cross-seeding of wild-type or mutant α-synuclein with wild-type or mutant α-synuclein seeds,(110) or seeding and cross-seeding reactions between two unique fibril polymorphs,(111) (ii) studying the interplay between α-syn and tau and understanding their synergistic roles in driving neurodegeneration through cross-seeding and potential prion-like propagation, (iii) studying of α-synuclein with prion-like C-terminal domain of TDP-43 which interact synergistically to generate neurotoxic hybrid fibrils,(118) and (iv) cross-seeding of alpha-synuclein aggregation by amyloid fibrils of food proteins.(119)

## Supplementary Material









## Supporting Information

Additional details regarding the computational methodology, additional figures and tables used to support our results, discussion and conclusion (PDF)
Coordinates corresponding to the center of the main cluster of the *open* conformer (i.e., *principal open* conformer) (PDB)
Coordinates corresponding to the center of the main cluster of the *closed* conformer (i.e., *principal closed* conformer) (PDB)
Table S10: Backbone RMSD (Å) between experimentally resolved polymorphs of Aβ or IAPP and the Aβ and IAPP structures of the principal open and closed conformers; PDBs containing multiple conformers are presented intentionally (i.e., 8EZE models 1–6), as they were all considered for the comparison with the IAPP structures; the “*” and “**” denote that the PDBs corresponded to structures with two fibrils, with “*” corresponding to the fibril with the largest number of residues, and “**” corresponding to the fibril with the smallest number of residues; the table is symmetric with both rows and columns corresponding to the Aβ experimentally resolved polymorphs (yellow headings), IAPP experimentally resolved polymorphs (green headings) and the Aβ and IAPP structures of the open and closed heteromeric models after the conventional MD simulations (purple headings); each cell of the table contains the RMSD between the corresponding pair (given column and row); the RMSD values reported in the tables are symmetric with the diagonal having only zeroes (0.0) as it corresponds to the RMSD between a structure with itself; each pair of structures in the tables was superimposed in the region 18–42 for Aβ and 13–37 for IAPP, and RMSD calculations were performed in the same superimposed region; conditional formatting has been applied to the cells, with blue indicating lower RMSD values and red indicating higher RMSD values, to enhance readability; the selected experimentally resolved polymorphs compared with our models were selected based on criteria explained in the supporting methods (S.2.3) (XLSX)

## References

[1] Sawaya, M. R. ; Hughes, M. P. ; Rodriguez, J. A. ; Riek, R. ; Eisenberg, D. S. The expanding amyloid family: Structure, stability, function, and pathogenesis. *Cell.* 2021, *184* (19), 4857–4873, 10.1016/j.cell.2021.08.013 34534463 PMC8772536

[2] Jo, M. ; Lee, S. ; Jeon, Y. M. ; Kim, S. ; Kwon, Y. ; Kim, H. J. The role of TDP-43 propagation in neurodegenerative diseases: integrating insights from clinical and experimental studies. *Exp Mol. Med.* 2020, *52* (10), 1652–1662, 10.1038/s12276-020-00513-7 33051572 PMC8080625

[3] Holtzman, D. M. ; Morris, J. C. ; Goate, A. M. Alzheimer’s disease: the challenge of the second century. *Sci. Transl. Med.* 2011, *3* (77), 77sr1, 10.1126/scitranslmed.3002369 21471435 PMC3130546

[4] Jucker, M. ; Walker, L. C. Pathogenic protein seeding in Alzheimer disease and other neurodegenerative disorders. *Annals of neurology.* 2011, *70* (4), 532–540, 10.1002/ana.22615 22028219 PMC3203752

[5] Ivanova, M. I. ; Lin, Y. ; Lee, Y. H. ; Zheng, J. ; Ramamoorthy, A. Biophysical processes underlying cross-seeding in amyloid aggregation and implications in amyloid pathology. *Biophys Chem.* 2021, *269* , 106507 10.1016/j.bpc.2020.106507 33254009 PMC10317075

[6] Baba, M. ; Nakajo, S. ; Tu, P. H. ; Tomita, T. ; Nakaya, K. ; Lee, V. M. ; Trojanowski, J. Q. ; Iwatsubo, T. Aggregation of alpha-synuclein in Lewy bodies of sporadic Parkinson’s disease and dementia with Lewy bodies. *Am. J. Pathol.* 1998, *152* (4), 879 9546347 PMC1858234

[7] Johnson, K. H. ; O’brien, T. D. ; Betsholtz, C. ; Westermark, P. Islet amyloid polypeptide: mechanisms of amyloidogenesis in the pancreatic islets and potential roles in diabetes mellitus. *Lab. Investig.; J. Tech. Methods Pathol.* 1992, *66* (5), 522–535 1573849

[8] Maji, S. K. ; Perrin, M. H. ; Sawaya, M. R. ; Jessberger, S. ; Vadodaria, K. ; Rissman, R. A. ; Singru, P. S. ; Nilsson, K. P. ; Simon, R. ; Schubert, D. ; Eisenberg, D. Functional amyloids as natural storage of peptide hormones in pituitary secretory granules. *Science* 2009, *325* (5938), 328–332, 10.1126/science.1173155 19541956 PMC2865899

[9] Prusiner, S. B. Neurodegenerative diseases and prions. *New England Journal of Medicine.* 2001, *344* (20), 1516–1526, 10.1056/NEJM200105173442006 11357156

[10] Collinge, J. ; Clarke, A. R. A general model of prion strains and their pathogenicity. *Science.* 2007, *318* (5852), 930–936, 10.1126/science.1138718 17991853

[11] Goedert, M. Alzheimer’s and Parkinson’s diseases: The prion concept in relation to assembled Aβ, tau, and α-synuclein. *Science* 2015, *349* (6248), 1255555 10.1126/science.1255555 26250687

[12] Li, D. ; Liu, C. Molecular rules governing the structural polymorphism of amyloid fibrils in neurodegenerative diseases. *Structure.* 2023, *31* (11), 1335–1347, 10.1016/j.str.2023.08.006 37657437

[13] Chaudhuri, P. ; Prajapati, K. P. ; Anand, B. G. ; Dubey, K. ; Kar, K. Amyloid cross-seeding raises new dimensions to understanding of amyloidogenesis mechanism. *Ageing Res. Rev.* 2019, *56* , 100937 10.1016/j.arr.2019.100937 31430565

[14] Dubey, K. ; Anand, B. G. ; Temgire, M. K. ; Kar, K. Evidence of rapid coaggregation of globular proteins during amyloid formation. *Biochemistry.* 2014, *53* (51), 8001–8004, 10.1021/bi501333q 25494036

[15] Anand, B. G. ; Dubey, K. ; Shekhawat, D. S. ; Kar, K. Intrinsic property of phenylalanine to trigger protein aggregation and hemolysis has a direct relevance to phenylketonuria. *Sci. Rep.* 2017, *7* (1), 11146, 10.1038/s41598-017-10911-z 28894147 PMC5593866

[16] Anand, B. G. ; Prajapati, K. P. ; Kar, K. Aβ1–40 mediated aggregation of proteins and metabolites unveils the relevance of amyloid cross-seeding in amyloidogenesis. *Biochem. Biophys. Res. Commun.* 2018, *501* (1), 158–164, 10.1016/j.bbrc.2018.04.198 29723530

[17] Tavassoly, O. ; Sade, D. ; Bera, S. ; Shaham-Niv, S. ; Vocadlo, D. J. ; Gazit, E. Quinolinic acid amyloid-like fibrillar assemblies seed α-synuclein aggregation. *Journal of molecular biology.* 2018, *430* (20), 3847–3862, 10.1016/j.jmb.2018.08.002 30098337

[18] Sarell, C. J. ; Stockley, P. G. ; Radford, S. E. Assessing the causes and consequences of co-polymerization in amyloid formation. *Prion.* 2013, *7* (5), 359–368, 10.4161/pri.26415 24025483 PMC4134340

[19] Ono, K. ; Takahashi, R. ; Ikeda, T. ; Yamada, M. Cross-seeding effects of amyloid β-protein and α-synuclein. *Journal of neurochemistry.* 2012, *122* (5), 883–890, 10.1111/j.1471-4159.2012.07847.x 22734715

[20] O’Nuallain, B. ; Williams, A. D. ; Westermark, P. ; Wetzel, R. Seeding specificity in amyloid growth induced by heterologous fibrils. *J. Biol. Chem.* 2004, *279* (17), 17490–17499, 10.1074/jbc.M311300200 14752113

[21] Oskarsson, M. E. ; Paulsson, J. F. ; Schultz, S. W. ; Ingelsson, M. ; Westermark, P. ; Westermark, G. T. In vivo seeding and cross-seeding of localized amyloidosis: a molecular link between type 2 diabetes and Alzheimer disease. *American journal of pathology.* 2015, *185* (3), 834–846, 10.1016/j.ajpath.2014.11.016 25700985

[22] Masliah, E. ; Rockenstein, E. ; Veinbergs, I. ; Sagara, Y. ; Mallory, M. ; Hashimoto, M. ; Mucke, L. β-Amyloid peptides enhance α-synuclein accumulation and neuronal deficits in a transgenic mouse model linking Alzheimer’s disease and Parkinson’s disease. *Proceedings of the National Academy of Sciences.* 2001, *98* (21), 12245–12250, 10.1073/pnas.211412398 PMC59799 11572944

[23] Moreno-Gonzalez, I. ; Edwards, G., III ; Salvadores, N. ; Shahnawaz, M. ; Diaz-Espinoza, R. ; Soto, C. Molecular interaction between type 2 diabetes and Alzheimer’s disease through cross-seeding of protein misfolding. *Molecular psychiatry.* 2017, *22* (9), 1327–1334, 10.1038/mp.2016.230 28044060 PMC5495631

[24] Ren, B. ; Zhang, Y. ; Zhang, M. ; Liu, Y. ; Zhang, D. ; Gong, X. ; Feng, Z. ; Tang, J. ; Chang, Y. ; Zheng, J. Fundamentals of cross-seeding of amyloid proteins: an introduction. *J. Mater. Chem. B* 2019, *7* (46), 7267–7282, 10.1039/C9TB01871A 31647489

[25] Wright, C. F. ; Teichmann, S. A. ; Clarke, J. ; Dobson, C. M. The importance of sequence diversity in the aggregation and evolution of proteins. *Nature.* 2005, *438* (7069), 878–881, 10.1038/nature04195 16341018

[26] Zhang, M. ; Hu, R. ; Chen, H. ; Gong, X. ; Zhou, F. ; Zhang, L. ; Zheng, J. Polymorphic Associations and Structures of the Cross-Seeding of Aβ1–42 and hIAPP1–37 Polypeptides. *J. Chem. Inf Model.* 2015, *55* (8), 1628–1639, 10.1021/acs.jcim.5b00166 26173078

[27] Sade, D. ; Shaham-Niv, S. ; Arnon, Z. A. ; Tavassoly, O. ; Gazit, E. Seeding of proteins into amyloid structures by metabolite assemblies may clarify certain unexplained epidemiological associations. *Open Biol.* 2018, *8* (1), 170229 10.1098/rsob.170229 29367352 PMC5795054

[28] Gour, N. ; Gazit, E. Metabolite assemblies: A surprising extension to the amyloid hypothesis. *Curr. Opin Chem. Biol.* 2021, *64* , 154–164, 10.1016/j.cbpa.2021.07.005 34482124

[29] Subedi, S. ; Sasidharan, S. ; Nag, N. ; Saudagar, P. ; Tripathi, T. Amyloid Cross-Seeding: Mechanism, Implication, and Inhibition. *Molecules.* 2022, *27* (6), 1776, 10.3390/molecules27061776 35335141 PMC8955620

[30] Morales, R. ; Moreno-Gonzalez, I. ; Soto, C. Cross-seeding of misfolded proteins: implications for etiology and pathogenesis of protein misfolding diseases. *PLoS Pathog.* 2013, *9* (9), e1003537 10.1371/journal.ppat.1003537 24068917 PMC3777858

[31] Miklossy, J. ; Qing, H. ; Radenovic, A. ; Kis, A. ; Vileno, B. ; Làszló, F. ; Miller, L. ; Martins, R. N. ; Waeber, G. ; Mooser, V. ; Bosman, F. ; Khalili, K. ; Darbinian, N. ; McGeer, P. L. Beta amyloid and hyperphosphorylated tau deposits in the pancreas in type 2 diabetes. *Neurobiol Aging.* 2010, *31* (9), 1503–1515, 10.1016/j.neurobiolaging.2008.08.019 18950899 PMC4140193

[32] Ristow, M. Neurodegenerative disorders associated with diabetes mellitus. *J. Mol. Med. (Berl).* 2004, *82* (8), 510–529, 10.1007/s00109-004-0552-1 15175861

[33] Fawver, J. N. ; Ghiwot, Y. ; Koola, C. ; Carrera, W. ; Rodriguez-Rivera, J. ; Hernandez, C. ; Dineley, K. T. ; Kong, Y. ; Li, J. ; Jhamandas, J. ; Perry, G. ; Murray, I. V. Islet amyloid polypeptide (IAPP): a second amyloid in Alzheimer’s disease. *Curr. Alzheimer Res.* 2014, *11* (10), 928–940, 10.2174/1567205011666141107124538 25387341

[34] Banks, W. A. ; Kastin, A. J. ; Maness, L. M. ; Huang, W. ; Jaspan, J. B. Permeability of the blood-brain barrier to amylin. *Life Sci.* 1995, *57* (22), 1993–2001, 10.1016/0024-3205(95)02197-Q 7475950

[35] Ge, W. Y. ; Deng, X. ; Shi, W. P. ; Lin, W. J. ; Chen, L. L. ; Liang, H. ; Wang, X. T. ; Zhang, T. D. ; Zhao, F. Z. ; Guo, W. H. ; Yin, D. C. Amyloid Protein Cross-Seeding Provides a New Perspective on Multiple Diseases In Vivo. *Biomacromolecules.* 2023, *24* (1), 1–18, 10.1021/acs.biomac.2c01233 36507729

[36] Baghel, D. ; Ghosh, A. Heterotypic Interactions of Amyloid β and the Islet Amyloid Polypeptide Produce Mixed Aggregates with Non-Native Fibril Structure. *J. Phys. Chem. Lett.* 2024, *15* (49), 12197–12205, 10.1021/acs.jpclett.4c02827 39625456 PMC11781043

[37] Ly, H. ; Verma, N. ; Sharma, S. ; Kotiya, D. ; Despa, S. ; Abner, E. L. ; Nelson, P. T. ; Jicha, G. A. ; Wilcock, D. M. ; Goldstein, L. B. ; Guerreiro, R. ; Brás, J. ; Hanson, A. J. ; Craft, S. ; Murray, A. J. ; Biessels, G. J. ; Troakes, C. ; Zetterberg, H. ; Hardy, J. ; Lashley, T. ; AESG; Despa, F. The association of circulating amylin with β-amyloid in familial Alzheimer’s disease. *Alzheimers Dement.* 2021, *7* (1), e12130 10.1002/trc2.12130 PMC7816817 33521236

[38] Bharadwaj, P. ; Solomon, T. ; Sahoo, B. R. ; Ignasiak, K. ; Gaskin, S. ; Rowles, J. ; Verdile, G. ; Howard, M. J. ; Bond, C. S. ; Ramamoorthy, A. ; Martins, R. N. Amylin and beta amyloid proteins interact to form amorphous heterocomplexes with enhanced toxicity in neuronal cells. *Sci. Rep.* 2020, *10* (1), 10356, 10.1038/s41598-020-66602-9 32587390 PMC7316712

[39] Ono, K. ; Takahashi, R. ; Ikeda, T. ; Mizuguchi, M. ; Hamaguchi, T. ; Yamada, M. Exogenous amyloidogenic proteins function as seeds in amyloid β-protein aggregation. *Biochim. Biophys. Acta* 2014, *1842* (4), 646–653, 10.1016/j.bbadis.2014.01.002 24440525

[40] Krotee, P. ; Griner, S. L. ; Sawaya, M. R. ; Cascio, D. ; Rodriguez, J. A. ; Shi, D. ; Philipp, S. ; Murray, K. ; Saelices, L. ; Lee, J. ; Seidler, P. ; Glabe, C. G. ; Jiang, L. ; Gonen, T. ; Eisenberg, D. S. Common fibrillar spines of amyloid-β and human islet amyloid polypeptide revealed by microelectron diffraction and structure-based inhibitors. *J. Biol. Chem.* 2018, *293* (8), 2888–2902, 10.1074/jbc.M117.806109 29282295 PMC5827424

[41] Hu, R. ; Zhang, M. ; Chen, H. ; Jiang, B. ; Zheng, J. Cross-Seeding Interaction between β-Amyloid and Human Islet Amyloid Polypeptide. *ACS Chem. Neurosci.* 2015, *6* (10), 1759–1768, 10.1021/acschemneuro.5b00192 26255739

[42] Yan, L. M. ; Velkova, A. ; Tatarek-Nossol, M. ; Andreetto, E. ; Kapurniotu, A. IAPP mimic blocks Abeta cytotoxic self-assembly: cross-suppression of amyloid toxicity of Abeta and IAPP suggests a molecular link between Alzheimer’s disease and type II diabetes. *Angew. Chem., Int. Ed. Engl.* 2007, *46* (8), 1246–1252, 10.1002/anie.200604056 17203498

[43] Yan, L. M. ; Velkova, A. ; Kapurniotu, A. Molecular characterization of the hetero-assembly of β-amyloid peptide with islet amyloid polypeptide. *Curr. Pharm. Des.* 2014, *20* (8), 1182–1191, 10.2174/13816128113199990064 23713771

[44] Andreetto, E. ; Yan, L. M. ; Tatarek-Nossol, M. ; Velkova, A. ; Frank, R. ; Kapurniotu, A. Identification of hot regions of the Abeta-IAPP interaction interface as high-affinity binding sites in both cross- and self-association. *Angew. Chem., Int. Ed. Engl.* 2010, *49* (17), 3081–3085, 10.1002/anie.200904902 20309983

[45] Taş, K. ; Volta, B. D. ; Lindner, C. ; El Bounkari, O. ; Hille, K. ; Tian, Y. ; Puig-Bosch, X. ; Ballmann, M. ; Hornung, S. ; Ortner, M. ; Prem, S. ; Meier, L. ; Rammes, G. ; Haslbeck, M. ; Weber, C. ; Megens, R. T. A. ; Bernhagen, J. ; Kapurniotu, A. Designed peptides as nanomolar cross-amyloid inhibitors acting via supramolecular nanofiber co-assembly. *Nat. Commun.* 2022, *13* (1), 5004, 10.1038/s41467-022-32688-0 36008417 PMC9411207

[46] Zhang, Y. ; Tang, Y. ; Zhang, D. ; Liu, Y. ; He, J. ; Chang, Y. ; Zheng, J. Amyloid cross-seeding between Aβ and hIAPP in relation to the pathogenesis of Alzheimer and type 2 diabetes. *Chinese Journal of Chemical Engineering.* 2021, *30* , 225–235, 10.1016/j.cjche.2020.09.033

[47] D’Urso, L. ; Condorelli, M. ; Puglisi, O. ; Tempra, C. ; Lolicato, F. ; Compagnini, G. ; La Rosa, C. Detection and characterization at nM concentration of oligomers formed by hIAPP, Aβ (1–40) and their equimolar mixture using SERS and MD simulations. *Phys. Chem. Chem. Phys.* 2018, *20* (31), 20588–20596, 10.1039/C7CP08552D 30059089

[48] Seeliger, J. ; Weise, K. ; Opitz, N. ; Winter, R. The effect of Aβ on IAPP aggregation in the presence of an isolated β-cell membrane. *J. Mol. Biol.* 2012, *421* (2–3), 348–363, 10.1016/j.jmb.2012.01.048 22321797 PMC4075326

[49] Luo, J. ; Wärmländer, S. K. ; Gräslund, A. ; Abrahams, J. P. Cross-interactions between the Alzheimer Disease Amyloid-β Peptide and Other Amyloid Proteins: A Further Aspect of the Amyloid Cascade Hypothesis. *J. Biol. Chem.* 2016, *291* (32), 16485–16493, 10.1074/jbc.R116.714576 27325705 PMC4974365

[50] Baram, M. ; Atsmon-Raz, Y. ; Ma, B. ; Nussinov, R. ; Miller, Y. Amylin-Aβ oligomers at atomic resolution using molecular dynamics simulations: a link between Type 2 diabetes and Alzheimer’s disease. *Phys. Chem. Chem. Phys.* 2016, *18* (4), 2330–2338, 10.1039/C5CP03338A 26349542 PMC4720542

[51] Fan, X. ; Zhang, X. ; Yan, J. ; Xu, H. ; Zhao, W. ; Ding, F. ; Huang, F. ; Sun, Y. Computational Investigation of coaggregation and Cross-Seeding between Aβ and hIAPP Underpinning the Cross-Talk in Alzheimer’s Disease and Type 2 Diabetes. *J. Chem. Inf Model.* 2024, *64* (13), 5303–5316, 10.1021/acs.jcim.4c00859 38921060 PMC11339732

[52] Zhang, M. ; Hu, R. ; Ren, B. ; Chen, H. ; Jiang, B. ; Ma, J. ; Zheng, J. Molecular Understanding of Aβ-hIAPP Cross-Seeding Assemblies on Lipid Membranes. *ACS Chem. Neurosci.* 2017, *8* (3), 524–537, 10.1021/acschemneuro.6b00247 27936589

[53] Ge, X. ; Yang, Y. ; Sun, Y. ; Cao, W. ; Ding, F. Islet Amyloid Polypeptide Promotes Amyloid-Beta Aggregation by Binding-Induced Helix-Unfolding of the Amyloidogenic Core. *ACS Chem. Neurosci.* 2018, *9* (5), 967–975, 10.1021/acschemneuro.7b00396 29378116 PMC5955824

[54] Li, X. ; Lao, Z. ; Zou, Y. ; Dong, X. ; Li, L. ; Wei, G. Mechanistic Insights into the Co-Aggregation of Aβ and hIAPP: An All-Atom Molecular Dynamic Study. *J. Phys. Chem. B* 2021, *125* (8), 2050–2060, 10.1021/acs.jpcb.0c11132 33616398

[55] Song, Z. ; Tang, H. ; Gatch, A. ; Sun, Y. ; Ding, F. Islet amyloid polypeptide fibril catalyzes amyloid-β aggregation by promoting fibril nucleation rather than direct axial growth. *Int. J. Biol. Macromol.* 2024, *279* (Pt 1), 135137 10.1016/j.ijbiomac.2024.135137 39208885 PMC11469950

[56] Sun, Y. ; Wang, B. ; Ge, X. ; Ding, F. Distinct oligomerization and fibrillization dynamics of amyloid core sequences of amyloid-beta and islet amyloid polypeptide. *Phys. Chem. Chem. Phys.* 2017, *19* (41), 28414–28423, 10.1039/C7CP05695H 29038815 PMC5657190

[57] Kawecki, G. E. ; King, K. M. ; Cramer, N. A. ; Bevan, D. R. ; Brown, A. M. Simulations of cross-amyloid aggregation of amyloid-β and islet amyloid polypeptide fragments. *Biophys. J.* 2022, *121* (11), 2002–2013, 10.1016/j.bpj.2022.05.007 35538665 PMC9247468

[58] Berhanu, W. M. ; Yaşar, F. ; Hansmann, U. H. In silico cross seeding of Aβ and amylin fibril-like oligomers. *ACS Chem. Neurosci.* 2013, *4* (11), 1488–1500, 10.1021/cn400141x 24007594 PMC3837374

[59] Wojciechowski, J. W. ; Szczurek, W. ; Szulc, N. ; Szefczyk, M. ; Kotulska, M. PACT-Prediction of amyloid cross-interaction by threading. *Sci. Rep.* 2023, *13* (1), 22268, 10.1038/s41598-023-48886-9 38097650 PMC10721876

[60] Toyama, B. H. ; Weissman, J. S. Amyloid structure: conformational diversity and consequences. *Annu. Rev. Biochem.* 2011, *80* , 557–585, 10.1146/annurev-biochem-090908-120656 21456964 PMC3817101

[61] Riek, R. The Three-Dimensional Structures of Amyloids. *Cold Spring Harb Perspect Biol.* 2017, *9* (2), a023572 10.1101/cshperspect.a023572 27793967 PMC5287077

[62] Fändrich, M. ; Nyström, S. ; Nilsson, K. P. R. ; Böckmann, A. ; LeVine, H., 3rd ; Hammarström, P. Amyloid fibril polymorphism: a challenge for molecular imaging and therapy. *J. Intern Med.* 2018, *283* (3), 218–237, 10.1111/joim.12732 29360284 PMC5820168

[63] Tycko, R. Amyloid polymorphism: structural basis and neurobiological relevance. *Neuron.* 2015, *86* (3), 632–645, 10.1016/j.neuron.2015.03.017 25950632 PMC4425266

[64] Gremer, L. ; Schölzel, D. ; Schenk, C. ; Reinartz, E. ; Labahn, J. ; Ravelli, R. B. G. ; Tusche, M. ; Lopez-Iglesias, C. ; Hoyer, W. ; Heise, H. ; Willbold, D. ; Schröder, G. F. Fibril structure of amyloid-β(1–42) by cryo-electron microscopy. *Science.* 2017, *358* (6359), 116–119, 10.1126/science.aao2825 28882996 PMC6080689

[65] Cao, Q. ; Boyer, D. R. ; Sawaya, M. R. ; Abskharon, R. ; Saelices, L. ; Nguyen, B. A. ; Lu, J. ; Murray, K. A. ; Kandeel, F. ; Eisenberg, D. S. Cryo-EM structures of hIAPP fibrils seeded by patient-extracted fibrils reveal new polymorphs and conserved fibril cores. *Nat. Struct Mol. Biol.* 2021, *28* (9), 724–730, 10.1038/s41594-021-00646-x 34518699 PMC10396428

[66] Sievers, F. ; Wilm, A. ; Dineen, D. ; Gibson, T. J. ; Karplus, K. ; Li, W. ; Lopez, R. ; McWilliam, H. ; Remmert, M. ; Söding, J. ; Thompson, J. D. ; Higgins, D. G. Fast, scalable generation of high-quality protein multiple sequence alignments using Clustal Omega. *Mol. Syst. Biol.* 2011, *7* , 539, 10.1038/msb.2011.75 21988835 PMC3261699

[67] Nanga, R. P. ; Brender, J. R. ; Vivekanandan, S. ; Ramamoorthy, A. Structure and membrane orientation of IAPP in its natively amidated form at physiological pH in a membrane environment. *Biochim. Biophys. Acta* 2011, *1808* (10), 2337–2342, 10.1016/j.bbamem.2011.06.012 21723249 PMC3156962

[68] Humphrey, W. ; Dalke, A. ; Schulten, K. VMD: visual molecular dynamics. *J. Mol. Graph.* 1996, *14* (1), 33–38, 10.1016/0263-7855(96)00018-5 8744570

[69] Brooks, B. R. ; Brooks, C. L., 3rd ; Mackerell, A. D., Jr ; Nilsson, L. ; Petrella, R. J. ; Roux, B. ; Won, Y. ; Archontis, G. ; Bartels, C. ; Boresch, S. ; Caflisch, A. ; Caves, L. ; Cui, Q. ; Dinner, A. R. ; Feig, M. ; Fischer, S. ; Gao, J. ; Hodoscek, M. ; Im, W. ; Kuczera, K. ; Lazaridis, T. ; Ma, J. ; Ovchinnikov, V. ; Paci, E. ; Pastor, R. W. ; Post, C. B. ; Pu, J. Z. ; Schaefer, M. ; Tidor, B. ; Venable, R. M. ; Woodcock, H. L. ; Wu, X. ; Yang, W. ; York, D. M. ; Karplus, M. CHARMM: the biomolecular simulation program. *J. Comput. Chem.* 2009, *30* (10), 1545–1614, 10.1002/jcc.21287 19444816 PMC2810661

[70] Jo, S. ; Kim, T. ; Iyer, V. G. ; Im, W. CHARMM-GUI: a web-based graphical user interface for CHARMM. *J. Comput. Chem.* 2008, *29* (11), 1859–1865, 10.1002/jcc.20945 18351591

[71] Lee, J. ; Cheng, X. ; Swails, J. M. ; Yeom, M. S. ; Eastman, P. K. ; Lemkul, J. A. ; Wei, S. ; Buckner, J. ; Jeong, J. C. ; Qi, Y. ; Jo, S. ; Pande, V. S. ; Case, D. A. ; Brooks, C. L., 3rd ; MacKerell, A. D., Jr ; Klauda, J. B. ; Im, W. CHARMM-GUI Input Generator for NAMD, GROMACS, AMBER, OpenMM, and CHARMM/OpenMM Simulations Using the CHARMM36 Additive Force Field. *J. Chem. Theory Comput.* 2016, *12* (1), 405–413, 10.1021/acs.jctc.5b00935 26631602 PMC4712441

[72] Wang, K. W. ; Lee, J. ; Zhang, H. ; Suh, D. ; Im, W. CHARMM-GUI Implicit Solvent Modeler for Various Generalized Born Models in Different Simulation Programs. *J. Phys. Chem. B* 2022, *126* (38), 7354–7364, 10.1021/acs.jpcb.2c05294 36117287 PMC9551160

[73] Im, W. ; Lee, M. S. ; Brooks, C. L., 3rd Generalized born model with a simple smoothing function. *J. Comput. Chem.* 2003, *24* (14), 1691–1702, 10.1002/jcc.10321 12964188

[74] Seeber, M. ; Cecchini, M. ; Rao, F. ; Settanni, G. ; Caflisch, A. Wordom: a program for efficient analysis of molecular dynamics simulations. *Bioinformatics.* 2007, *23* (19), 2625–2627, 10.1093/bioinformatics/btm378 17717034

[75] Seeber, M. ; Felline, A. ; Raimondi, F. ; Muff, S. ; Friedman, R. ; Rao, F. ; Caflisch, A. ; Fanelli, F. Wordom: a user-friendly program for the analysis of molecular structures, trajectories, and free energy surfaces. *J. Comput. Chem.* 2011, *32* (6), 1183–1194, 10.1002/jcc.21688 21387345 PMC3151548

[76] Lee, M. S. ; Salsbury, F. R., Jr ; Brooks, C. L., III Novel generalized Born methods. *Journal of chemical physics.* 2002, *116* (24), 10606–10614, 10.1063/1.1480013

[77] Lee, M. S. ; Feig, M. ; Salsbury, F. R., Jr ; Brooks, C. L., 3rd New analytic approximation to the standard molecular volume definition and its application to generalized Born calculations. *J. Comput. Chem.* 2003, *24* (11), 1348–1356, 10.1002/jcc.10272 12827676

[78] Orr, A. A. ; Wördehoff, M. M. ; Hoyer, W. ; Tamamis, P. Uncovering the Binding and Specificity of β-Wrapins for Amyloid-β and α-Synuclein. *J. Phys. Chem. B* 2016, *120* (50), 12781–12794, 10.1021/acs.jpcb.6b08485 27934063

[79] Orr, A. A. ; Shaykhalishahi, H. ; Mirecka, E. A. ; Jonnalagadda, S. V. R. ; Hoyer, W. ; Tamamis, P. Elucidating the multi-targeted anti-amyloid activity and enhanced islet amyloid polypeptide binding of β-wrapins. *Comput. Chem. Eng.* 2018, *116* (116), 322–332, 10.1016/j.compchemeng.2018.02.013 30405276 PMC6217933

[80] Orr, A. A. ; Gonzalez-Rivera, J. C. ; Wilson, M. ; Bhikha, P. R. ; Wang, D. ; Contreras, L. M. ; Tamamis, P. A high-throughput and rapid computational method for screening of RNA post-transcriptional modifications that can be recognized by target proteins. *Methods.* 2018, *143* (143), 34–47, 10.1016/j.ymeth.2018.01.015 29408626

[81] Gonzalez-Rivera, J. C. ; Orr, A. A. ; Engels, S. M. ; Jakubowski, J. M. ; Sherman, M. W. ; O’Connor, K. N. ; Matteson, T. ; Woodcock, B. C. ; Contreras, L. M. ; Tamamis, P. Computational evolution of an RNA-binding protein towards enhanced oxidized-RNA binding. *Comput. Struct Biotechnol J.* 2020, *18* , 137–152, 10.1016/j.csbj.2019.12.003 31988703 PMC6965710

[82] Orr, A. A. ; Kuhlmann, S. K. ; Tamamis, P. Computational design of a β-wrapin’s N-terminal domain with canonical and non-canonical amino acid modifications mimicking curcumin’s proposed inhibitory function. *Biophys Chem.* 2022, *286* , 106805 10.1016/j.bpc.2022.106805 35417810

[83] Miller, L. G. ; Kim, W. ; Schowe, S. ; Taylor, K. ; Han, R. ; Jain, V. ; Park, R. ; Sherman, M. ; Fang, J. ; Ramirez, H. ; Ellington, A. ; Tamamis, P. ; Resendiz, M. J. E. ; Zhang, Y. J. ; Contreras, L. Selective 8-oxo-rG stalling occurs in the catalytic core of polynucleotide phosphorylase (PNPase) during degradation. *Proc. Natl. Acad. Sci. U. S. A.* 2024, *121* (46), e2317865121 10.1073/pnas.2317865121 39495922 PMC11572968

[84] Yang, Y. ; Arseni, D. ; Zhang, W. ; Huang, M. ; Lövestam, S. ; Schweighauser, M. ; Kotecha, A. ; Murzin, A. G. ; Peak-Chew, S. Y. ; Macdonald, J. ; Lavenir, I. ; Garringer, H. J. ; Gelpi, E. ; Newell, K. L. ; Kovacs, G. G. ; Vidal, R. ; Ghetti, B. ; Ryskeldi-Falcon, B. ; Scheres, S. H. W. ; Goedert, M. Cryo-EM structures of amyloid-β 42 filaments from human brains. *Science.* 2022, *375* (6577), 167–172, 10.1126/science.abm7285 35025654 PMC7612234

[85] Gallardo, R. ; Iadanza, M. G. ; Xu, Y. ; Heath, G. R. ; Foster, R. ; Radford, S. E. ; Ranson, N. A. Fibril structures of diabetes-related amylin variants reveal a basis for surface-templated assembly. *Nat. Struct Mol. Biol.* 2020, *27* (11), 1048–1056, 10.1038/s41594-020-0496-3 32929282 PMC7617688

[86] Xiao, Y. ; Ma, B. ; McElheny, D. ; Parthasarathy, S. ; Long, F. ; Hoshi, M. ; Nussinov, R. ; Ishii, Y. Aβ(1–42) fibril structure illuminates self-recognition and replication of amyloid in Alzheimer’s disease. *Nat. Struct Mol. Biol.* 2015, *22* (6), 499–505, 10.1038/nsmb.2991 25938662 PMC4476499

[87] Kalitnik, A. ; Lassota, A. ; Polańska, O. ; Gąsior-Głogowska, M. ; Szefczyk, M. ; Barbach, A. ; Chilimoniuk, J. ; Jęśkowiak-Kossakowska, I. ; Wojciechowska, A. W. ; Wojciechowski, J. W. ; Szulc, N. ; Kotulska, M. ; Burdukiewicz, M. Experimental methods for studying amyloid cross-interactions. *Protein Sci.* 2025, *34* (6), e70151 10.1002/pro.70151 40384558 PMC12086524

[88] Burdukiewicz, M. ; Rafacz, D. ; Barbach, A. ; Hubicka, K. ; Bąkała, L. ; Lassota, A. ; Stecko, J. ; Szymańska, N. ; Wojciechowski, J. W. ; Kozakiewicz, D. ; Szulc, N. ; Chilimoniuk, J. ; Jęśkowiak, I. ; Gąsior-Głogowska, M. ; Kotulska, M. AmyloGraph: a comprehensive database of amyloid-amyloid interactions. *Nucleic Acids Res.* 2023, *51* (D1), D352–D357, 10.1093/nar/gkac882 36243982 PMC9825533

[89] Bondarev, S. A. ; Uspenskaya, M. V. ; Leclercq, J. ; Falgarone, T. ; Zhouravleva, G. A. ; Kajava, A. V. AmyloComp: A Bioinformatic Tool for Prediction of Amyloid Co-aggregation. *J. Mol. Biol.* 2024, *436* (17), 168437 10.1016/j.jmb.2024.168437 38185324

[90] Kokotidou, C. ; Jonnalagadda, S. V. R. ; Orr, A. A. ; Vrentzos, G. ; Kretsovali, A. ; Tamamis, P. ; Mitraki, A. A. Designer Amyloid Cell-Penetrating Peptides for Potential Use as Gene Transfer Vehicles. *Biomolecules.* 2020, *10* (1), 7, 10.3390/biom10010007 PMC7023140 31861408

[91] Jonnalagadda, S. V. R. ; Kokotidou, C. ; Orr, A. A. ; Fotopoulou, E. ; Henderson, K. J. ; Choi, C. H. ; Lim, W. T. ; Choi, S. J. ; Jeong, H. K. ; Mitraki, A. ; Tamamis, P. Computational Design of Functional Amyloid Materials with Cesium Binding, Deposition, and Capture Properties. *J. Phys. Chem. B* 2018, *122* (30), 7555–7568, 10.1021/acs.jpcb.8b04103 29975835

[92] Kokotidou, C. ; Jonnalagadda, S. V. R. ; Orr, A. A. ; Seoane-Blanco, M. ; Apostolidou, C. P. ; van Raaij, M. J. ; Kotzabasaki, M. ; Chatzoudis, A. ; Jakubowski, J. M. ; Mossou, E. ; Forsyth, V. T. ; Mitchell, E. P. ; Bowler, M. W. ; Llamas-Saiz, A. L. ; Tamamis, P. ; Mitraki, A. A novel amyloid designable scaffold and potential inhibitor inspired by GAIIG of amyloid beta and the HIV-1 V3 loop. *FEBS Lett.* 2018, *592* (11), 1777–1788, 10.1002/1873-3468.13096 29772603

[93] Deidda, G. ; Jonnalagadda, S. V. R. ; Spies, J. W. ; Ranella, A. ; Mossou, E. ; Forsyth, V. T. ; Mitchell, E. P. ; Bowler, M. W. ; Tamamis, P. ; Mitraki, A. Self-Assembled Amyloid Peptides with Arg-Gly-Asp (RGD) Motifs As Scaffolds for Tissue Engineering. *ACS Biomater Sci. Eng.* 2017, *3* (7), 1404–1416, 10.1021/acsbiomaterials.6b00570 33429698

[94] Tamamis, P. ; Kasotakis, E. ; Archontis, G. ; Mitraki, A. Combination of theoretical and experimental approaches for the design and study of fibril-forming peptides. In *Protein Design: Methods and Applications* ; Humana Press: New York, NY, 2014; pp. 53–70. 10.1007/978-1-4939-1486-9_3 25213410

[95] Tamamis, P. ; Terzaki, K. ; Kassinopoulos, M. ; Mastrogiannis, L. ; Mossou, E. ; Forsyth, V. T. ; Mitchell, E. P. ; Mitraki, A. ; Archontis, G. Self-assembly of an aspartate-rich sequence from the adenovirus fiber shaft: insights from molecular dynamics simulations and experiments. *J. Phys. Chem. B* 2014, *118* (7), 1765–1774, 10.1021/jp409988n 24437637

[96] Tamamis, P. ; Kasotakis, E. ; Mitraki, A. ; Archontis, G. Amyloid-like self-assembly of peptide sequences from the adenovirus fiber shaft: insights from molecular dynamics simulations. *J. Phys. Chem. B* 2009, *113* (47), 15639–15647, 10.1021/jp9066718 19863125

[97] Vlachou, A. ; Kumar, V. B. ; Tiwari, O. S. ; Rencus-Lazar, S. ; Chen, Y. ; Ozguney, B. ; Gazit, E. ; Tamamis, P. Co-Assembly of Cancer Drugs with Cyclo-HH Peptides: Insights from Simulations and Experiments. *ACS Appl. Bio Mater.* 2024, *7* (4), 2309–2324, 10.1021/acsabm.3c01304 PMC11022239 38478987

[98] Kumar, V. B. ; Ozguney, B. ; Vlachou, A. ; Chen, Y. ; Gazit, E. ; Tamamis, P. Peptide Self-Assembled Nanocarriers for Cancer Drug Delivery. *J. Phys. Chem. B* 2023, *127* (9), 1857–1871, 10.1021/acs.jpcb.2c06751 36812392 PMC10848270

[99] Orr, A. A. ; Chen, Y. ; Gazit, E. ; Tamamis, P. Computational and Experimental Protocols to Study Cyclo-dihistidine Self- and Co-assembly: Minimalistic Bio-assemblies with Enhanced Fluorescence and Drug Encapsulation Properties. *Methods Mol. Biol.* 2022, *2405* , 179–203, 10.1007/978-1-0716-1855-4_10 35298815

[100] Chen, Y. ; Yang, Y. ; Orr, A. A. ; Makam, P. ; Redko, B. ; Haimov, E. ; Wang, Y. ; Shimon, L. J. W. ; Rencus-Lazar, S. ; Ju, M. ; Tamamis, P. ; Dong, H. ; Gazit, E. Self-Assembled Peptide Nano-Superstructure towards Enzyme Mimicking Hydrolysis. *Angew. Chem., Int. Ed. Engl.* 2021, *60* (31), 17164–17170, 10.1002/anie.202105830 34014019

[101] Tao, K. ; Chen, Y. ; Orr, A. A. ; Tian, Z. ; Makam, P. ; Gilead, S. ; Si, M. ; Rencus-Lazar, S. ; Qu, S. ; Zhang, M. ; Tamamis, P. ; Gazit, E. Enhanced Fluorescence for Bioassembly by Environment-Switching Doping of Metal Ions. *Adv. Funct Mater.* 2020, *30* (10), 1909614, 10.1002/adfm.201909614 32256278 PMC7136075

[102] Chen, Y. ; Orr, A. A. ; Tao, K. ; Wang, Z. ; Ruggiero, A. ; Shimon, L. J. W. ; Schnaider, L. ; Goodall, A. ; Rencus-Lazar, S. ; Gilead, S. ; Slutsky, I. ; Tamamis, P. ; Tan, Z. ; Gazit, E. High-Efficiency Fluorescence through Bioinspired Supramolecular Self-Assembly. *ACS Nano* 2020, *14* (3), 2798–2807, 10.1021/acsnano.9b10024 32013408 PMC7098056

[103] Tamamis, P. ; Adler-Abramovich, L. ; Reches, M. ; Marshall, K. ; Sikorski, P. ; Serpell, L. ; Gazit, E. ; Archontis, G. Self-assembly of phenylalanine oligopeptides: insights from experiments and simulations. *Biophys. J.* 2009, *96* (12), 5020–5029, 10.1016/j.bpj.2009.03.026 19527662 PMC2712050

[104] Dupuis, N. F. ; Wu, C. ; Shea, J. E. ; Bowers, M. T. The amyloid formation mechanism in human IAPP: dimers have β-strand monomer-monomer interfaces. *J. Am. Chem. Soc.* 2011, *133* (19), 7240–7243, 10.1021/ja1081537 21517093 PMC3093713

[105] Divanach, P. ; Noti, A. ; Vouvopoulos, P. ; Athanasiou, T. ; Kountourakis, N. ; Harmandaris, V. ; Rissanou, A. N. ; Mitraki, A. FmocFF Peptide Hydrogel Is a Promising Matrix for Encapsulation and Controlled Release of the Anticancer Peptide Drug Bortezomib. *Biomolecules.* 2025, *15* (6), 839, 10.3390/biom15060839 40563480 PMC12191350

[106] Divanach, P. ; Fanouraki, E. ; Mitraki, A. ; Harmandaris, V. ; Rissanou, A. N. Self-Assembly of Phenylalanine-Leucine, Leucine-Phenylalanine, and Cyclo (-leucine-phenylalanine) Dipeptides through Simulations and Experiments. *J. Phys. Chem. B* 2023, *127* (19), 4208–4219, 10.1021/acs.jpcb.2c08576 37148280

[107] Kalapurakal, R. A. M. ; Rocha, B. C. ; Vashisth, H. Self-Assembly in an Experimentally Realistic Model of Lobed Patchy Colloids. *ACS Appl. Bio Mater.* 2024, *7* (2), 535–542, 10.1021/acsabm.2c00910 PMC10880053 36698242

[108] Sudarshan, T. R. ; Lim, S. ; Li, J. ; Robang, A. S. ; Liberty, L. M. ; Ardoña, H. A. M. ; Paravastu, A. K. Cooperative β-sheet coassembly controls intermolecular orientation of amphiphilic peptide-polydiacetylene conjugates. *Solid State Nucl. Magn. Reson.* 2024, *133* , 101959 10.1016/j.ssnmr.2024.101959 39213800

[109] Wong, K. M. ; Robang, A. S. ; Lint, A. H. ; Wang, Y. ; Dong, X. ; Xiao, X. ; Seroski, D. T. ; Liu, R. ; Shao, Q. ; Hudalla, G. A. ; Hall, C. K. ; Paravastu, A. K. Engineering β-Sheet Peptide Coassemblies for Biomaterial Applications. *J. Phys. Chem. B* 2021, *125* (50), 13599–13609, 10.1021/acs.jpcb.1c04873 34905370

[110] Watanabe-Nakayama, T. ; Nawa, M. ; Konno, H. ; Kodera, N. ; Ando, T. ; Teplow, D. B. ; Ono, K. Self- and Cross-Seeding on α-Synuclein Fibril Growth Kinetics and Structure Observed by High-Speed Atomic Force Microscopy. *ACS Nano* 2020, *14* (8), 9979–9989, 10.1021/acsnano.0c03074 32678577

[111] Rahimi Araghi, L. ; Dee, D. R. Cross-Species and Cross-Polymorph Seeding of Lysozyme Amyloid Reveals a Dominant Polymorph. *Front Mol. Biosci.* 2020, *7* , 206, 10.3389/fmolb.2020.00206 32923456 PMC7456942

[112] Padilla-Godínez, F. J. ; Vázquez-García, E. R. ; Trujillo-Villagrán, M. I. ; Soto-Rojas, L. O. ; Palomero-Rivero, M. ; Hernández-González, O. ; Pérez-Eugenio, F. ; Collazo-Navarrete, O. ; Arias-Carrión, O. ; Guerra-Crespo, M. α-synuclein and tau: interactions, cross-seeding, and the redefinition of synucleinopathies as complex proteinopathies. *Front Neurosci.* 2025, *19* (19), 1570553, 10.3389/fnins.2025.1570553 40212715 PMC11983482

[113] Jensen, P. H. ; Hager, H. ; Nielsen, M. S. ; Hojrup, P. ; Gliemann, J. ; Jakes, R. alpha-synuclein binds to Tau and stimulates the protein kinase A-catalyzed tau phosphorylation of serine residues 262 and 356. *J. Biol. Chem.* 1999, *274* (36), 25481–25489, 10.1074/jbc.274.36.25481 10464279

[114] Guo, J. L. ; Lee, V. M. Cell-to-cell transmission of pathogenic proteins in neurodegenerative diseases. *Nat. Med.* 2014, *20* (2), 130–138, 10.1038/nm.3457 24504409 PMC4011661

[115] Lu, J. ; Zhang, S. ; Ma, X. ; Jia, C. ; Liu, Z. ; Huang, C. ; Liu, C. ; Li, D. Structural basis of the interplay between α-synuclein and Tau in regulating pathological amyloid aggregation. *J. Biol. Chem.* 2020, *295* (21), 7470–7480, 10.1074/jbc.RA119.012284 32291284 PMC7247300

[116] Castillo-Carranza, D. L. ; Guerrero-Muñoz, M. J. ; Sengupta, U. ; Gerson, J. E. ; Kayed, R. α-Synuclein Oligomers Induce a Unique Toxic Tau Strain. *Biol. Psychiatry* 2018, *84* (7), 499–508, 10.1016/j.biopsych.2017.12.018 29478699 PMC6201292

[117] Hojjatian, A. ; Dasari, A. K. R. ; Sengupta, U. ; Taylor, D. ; Daneshparvar, N. ; Yeganeh, F. A. ; Dillard, L. ; Michael, B. ; Griffin, R. G. ; Borgnia, M. J. ; Kayed, R. ; Taylor, K. A. ; Lim, K. H. Tau induces formation of α-synuclein filaments with distinct molecular conformations. *Biochem. Biophys. Res. Commun.* 2021, *554* (554), 145–150, 10.1016/j.bbrc.2021.03.091 33798940 PMC8062303

[118] Dhakal, S. ; Wyant, C. E. ; George, H. E. ; Morgan, S. E. ; Rangachari, V. Prion-like C-Terminal Domain of TDP-43 and α-Synuclein Interact Synergistically to Generate Neurotoxic Hybrid Fibrils. *J. Mol. Biol.* 2021, *433* (10), 166953 10.1016/j.jmb.2021.166953 33771571 PMC8085152

[119] Vaneyck, J. ; Segers-Nolten, I. ; Broersen, K. ; Claessens, M. M. A. E. Cross-seeding of alpha-synuclein aggregation by amyloid fibrils of food proteins. *J. Biol. Chem.* 2021, *296* , 100358 10.1016/j.jbc.2021.100358 33539920 PMC7949133

