# Supplementary material for: Amyloid Cross-Interactions through the Lens of Simulations: The Case of Aβ–IAPP
Source: J Phys Chem B. 2025 Oct 6;129(41):10721–38. doi: 10.1021/acs.jpcb.5c04536 (PMC12536406; doi:10.1021/acs.jpcb.5c04536)
Supplement: Supplementary file 1 [file jp5c04536_si_001.pdf]

Amyloid Cross-Interactions through the Lens of Simulations: The case of Aβ-IAPP

Xenophon Xenophontos [a], Anastasia Vlachou [a], Ryleigh K. Hunt [a], Phanourios Tamamis [a,b,*]

[a] Artie McFerrin Department of Chemical Engineering, College of Engineering, Texas A&M University, College Station, TX 77843, USA

[b] Department of Materials Science and Engineering, College of Engineering, Texas A&M University, College Station, TX 77843, USA

* Corresponding author: tamamis@tamu.edu

Supporting Information

Table of Contents

List of Figures 3
List of Tables 4
S.2. Supporting Methods 5
S.2.1. Selection of the Initial Templates 5
S.2.2. Biased MD Simulations – Modeling the Heteromeric Cross-Interaction 7
S.2.3. Conventional MD Simulations and Analysis 8
S.3. Supporting Results 12
Supporting References 49

List of Figures

Figure S1: Sequence alignment of Aβ and IAPP produced using Clustal Omega, with high gap penalty. 5
Figure S2: Panels A-B, C-D, E-F, and G-H correspond to the backbone RMSD of chains D-E (junction), C-F (junction + 1 chain beyond), B-G (junction + 2 chains beyond – all chains but the exterior), and A-H (all chains), respectively, with respect to its initial structure (blue) and average structure (orange), as a function of time. Panels on the left correspond to the open heteromeric conformer, while panels on the right correspond to the closed heteromeric conformer. 12
Figure S3: Panels A and B show the number of backbone-backbone hydrogen bonds in the junction of the open (blue) and closed (orange) heteromeric conformers during the biased and conventional MD simulations, respectively. 16
Figure S4: The first alignment corresponds to the sequence alignment that we enforced during the biased MD simulations. The second and third alignment correspond to the alignment according to side chain complementarity and β-sheet hydrogen bonds after the conventional MD simulations for the open and closed conformers, respectively. 17
Figure S5: Panels A-B, C-D, E-F, and G-H show contact maps, backbone-backbone hydrogen-bond maps, non-polar interaction free energy maps, and polar interaction free energy maps, respectively, between residues of chains B and C of homomeric Aβ within the two heteromeric conformers. Panels on the left correspond to the open heteromeric conformer, while the panels on the right correspond to the closed heteromeric conformer. The values in panels A-D correspond to percentage probabilities/occupancies, while the values in panels E-H correspond to interaction free energies in kcal/mol. 19
Figure S6: Panels A-B, C-D, E-F, and G-H show contact maps, backbone-backbone hydrogen-bond maps, non-polar interaction free energy maps, and polar interaction free energy maps, respectively, between residues of chains C and D of homomeric Aβ within the two heteromeric conformers. Panels on the left correspond to the open heteromeric conformer, while the panels on the right correspond to the closed heteromeric conformer. The values in panels A-D correspond to percentage probabilities/occupancies, while the values in panels E-H correspond to interaction free energies in kcal/mol. 21
Figure S7: Panels A-B, C-D, E-F, and G-H show contact maps, backbone-backbone hydrogen-bond maps, non-polar interaction free energy maps, and polar interaction free energy maps, respectively, between residues of chains E and F of homomeric IAPP within the two heteromeric conformers. Panels on the left correspond to the open heteromeric conformer, while the panels on the right correspond to the closed heteromeric conformer. The values in panels A-D correspond to percentage probabilities/occupancies, while the values in panels E-H correspond to interaction free energies in kcal/mol. 23
Figure S8: Panels A-B, C-D, E-F, and G-H show contact maps, backbone-backbone hydrogen-bond maps, non-polar interaction free energy maps, and polar interaction free energy maps, respectively, between residues of chains F and G of homomeric IAPP within the two heteromeric conformers. Panels on the left correspond to the open heteromeric conformer, while the panels on the right correspond to the closed heteromeric conformer. The values in panels A-D correspond to percentage probabilities/occupancies, while the values in panels E-H correspond to interaction free energies in kcal/mol. 25

List of Tables

Table S1: Backbone RMSD (Å) calculated for all pairs between Aβ and IAPP experimentally resolved structures........ 5
Table S2: Summary of the variations of the "shape-like similarity" constraints that were used. 7
Table S3: Summary of results from 18 trajectories, on the β-sheet content and the state of Aβ N-terminal........ 9
Table S4A: Backbone RMSD (Å) between the initial structure of Aβ (5OQV), and the Aβ structures of the heteromeric conformers at the end of the biased MD simulations resulting in the open and closed conformers, as well as the principal open and closed conformers from the conventional MD simulations........ 13
Table S4B: Backbone RMSD (Å) between the initial (refined) structure of IAPP, and the IAPP structures of the heteromeric conformers at the end of the biased MD simulations resulting in the open and closed conformers, as well as the principal open and closed conformers from the conventional MD simulations........ 13
Table S5A: Backbone RMSD (Å) between the Aβ structures of the heteromeric conformers at the end of the biased MD Simulations resulting in the open and closed conformers, and the corresponding principal open and closed conformers from the conventional MD simulations. 14
Table S5B: Backbone RMSD (Å) between the IAPP structures of the heteromeric conformers, at the end of the biased MD Simulations resulting in the open and closed conformers, and the corresponding principal open and closed conformers from the conventional MD simulations. 14
Table S6A: Backbone RMSD (Å) between the Aβ structures of the principal open and closed conformers from the conventional MD simulations. 15
Table S6B: Backbone RMSD (Å) between the IAPP structures of the principal open and closed conformers from the conventional MD simulations. 15
Table S7A: The key interactions taking place in the junction of the open heteromeric conformer. 26
Table 7B: The key interactions taking place in the junction of the closed heteromeric conformer. 35
Table S8: Side chain-side chain or backbone-side chain hydrogen bonds within neighboring chains of both the homomeric regions (excluding exterior peptides) and the junction of open and closed heteromeric conformers. 44
Table S9A: Backbone RMSD (Å) between all pairs of chains within the *principal open* conformer. 47
Table S9B: Backbone RMSD (Å) between all pairs of chains within the *principal closed* conformer. 47
Table S9C: Backbone RMSD (Å) between all pairs of chains within the *principal open and closed* conformers. 47
Table S10: Backbone RMSD (Å) between experimentally resolved polymorphs of Aβ or IAPP and the Aβ and IAPP structures within the *principal open* and *closed* conformers. 48

S.2. Supporting Methods

S.2.1. Selection of the Initial Templates

Table S1: Backbone RMSD (Å) calculated for all pairs between Aβ and IAPP experimentally resolved structures.

	**5OQV[1]**	**8EZE[2] #1**	**8EZE[2] #2**	**8EZE[2] #3**	**8EZE[2] #4**	**8EZE[2] #5**	**8EZE[2] #6**
**7M62[3]**	18.1	13.6	13.3	13.7	13.7	13.7	13.5
**7M65*[3]**	8.4	13.1	13.1	13.1	13.0	13.1	13.0
**7YKW*[4]**	17.3	11.1	11.0	10.9	11.1	11.3	11.1
**7YL0*[4]**	19.8	14.8	14.8	14.7	14.9	15.1	14.9
**7YL3*[4]**	19.2	14.6	14.6	14.5	14.7	14.9	14.7
**7YL7*[4]**	17.2	10.2	10.1	10.	10.2	10.5	10.3

The table shows the PDB IDs corresponding to IAPP experimentally resolved conformers (rows) and Aβ experimentally resolved conformers (columns). The structures shown in the table were chosen based on the first evaluation criterion, structures with all the residues resolved for Aβ, and structures with at least 6-37 resolved for IAPP. PDBs containing multiple conformers are presented intentionally (8EZE[2] models 1-6), as they were all considered for the comparison with the IAPP structures. The "*" denotes that the PDBs corresponded to structures with two or more fibrils and only the fibril with the maximum number of residues was considered. For all the cases of structures whose PDB entries did not contain tetramers, a tetramer was modelled using VMD[5], based on the extracted trimers. For each pair, the entire resolved backbone conformations were superimposed, using VMD[5], such that the 11-38 region of Aβ was sequentially aligned with the 6-33 region of IAPP without considering any gaps before or after these regions, and the RMSD calculations were performed based on the aligned regions. Specifically, for the pairs of 7M62[3] with any Aβ structure or 7M65*[3] with any Aβ structure the region of superposition was 11-42 for Aβ and 6-37 for IAPP. For any other pair the region for superposition was 6-42 for Aβ and 1-37 for IAPP.

```
IAPP        -----KCNTATCATQRLANFLVHSSNNFGAILSSTNVGSNTY    37
Aβ          DAEFRHDSGYEVHHQKLVFFAEDVGSNKGAIIGLMVGGVVIA    42
```

Figure S1: Sequence alignment of Aβ and IAPP produced using Clustal Omega[6], with high gap penalty.

We exploited the multiple NMR structures (20 models) of IAPP by Nanga et al. (PDB ID: 2L86[7]) to select the best conformer based on which we would model the missing residues (1-5) of IAPP. First, we superimposed the backbone of residues 6-7 of all 20 models of 2L86[7] to residues 6-7 of 7M65[3] and we discarded all 2L86[7] models with clashes based on visual inspection in VMD[5]. Subsequently we calculated the RMSD between the superimposed regions (corresponding to the backbone of residues 6-7) of the remaining 2L86[7] models and 7M65[3] , and selected model #5 of 2L86[7], which corresponded to the lowest RMSD. Subsequently, residues 1-5, extracted from 2L86[7] model #5, were structurally appended to each chain of the IAPP tetramer extracted from 7M65[3] (residues 6–37) using VMD[5], resulting in an IAPP tetramer with complete chains/monomers (residues 1–37). Subsequently, we performed an energy minimization to improve the integrity of the modeled IAPP tetramer. We used CHARMM-GUI PDB Reader and Manipulator[8,9,10,11,12,13] to produce input files, where disulfide bridges between cysteine residues at positions 2 and 7 of each chain were introduced. We modified the generated input files to include 200 steps of energy minimization (100 steps of both steepest descent – SD and adopted basis Newton-Raphson ABNR) after we first fixed all residues except 1–7. The complete IAPP structure after the energy minimization was used as the initial IAPP structure for the modeling of the heteromeric cross-interaction and the IAPP control runs.

S.2.2. Biased MD Simulations – Modeling the Heteromeric Cross-Interaction

The first set of constraints comprised constraints between corresponding Cα:Cα atom pairs of neighboring chains, both identical (i.e., homomeric) and within the junction (i.e., heteromeric). These constraints are referred to as "β-sheet-like" constraints, as they facilitate the formation of hydrogen bonding interactions within β-sheets. Within the junction, β-sheet-like constraints were introduced between corresponding Cα:Cα atom pairs of the 9-42 Aβ region and 4-37 IAPP region, as shown in Figure 1B and Figure S1.

These constraints consisted of Miscellaneous Mean Field Potential (MMFP) relative distance constraints of 5 Å between corresponding Cα:Cα atom pairs of neighboring chains (e.g., for homomeric chains, between the Cα of residue i in chain A and the Cα of residue i of chain B, for i = 1, 2, 3, ... e.g., for the junction, between the Cα of residue i in chain D and the Cα of residue j of chain E, for i = 9, 10, …,42 and j = 4, 5, ..., 37). These constraints employed a symmetric harmonic force between the atom pairs with a CHARMM force constant of 2 kcal/mol $Å^2$.

The second set of constraints comprised six variations of shape-like similarity constraints, which were all applied independently, and aimed to facilitate shape similarity across homomeric chains, with different sets of CHARMM[9] force constants (shown below in Table S2), variably reduced as approaching the junction, as shown in Figure 1B. These constraints consisted of harmonic relative mass constraints between corresponding C-C or N-N backbone atom pairs of neighboring chains (e.g., between C atom of residue i in chain A and C atom of residue i in chain B & between N atom of residue i in chain B and N atom of residue i in chain C, for i = 1, 2, 3, ...).

Table S2: Summary of the variations of the "shape-like similarity" constraints that were used.

chain pairs	CHARMM-Force Constant (kcal/mol/ $Å^2$)					
	Variant 1: ●	Variant 2: ✕	Variant 3: ■	Variant 4: ◆	Variant 5: ★	Variant 6: ✚
A-B	10.0	3.00	3.00	3.00	1.00	1.00
B-C	1.00	3.00	3.00	3.00	1.00	1.00
C-D	0.10	0.10	0.00	0.30	0.10	0.00
D-E	0.00	0.00	0.00	0.30	0.00	0.00
E-F	0.10	0.10	0.00	0.30	0.10	0.00
F-G	1.00	3.00	3.00	3.00	1.00	1.00
G-H	10.0	3.00	3.00	3.00	1.00	1.00

The first column corresponds to the chain pair, while the rest of the columns are showing the value of the CHARMM[9] force constant of each variant corresponding to each chain pair. The symbols shown under each variant are the symbols that were used to demonstrate the shape-like similarity constraints in Figure 1B.

We performed short, biased MD simulations, concurrently combining the first set and second set (comprising six variations, independently) of constraints. Prior to MD simulations, the two tetramer modeled fibrils of Aβ and IAPP were initially brought in proximity, such that the

sequentially similar 28-34 residue moiety of Aβ and 23-29 of IAPP were in proximity (with an average distance between corresponding Cα:Cα atom pairs of ~5.1 Å), and an energy minimization (200 steps of steepest descent and 200 steps of ABNR) was used to avoid any steric clashes. Subsequently, MD simulations were introduced. CHARMM-GUI Implicit Solvent Generator module[8,9,10,14] was used to generate the input files, which we modified to introduce the constraints described above. The biased MD Simulations were conducted at pH 7 and 300 K for 2 ns (1,000,000 steps with a timestep of 0.002 ps) using Langevin Dynamics. We adopted the Generalized Born with a Smooth Switching (GBSW) Implicit Solvent Model[15] applying a surface tension coefficient (sgamma) of 0.005 kcal/mol $Å^2$, 50 angular grid points and an ionic concentration of 0.15 M. Disulfide bridge patches were introduced between cysteine residues at positions 2 and 7 of all chains of IAPP, along with amidated C-terminal patches (CT2).

S.2.3. Conventional MD Simulations and Analysis

For the setup of the simulated systems and the generation of the input files for equilibration and production simulations we used CHARMM-GUI Solution Builder[8,9,10]. Triplicate simulations were performed at pH 7 with each run starting from the same structure but with different initial velocities to ensure statistical independence. Disulfide bridge patches were introduced between cysteine residues at positions 2 and 7 of all chains of IAPP, along with amidated C-terminal patches (CT2). For each system, the modeled fibril was centered in a cubic periodic boundary condition (PBC) box, with a minimum distance of 20 Å between any fibril atom and the box edges. The systems were solvated with explicit water molecules, and sodium and chloride ions were added to reach a physiological ionic concentration of 0.15 M. Simulations were performed using OpenMM[16]. Each system underwent 1 ns of equilibration (NVT) at 300 K, with a timestep of 0.001 ps, during which harmonic constraints were applied on heavy backbone atoms with a force constant of 400 kJ/mol·nm^2 and heavy side chain atoms with a force constant of 40 kJ/mol·nm^2. This was followed by 100 ns of production (NPT) at 300 K and 1 atm, with a timestep of 0.002 ps, during which no constraints were applied.

Evaluation of the β-sheet content for all 18 simulation trajectories after the end of the 100 ns conventional MD simulations was conducted. To assess the β-sheet content of each trajectory we calculated the average number of residues found in β-sheet configuration in the last 25 ns of each trajectory. To calculate the average number of residues in β-sheet configuration, we summed all residues found in β-sheet configuration in each snapshot/conformation in the last 25 ns of each trajectory and we divided with the number of snapshots/conformations analyzed. The calculations were performed using STRIDE[17], via VMD[5], considering all residues in extended configuration (E) or isolated β-bridge (B) configuration. The calculations were performed after we isolated only the chains of the junction and one chain beyond (i.e., chains C, D, E, and F) for each system, while the rest were deleted using VMD[5]. Similarly, calculations were also performed after we isolated only the chains of the junction and two chains beyond (i.e., chains B, C, D, E, F, and G) for each system, while the rest were deleted using VMD[5]. The results of this analysis are shown in Table S3. Subsequently, the two trajectories corresponding to the highest degree of β-sheet content were selected for extended conventional MD simulations and further analysis.

Table S3: Summary of results from 18 trajectories, on the β-sheet content and the state of Aβ N-terminal.

Originating from different variants of "shape-like" similarity	Number of run	Average number of residues in β-sheet for chains C-F	Average number of residues in β-sheet for chains B-G	Description
Variant 1	1	99.68	146.40	Open
Variant 1	2	102.64	154.00	Open
Variant 1	3	99.48	149.20	Open
Variant 2	1	93.52	143.24	Open
Variant 2	2	91.64	140.36	Closed
Variant 2	3	86.08	135.88	Open
Variant 3	1	103.08	154.96	Closed
Variant 3	2	78.52	126.12	Open
Variant 3	3	93.36	140.16	Closed
Variant 4	1	86.88	134.56	Closed
Variant 4	2	87.84	134.96	Closed
Variant 4	3	94.52	143.08	Closed
Variant 5	1	87.64	143.20	Open
Variant 5	2	99.16	148.24	Open
Variant 5	3	88.76	143.00	Open
Variant 6	1	88.72	137.84	Open
Variant 6	2	94.60	142.96	Open
Variant 6	3	97.48	149.40	Open

Results from the secondary structure analysis performed after the conclusion of the 100 ns conventional MD simulations. The first column corresponds to the variant (1-6) of the shape-like similarity constraints used to produce the structure based on which the conventional MD simulations were performed. The second column corresponds to the number of replicate (1-3) of the structure. The first two columns are used as a means to distinguish between the 18 runs of simulations. The third column corresponds to the average number of residues found in β-sheet configuration calculated using the last 25 ns of the trajectory of each run. The calculations were performed only considering the chains of the junction and one chain beyond (i.e., chains C, D, E, and F) for each system, by isolating them while the rest were deleted. The calculations were done using STRIDE[17], via VMD[5], considering all residues in extended configuration (E) or isolated β-bridge (B) configuration. Similarly with the third column, the fourth column corresponds to the average number of residues found in β-sheet configuration, with the difference that these calculations were performed only considering the chains of the junction and two chains beyond (i.e., chains B, C, D, E, F, and G). The fifth column corresponds to a description referring to the Aβ peptides' N-terminal. "Open" refers to an open/exposed N-terminal of Aβ, while "Closed" refers to a closed/contracted N-terminal of Aβ. The description was based on visual inspection of the last simulation snapshot of each run. The two runs with the highest degree of β-sheet content are shown highlighted in yellow. These trajectories were extended to 500 ns and energetic and structural analysis was conducted.

Using the simulation snapshots within the main clusters from the heteromeric conformers we calculated the backbone-backbone hydrogen bond occupancies between atom pairs of neighboring chains, using the VMD[5] Hydrogen Bond tool, with a cutoff distance of 3.5 Å and cutoff angle of 90°. Maps were generated to illustrate the backbone-backbone hydrogen bond occupancies (%). Additionally, using the same tool and cutoffs, we calculated the backbone-side chain or the side chain-side chain hydrogen bond occupancies between atom pairs of neighboring chains. Furthermore, using the simulation snapshots within the main clusters from the heteromeric conformers we calculated the contact probabilities between residue pairs of neighboring chains, using in-house FORTRAN scripts. To calculate the contact probabilities we counted the total number of instances that two residues were in contact and divided it by the total number of simulation snapshots that were analyzed. Two residues were considered to be in contact when any of their atoms (including hydrogens) were in proximity of ≤ 3.5 Å. Maps were generated to illustrate the residue-pairwise contact probabilities (%).

Moreover, we calculated the interaction free energy between residue pairs of neighboring chains, using in-house CHARMM[9] scripts employing the GBMV[18,19] implicit solvent model method II, as provided by CHARMM-GUI[8,9,10,14]. The setup of calculations followed previous studies in our lab [20,21,22,23,24,25], and the interaction free energy of the residue pairs, was decomposed into polar and non-polar contributions. Maps were generated to illustrate the residue-pairwise interaction free energy decomposed into polar and non-polar contributions. The residue-pairwise interaction free energy values were summed up for every residue, considering its interactions with other residues in neighboring chains. Subsequently, we summed-up all interaction free energy values of every residue per chain, and then divided by the total number of residues per chain, to compare evenly between Aβ and IAPP peptides in the *open* and *closed* heteromeric conformers. This residue-normalized interaction free energy aimed to evenly compare interaction energies per chain, in an "independent of molecule size"[26] manner. Histograms were generated to illustrate residue-normalized interaction free energy of each chain. The same calculations for interaction free energy and residue-normalized interaction free energy were conducted for the control systems (homomeric Aβ and homomeric IAPP) to be compared with the heteromeric conformers. The calculations were performed every 18 ns interval using the simulation snapshots within the main clusters from the heteromeric conformers. For the control runs, the average residue-normalized interaction free energy of each chain was calculated from the triplicate runs.

We compared the structures of Aβ and IAPP, individually, of the *principal open* and *closed conformers* to the structures of the biased MD simulations they originated from (2.2), as well as their initial/refined conformations (2.1). The backbone RMSD was calculated between the aforementioned structures in five different and partly overlapping regions of Aβ, 1-42 (total), 1-12, 12-26, 18-30, and 28-42, and in five different and partly overlapping regions of IAPP 1-37 (total), 1-7, 7-21, 13-25, and 23-37. Backbone RMSD calculations were performed upon superimposing the structures to the entire residue moiety or upon superimposing to each region individually. Furthermore, we compared the structures of different chains of both Aβ and IAPP within the *principal open* and *closed* conformers. Each pair of chains was superimposed in the region 12-42 for Aβ and region 7-37 for IAPP and the RMSD calculations were performed in the same superimposed region. Finally, we compared the structures of Aβ and IAPP of the *principal open* and *closed* conformers to experimentally resolved structures of both Aβ and IAPP. A selection of the experimentally resolved structures of Aβ and IAPP, based on the online databases of UniProt[27] and Amyloid Atlas[26], was used to conduct this analysis. The experimentally resolved structures for this analysis were chosen based on the following criteria: (1) Only published structures were considered, (2) Only ordered-fibrillar structures were

considered, (3) Only Aβ polymorphs having up to the 42nd residue resolved were considered, (4) Only Aβ polymorphs starting at least from the 12th residue were considered, (5) Only IAPP polymorphs having up to the 37th residue resolved were considered, and (6) Only IAPP polymorphs starting at least from the 13th residue were considered. The structures of the polymorphs were downloaded from the Protein Data Bank (PDB). We superimposed each Aβ and IAPP structure of the heteromeric conformers with each experimentally resolved polymorph of both Aβ and IAPP, and calculated the backbone RMSD between them to determine structural similarities. The superimposing of the structures and the backbone RMSD calculations were performed in the region 18-42 for Aβ and 13-37 for IAPP. Both the RMSD calculations and the superpositions of the structures were conducted using VMD[5].

S.3. Supporting Results

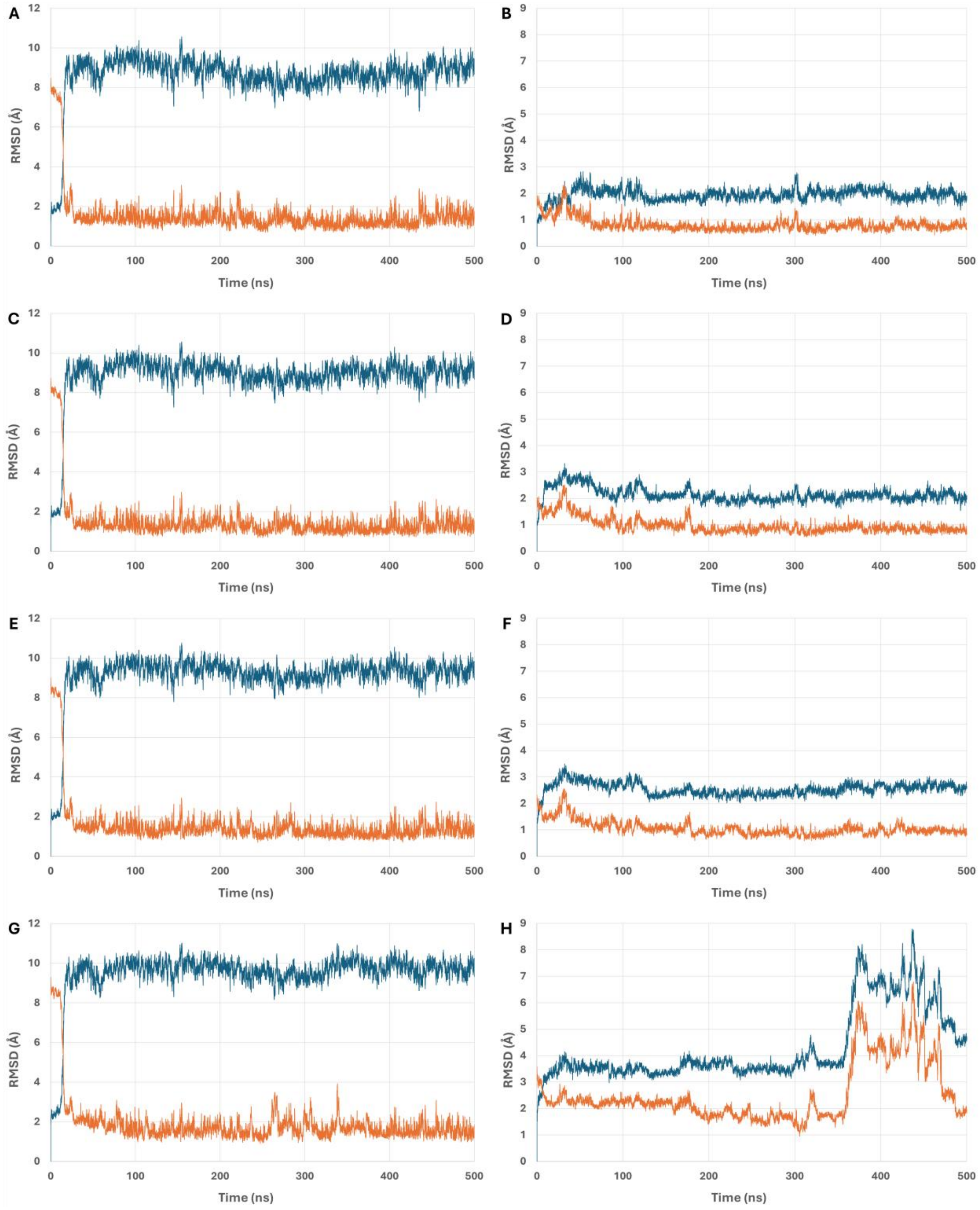

Figure S2: Panels A-B, C-D, E-F, and G-H correspond to the backbone RMSD of chains D-E (junction), C-F (junction + 1 chain beyond), B-G (junction + 2 chains beyond – all chains but the exterior), and A-H (all chains), respectively, with respect to its initial structure (blue) and average structure (orange), as a function of time. Panels on the left correspond to the *open* heteromeric conformer, while panels on the right correspond to the *closed* heteromeric conformer.

Table S4A: Backbone RMSD (Å) between the initial structure of Aβ (5OQV), and the Aβ structures of the heteromeric conformers at the end of the biased MD simulations resulting in the *open* and *closed* conformers, as well as the *principal open* and *closed* conformers from the conventional MD simulations.

	1-42	1-12	12-26	18-30	28-42
Final snapshot of Biased MD which results in the *open* conformer	3.8	3.1 / 1.2	3.3 / 1.8	3.6 / 2.7	4.6 / 1.4
Final snapshot of Biased MD which results in the *closed* conformer	1.9	1.6 / 0.9	1.6 / 1.0	1.8 / 1.0	2.4 / 2.0
Principal open conformer from conventional MD	10.9	17.8 / 5.1	4.0 / 2.3	5.4 / 3.1	7.8 / 2.5
Principal *closed* conformer from conventional MD	1.8	2.0 / 1.7	1.7 / 1.1	1.5 / 1.3	1.7 / 1.4

The 1st row of the table shows 5 different and partly overlapping regions of Aβ, 1-42 (total), 1-12, 12-26, 18-30, and 28-42 for which RMSD calculations were performed. Aβ 1-42 was superimposed to the entire 1-42 residue moiety. In regions 1-12, 12-26, 18-30 and 28-42, RMSD calculations were performed upon superimposing the structures to the entire 1-42 residue moiety (first value before "/") or upon superimposing to each region individually (second value after "/"). Backbone RMSD calculations and backbone superpositions of the structures were conducted using VMD[5].

Table S4B: Backbone RMSD (Å) between the initial (refined) structure of IAPP, and the IAPP structures of the heteromeric conformers at the end of the biased MD simulations resulting in the *open* and *closed* conformers, as well as the *principal open* and *closed* conformers from the conventional MD simulations.

	1-37	1-7	7-21	13-25	23-37
Final snapshot of Biased MD which results in the *open* conformer	5.1	4.2 / 2.2	2.4 / 1.5	3.7 / 2.5	7.1 / 5.2
Final snapshot of Biased MD which results in the *closed* conformer	6.4	3.8 / 1.6	3.6 / 2.4	5.0 / 3.9	8.9 / 5.0
Principal open conformer from conventional MD	6.1	7.0 / 4.0	3.6 / 2.3	5.3 / 2.6	7.3 / 5.7
Principal closed conformer from conventional MD	7.0	9.4 / 5.4	4.1 / 2.6	5.1 / 3.8	8.0 / 4.3

The 1st row of the table shows 5 different and partly overlapping regions of IAPP, 1-37 (total), 1-7, 7-21, 13-25, and 23-37 for which RMSD calculations were performed. The initial (refined) IAPP structure was produced by appending residues 1-5 of 2L86[7] #5 structure to each monomer of the extracted tetramer of 7M65[3], as explained in the supporting methods above. IAPP 1-37 was superimposed to the entire 1-37 residue moiety. In regions 1-7, 7-21, 13-25, and 23-37, RMSD calculations were performed upon superimposing the structures to the entire 1-37 residue moiety (first value before "/") or upon superimposing to each region individually (second value after "/"). Backbone RMSD calculations and backbone superpositions of the structures were conducted using VMD[5].

Table S5A: Backbone RMSD (Å) between the Aβ structures of the heteromeric conformers at the end of the biased MD Simulations resulting in the *open* and *closed* conformers, and the corresponding *principal open* and *closed* conformers from the conventional MD simulations.

	1-42	1-12	12-26	18-30	28-42
Final snapshot of Biased MD which results in the *open* conformer with *principal open* conformer from conventional MD	11.8	19.8 / 4.6	6.0 / 1.8	6.7 / 1.6	6.4 / 2.7
Final snapshot of Biased MD which results in the c*losed conformer* with *principal closed* conformer from conventional MD	1.9	2.5 / 1.8	1.3 / 1.1	1.4 / 1.1	1.8 / 1.3

The 1st row of the table shows 5 different and partly overlapping regions of Aβ, 1-42 (total), 1-12, 12-26, 18-30, and 28-42 for which RMSD calculations were performed. Aβ 1-42 was superimposed to the entire 1-42 residue moiety. In regions 1-12, 12-26, 18-30 and 28-42, RMSD calculations were performed upon superimposing the structures to the entire 1-42 residue moiety (first value before "/") or upon superimposing to each region individually (second value after "/"). Backbone RMSD calculations and backbone superpositions of the structures were conducted using VMD[5].

Table S5B: Backbone RMSD (Å) between the IAPP structures of the heteromeric conformers, at the end of the biased MD Simulations resulting in the open and closed conformers, and the corresponding *principal open and closed* conformers from the conventional MD simulations.

	1-37	1-7	7-21	13-25	23-37
Final snapshot of Biased MD which results in the *open* conformer with *principal open* conformer from conventional MD	3.9	6.8 / 4.1	2.4 / 1.7	2.6 / 1.4	3.0 / 2.0
Final snapshot of Biased MD which results in the c*losed conformer* with *principal closed* conformer from conventional MD	4.3	8.8 / 5.5	2.1 / 1.8	2.1 / 1.3	2.3 / 1.7

The 1st row of the table shows 5 different and partly overlapping regions of IAPP, 1-37 (total), 1-7, 7-21, 13-25, and 23-37 for which RMSD calculations were performed. IAPP 1-37 was superimposed to the entire 1-37 residue moiety. In regions 1-7, 7-21, 13-25, and 23-37, RMSD calculations were performed upon superimposing the structures to the entire 1-37 residue moiety (first value before "/") or upon superimposing to each region individually (second value after "/"). Backbone RMSD calculations and backbone superpositions of the structures were conducted using VMD[5].

Table S6A: Backbone RMSD (Å) between the Aβ structures of the *principal open* and *closed* conformers from the conventional MD simulations.

	1-42	1-12	12-26	18-30	28-42
RMSD (Å)	10.8	17.6 / 4.7	5.1 / 2.1	6.1 / 3.2	7.4 / 2.9

The 1st row of the table shows 5 different and partly overlapping regions of Aβ, 1-42 (total), 1-12, 12-26, 18-30, and 28-42 for which RMSD calculations were performed. Aβ 1-42 was superimposed to the entire 1-42 residue moiety. In regions 1-12, 12-26, 18-30 and 28-42, RMSD calculations were performed upon superimposing the structures to the entire 1-42 residue moiety (first value before "/") or upon superimposing to each region individually (second value after "/"). Backbone RMSD calculations and backbone superpositions of the structures were conducted using VMD[5].

Table S6B: Backbone RMSD (Å) between the IAPP structures of the *principal open* and *closed* conformers from the conventional MD simulations.

	1-37	1-7	7-21	13-25	23-37
RMSD (Å)	5.5	9.6 / 4.6	3.3 / 2.5	3.0 / 1.7	4.7 / 2.6

The 1st row of the table shows 5 different and partly overlapping regions of IAPP, 1-37 (total), 1-7, 7-21, 13-25, and 23-37 for which RMSD calculations were performed. IAPP 1-37 was superimposed to the entire 1-37 residue moiety. In regions 1-7, 7-21, 13-25, and 23-37, RMSD calculations were performed upon superimposing the structures to the entire 1-37 residue moiety (first value before "/") or upon superimposing to each region individually (second value after "/"). Backbone RMSD calculations and backbone superpositions of the structures were conducted using VMD[5].

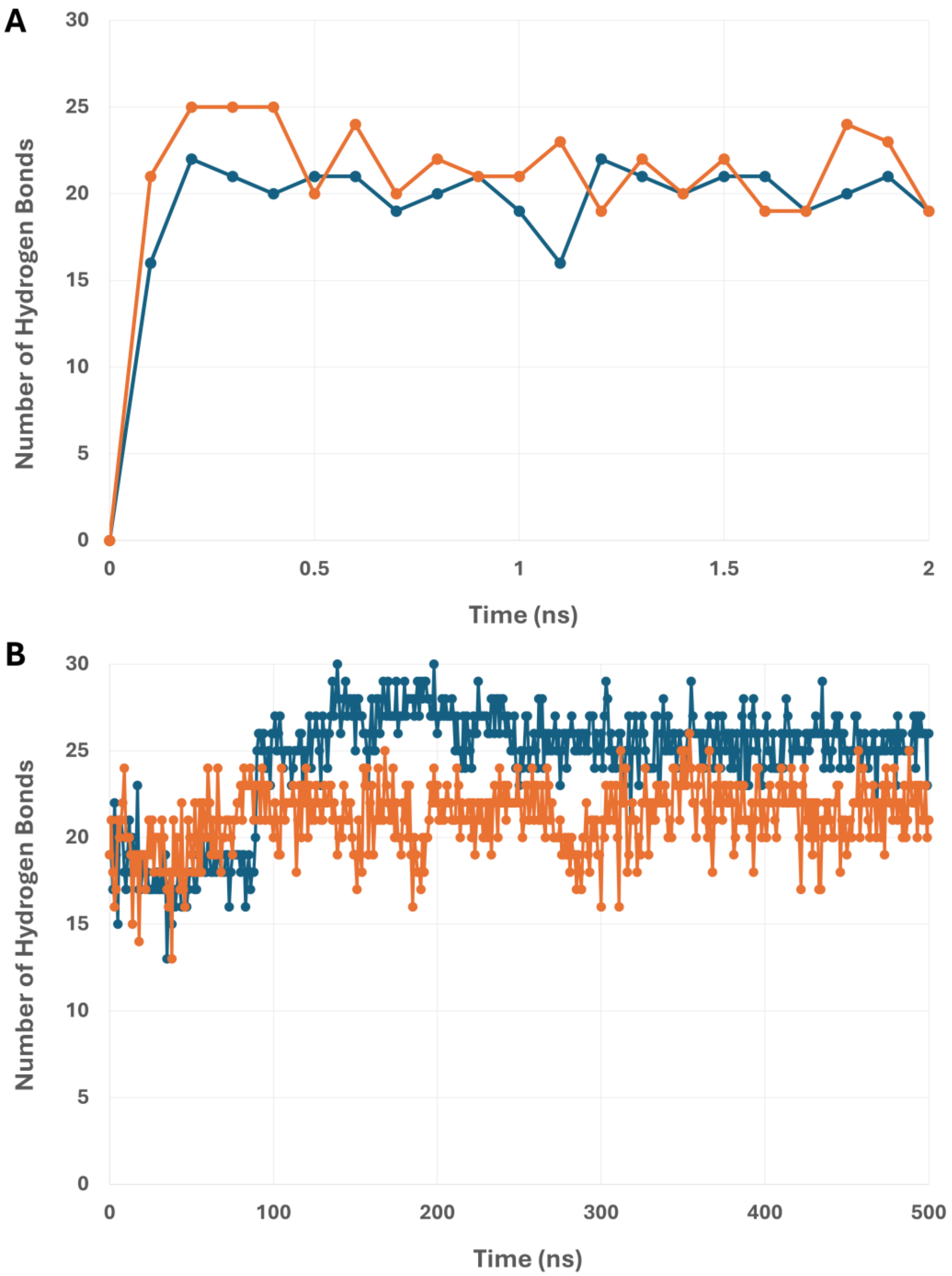

Figure S3: Panels A and B show the number of backbone-backbone hydrogen bonds in the junction of the *open* (blue) and *closed* (orange) heteromeric conformers during the biased and conventional MD simulations, respectively.

```
IAPP     -----KCNTATCATQRLANFLVHSSNNFGAILSSTNVGSNTY   37
Aβ       DAEFRHDSGYEVHHQKLVFFAEDVGSNKGAIIGLMVGGVVIA   42
```

```
IAPP     -----KCNTATCATQRLANFLV-HSSNNFGAILSSTNVGSNTY  37
Aβ       DAEFRHDSGYEVHHQKLV-FFAEDVGSNKGAIIG-LMVGGVVIA 42
```

```
IAPP     -----KCNTATCATQRLANFLVH-SSNNFGAILSSTNVGSNTY  37
Aβ       DAEFRHDSGYEVHHQKLV-FFAEDVGSNKGAIIG-LMVGGVVIA 42
```

Figure S4: The first alignment corresponds to the sequence alignment that we enforced during the biased MD simulations. The second and third alignment correspond to the alignment according to side chain complementarity and β-sheet hydrogen bonds after the conventional MD simulations for the *open* and *closed* conformers, respectively.

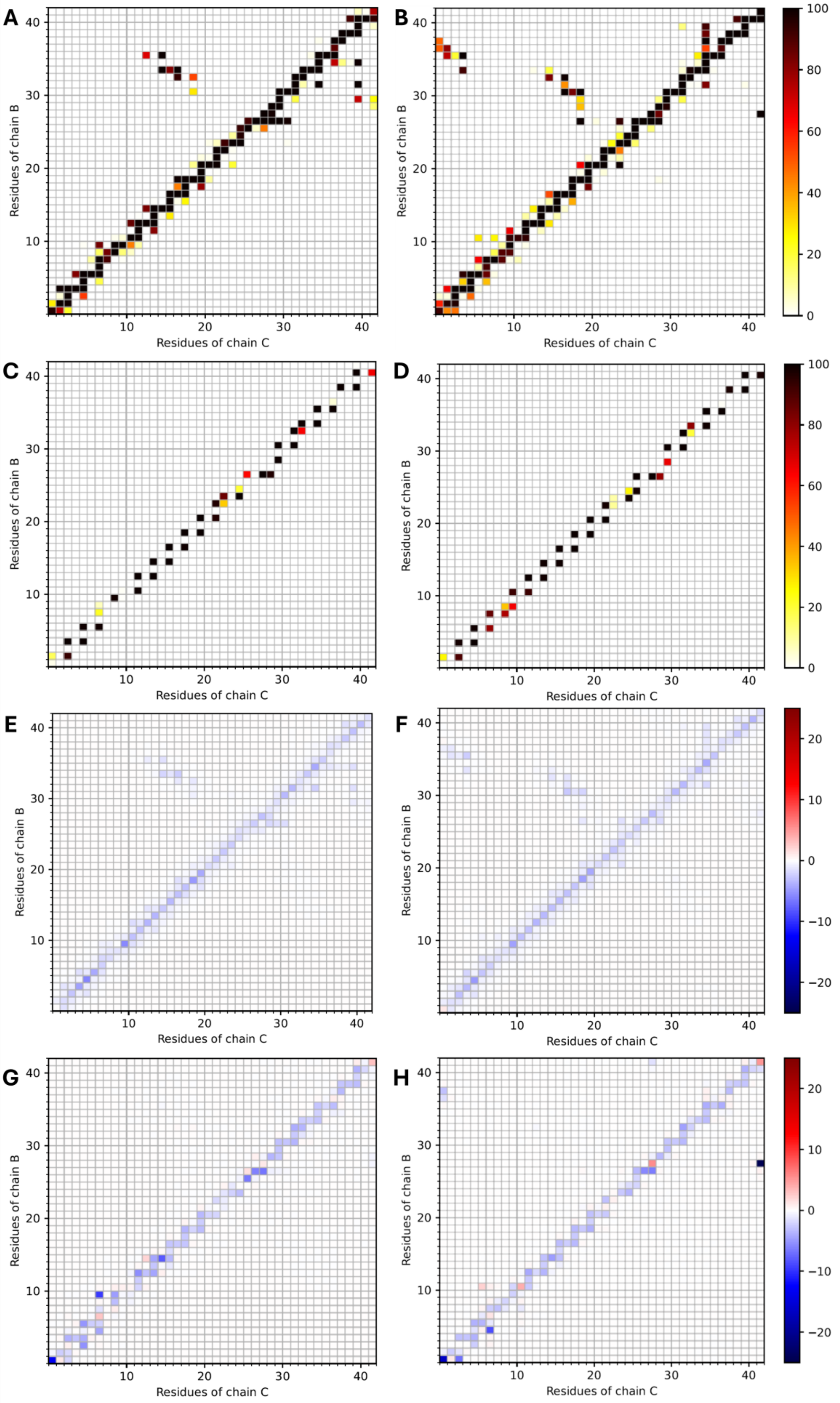
A
B
C
D
E
F
G
H
Residues of chain B
Residues of chain C
10
20
30
40
100
80
60
40
20
0
-10
-20

Figure S5: Panels A-B, C-D, E-F, and G-H show contact maps, backbone-backbone hydrogen-bond maps, non-polar interaction free energy maps, and polar interaction free energy maps, respectively, between residues of chains B and C of homomeric Aβ within the two heteromeric conformers. Panels on the left correspond to the *open* heteromeric conformer, while panels on the right correspond to the *closed* heteromeric conformer. The values in panels A-D correspond to percentage probabilities/occupancies, while the values in panels E-H correspond to interaction free energies in kcal/mol.

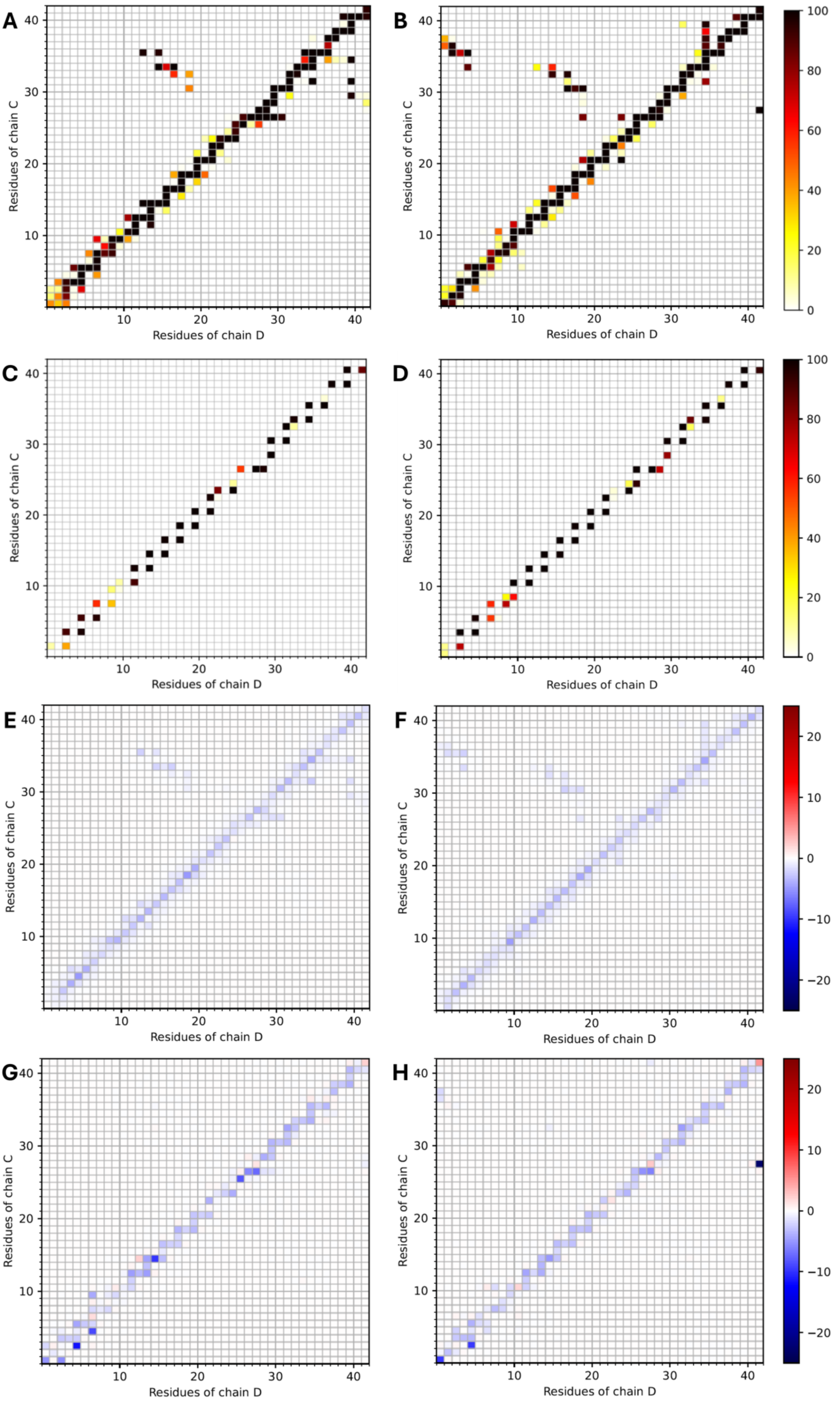
A
B
C
D
E
F
G
H
Residues of chain C
Residues of chain D
10
20
30
40
100
80
60
40
20
0
−10
−20

Figure S6: Panels A-B, C-D, E-F, and G-H show contact maps, backbone-backbone hydrogen-bond maps, non-polar interaction free energy maps, and polar interaction free energy maps, respectively, between residues of chains C and D of homomeric Aβ within the two heteromeric conformers. Panels on the left correspond to the *open* heteromeric conformer, while panels on the right correspond to the *closed* heteromeric conformer. The values in panels A-D correspond to percentage probabilities/occupancies, while the values in panels E-H correspond to interaction free energies in kcal/mol.

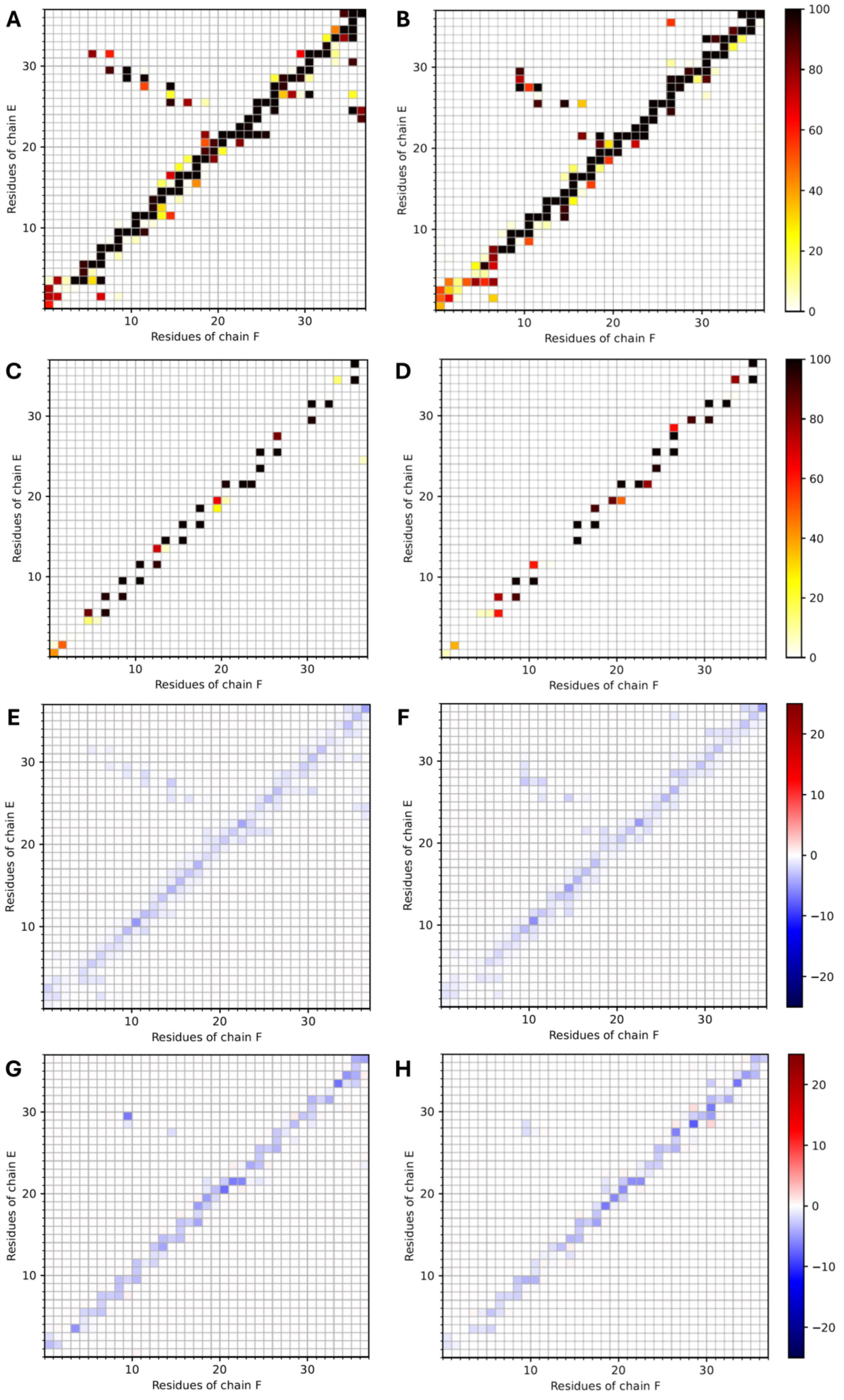

A
B
C
D
E
F
G
H
Residues of chain E
Residues of chain F

Figure S7: Panels A-B, C-D, E-F, and G-H show contact maps, backbone-backbone hydrogen-bond maps, non-polar interaction free energy maps, and polar interaction free energy maps, respectively, between residues of chains E and F of homomeric IAPP within the two heteromeric conformers. Panels on the left correspond to the *open* heteromeric conformer, while panels on the right correspond to the *closed* heteromeric conformer. The values in panels A-D correspond to percentage probabilities/occupancies, while the values in panels E-H correspond to interaction free energies in kcal/mol.

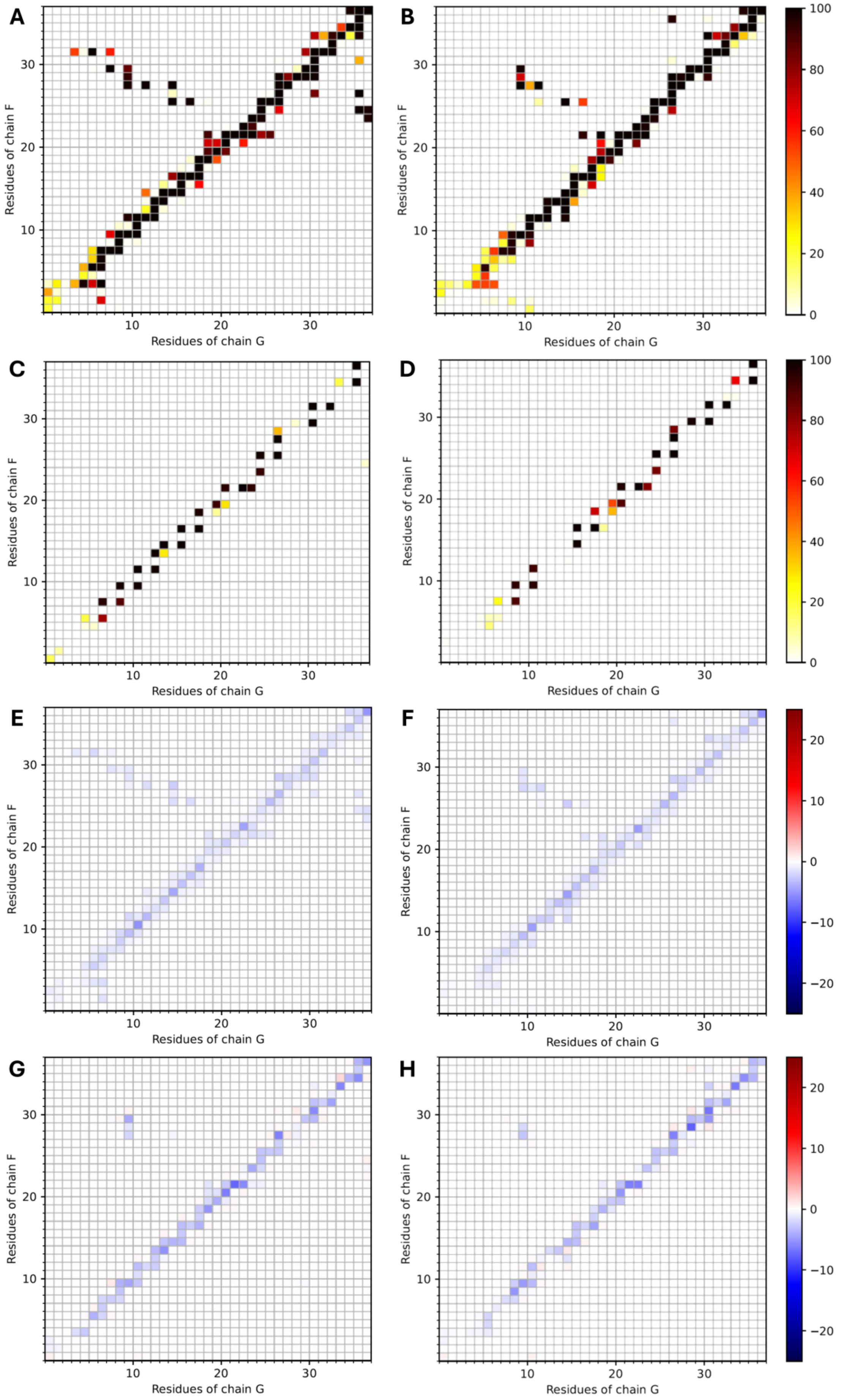
A
B
C
D
E
F
G
H
Residues of chain F
Residues of chain G
10
20
30
100
80
60
40
20
0
−10
−20

Figure S8: Panels A-B, C-D, E-F, and G-H show contact maps, backbone-backbone hydrogen-bond maps, non-polar interaction free energy maps, and polar interaction free energy maps, respectively, between residues of chains F and G of homomeric IAPP within the two heteromeric conformers. Panels on the left correspond to the *open* heteromeric conformer, while panels on the right correspond to the *closed* heteromeric conformer. The values in panels A-D correspond to percentage probabilities/occupancies, while the values in panels E-H correspond to interaction free energies in kcal/mol.

Table S7A : The key interactions taking place in the junction of the open heteromeric conformer.

Aβ-IAPP	Interactions	Comments on interactions between Aβ and IAPP
V12-C7	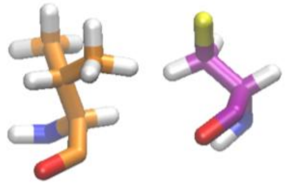	Non-polar interactions between CG1 of V12 and CB of C7
H13-A8	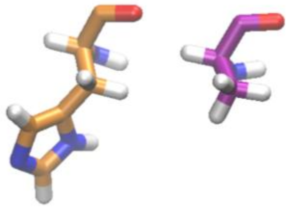	Non-polar interactions between CB of H13 and CB of A8
H14-T9	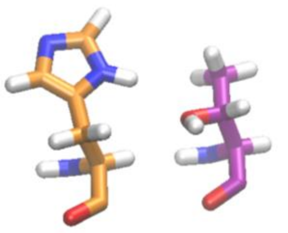	Hydrogen Bond between ND1 of H14 and OG1 of T9
Q15-Q10	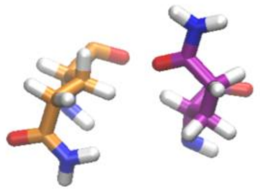	Non-polar interaction between CB of Q15 and CB of Q10

Q15-T9, Q15-Q10	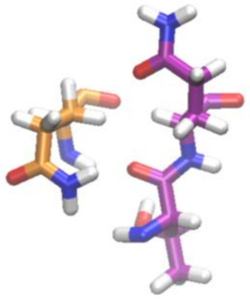	Hydrogen Bond between NE2 of Q15 and O (backbone) of T9
K16-R11	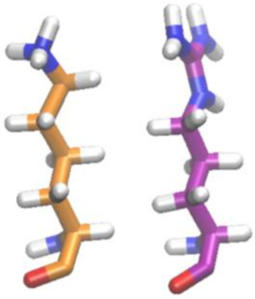	Non-polar interactions between the non-polar moieties of K16 and R11
L17-L12	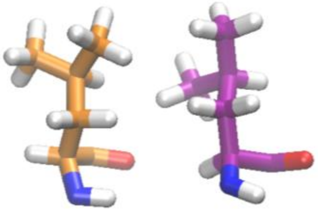	Non-polar (hydrophobic) interactions between the hydrophobic moieties of L17 and L12
V18-A13	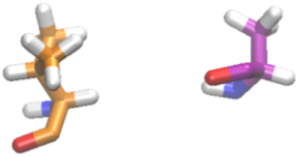	Weak non-polar (hydrophobic) interactions

V18-A13	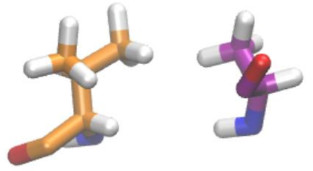	Non-polar (hydrophobic) interactions between CG2 of V18 and CB of A13<br><br>The particular interaction was taken from an alternative snapshot of the open conformer simulation within the main cluster
F19-F15	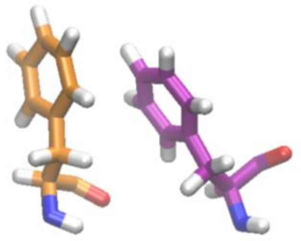	Non-polar (π-π parallel stacking) interactions between F19 and F15
F20-L16	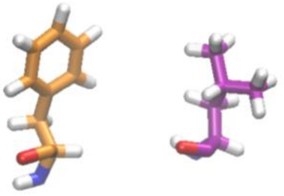	Weak non-polar (aromatic-hydrophobic) interactions between the ring of F20 and L16
F20-L16	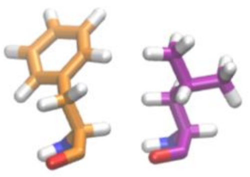	Non-polar (aromatic-hydrophobic) interactions between the ring of F20 and CD2 of L16<br><br>The particular interaction was taken from an alternative snapshot of the open conformer simulation within the main cluster
A21-V17	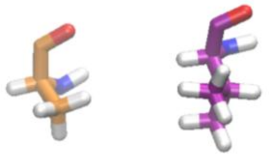	Weak non-polar (hydrophobic) interactions

A21-V17	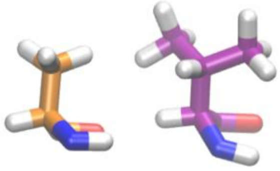	Non-polar (hydrophobic) interactions between CB of A21 and CG2 of V17<br><br>The particular interaction was taken from an alternative snapshot of the open conformer simulation within the main cluster
E22-H18	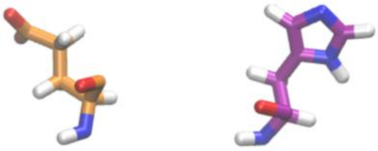	Weak non-polar interactions
E22-H18	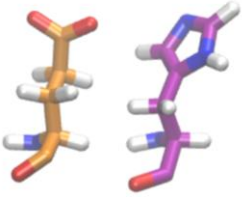	Non-polar interactions between CB of E22 and CB of H18<br><br>Non-polar (hydrophobic-imidazole) interactions between CG of E22 and imidazole of H18<br><br>The particular interaction was taken from an alternative snapshot of the open conformer simulation within the main cluster
D23-H18	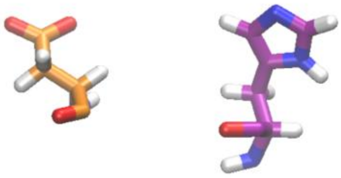	Weak non-polar interactions
D23-H18	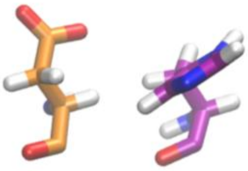	Non-polar interactions between CA (backbone) of D23 and CB of H18.<br><br>The particular interaction was taken from an alternative snapshot of the open conformer simulation within the main cluster

V24-S19	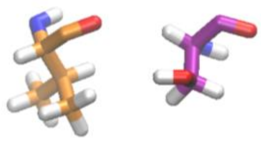	Non-polar interactions between CG1 of V24 and CB of S19
G25-S20	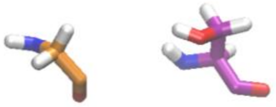	Weak non-polar interactions
G25-S19, G25-S20	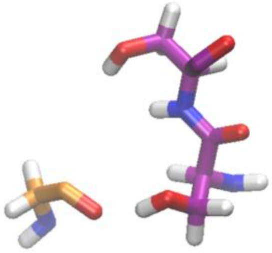	Hydrogen Bond between O (backbone) of G25 and OG of S19
S26-N21	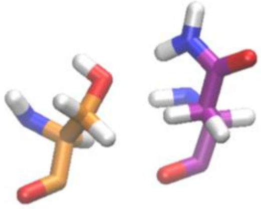	Hydrogen Bond between OG of S26 and ND2 of N21<br><br>Non-polar interactions between CB of S26 and CB of N21

N27-N22	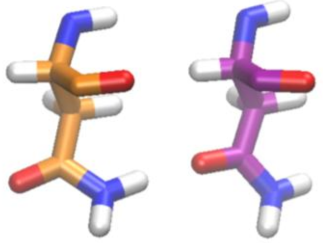	Hydrogen Bond between ND2 of N27 and OD1 of N22<br><br>Non-polar interactions between CB of N27 and CB of N22
N27-A25	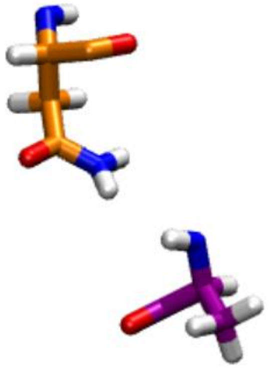	Hydrogen Bond between ND2 of N27 and N (backbone) of A25<br><br>Hydrogen Bond between ND2 of N27 and O (backbone) of A25
K28-F23	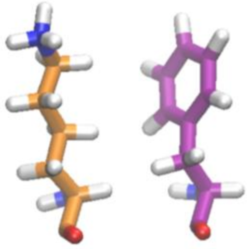	Non-polar interactions (hydrophobic-aromatic) between the non-polar moieties of K28 and the ring of F23
G29-G24	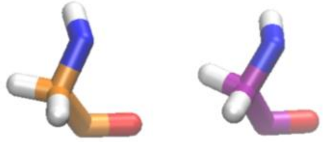	Non-polar interactions between CA (backbone) of G29 and CA (backbone) of G24

A30-A25	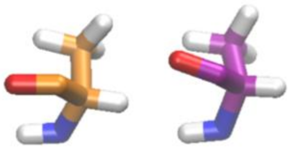	Non-polar (hydrophobic) interactions between CB of A30 and CB of A25
I31-I26	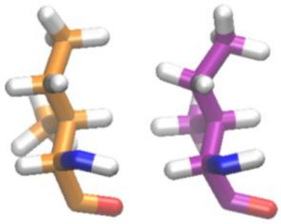	Non-polar (hydrophobic) interactions between the non-polar (hydrophobic) moieties of I31 and I26
I32-L27	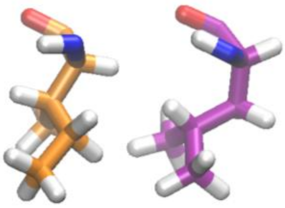	Non-polar (hydrophobic) interactions between the non-polar (hydrophobic) moieties of I32 and L27
G33-S28	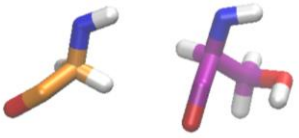	Non-polar interactions between CA (backbone) of G33 and CA (backbone) of S28

L34-T30	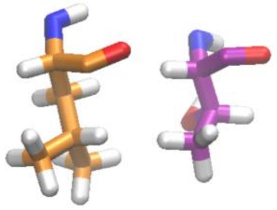	Non-polar interactions between the non-polar (hydrophobic) moieties of L34 and non-polar moieties of T30
M35-N31	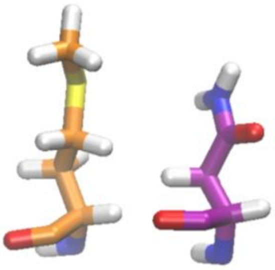	Non-polar interactions between CB of M35 and CB of N31<br><br>Non-polar interactions between CG of M35 and CG of N31
V36-V32	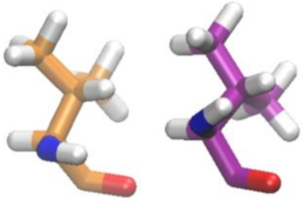	Non-polar (hydrophobic) interactions between the non-polar (hydrophobic) moieties of V36 and V32
G37-G33	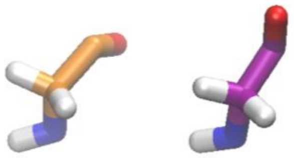	Non-polar interactions between CA (backbone) of G37 and CA (backbone) of G33

G38-S34	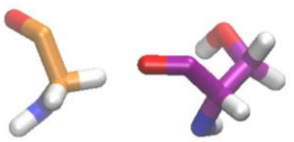	Non-polar interactions between CA (backbone) of G38 and CA (backbone) of S34
V39-N35	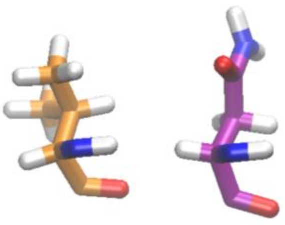	Non-polar interactions between CB of V39 and CB of N35<br><br>Non-polar interactions between CG2 of V39 and CG of N35
V40-T36	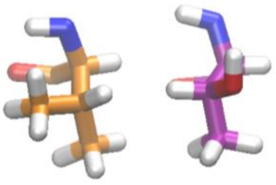	Non-polar interactions between CB of V40 and CB of T36<br><br>Non-polar interactions between CG2 of V40 and CG2 of T36
I41-Y37	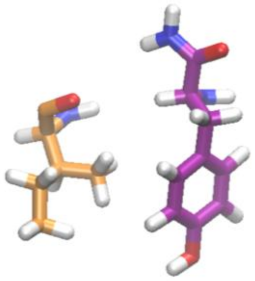	Non-polar (hydrophobic-aromatic) interaction between the non-polar (hydrophobic) moieties of I41 and the ring of Y37<br><br>Hydrogen Bond between O (backbone) of I41 and NT (amide) of Y37

The first column corresponds to the Aβ-IAPP residue pair under investigation. The second column corresponds the structure of the residue pair visualized and captured using VMD[5]. All atoms are shown in licorice representation, with the carbon atoms of Aβ colored in orange, the carbon atoms of IAPP colored in purple and all the other atoms colored by type. The third column corresponds to comments regarding the type of interactions between the residue pairs. All residue pair structures are extracted from the *principal open conformer* unless otherwise specified in the comments.

Table 7B: The key interactions taking place in the junction of the closed heteromeric conformer.

Aβ-IAPP	Interactions	Comments on interactions between Aβ and IAPP
Y10-A5	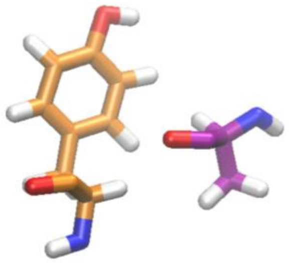	Non-polar interactions (aromatic-hydrophobic) between the ring of Y10 and both CA (backbone) and CB of A5<br><br>Non-polar interactions between CB of Y10 and CA (backbone) of A5
E11-T6	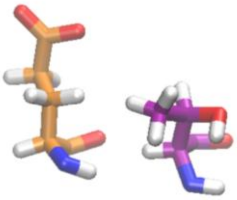	Non-polar interactions between CB of E11 and CG2 of T6
V12-C7	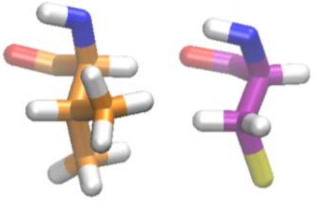	Non-polar interactions between the non-polar (hydrophobic) moieties of V12 and non-polar moieties of C7
H13-A8	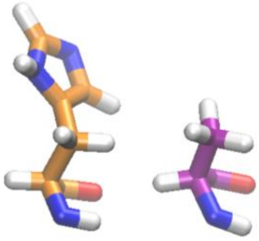	Non-polar interactions between CB of H13 and CB of A8<br><br>Non-polar interactions (imidazole-hydrophobic) between the ring of H13 and CB of A8

H14-T9	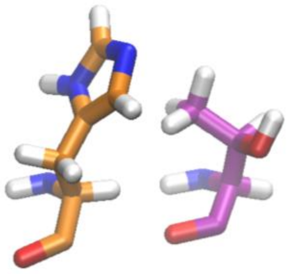	Non-polar interaction (imidazole-hydrophobic) between the ring of H14 and CG2 of T9
Q15-Q10	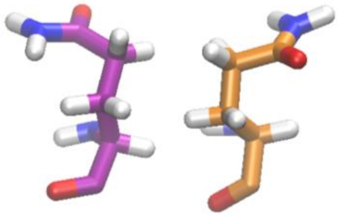	Non-polar interactions between CB of Q15 and CB of Q10<br><br>Non-polar interactions between CG of Q15 and CG of Q10<br><br>Hydrogen Bond between OE1 of Q10 and NE2 of Q10. (Although not present in *principal closed* conformer, this hydrogen bond has 37% occupancy throughout all the conformers of the primary cluster of the *closed* heteromeric conformer)
K16-R11	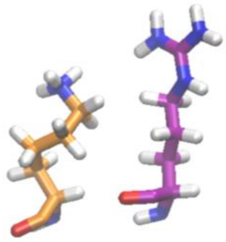	Non-polar interactions between the non-polar moieties of K16 and R11
L17-L12	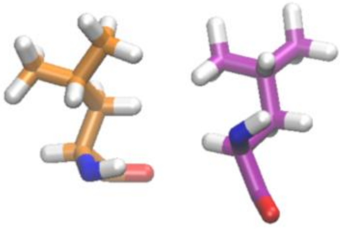	Non-polar (hydrophobic) interactions between CB of L17 and CD2 of L12<br><br>Non-polar (hydrophobic) interactions between CD1 of L17 and CD2 of L12

V18-A13	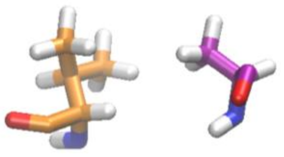	Non-polar (hydrophobic) interactions between CG2 of V18 and CB of A13
F19-F15	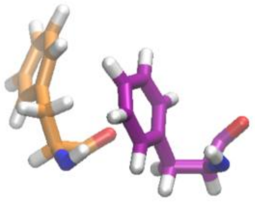	Non-polar (π-π t-shape stacking) interactions between F19 and F15
F20-L16	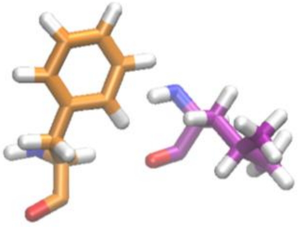	Non-polar (aromatic-hydrophobic) interactions between the ring of F20 and CB of L16
A21-V17	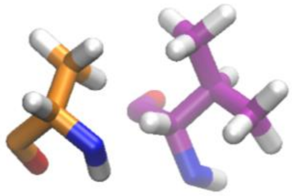	Non-polar (hydrophobic) interactions between CB of A21 and CG1 of V17

E22-H18	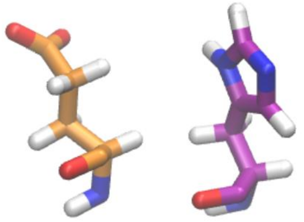	Non-polar interactions between CA (backbone) of E22 and CB of H18
V24-S19	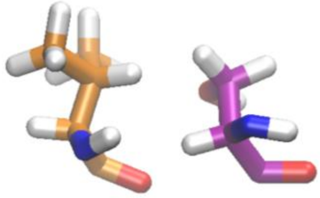	Non-polar interactions between CB of V24 and CB of S19<br><br>Hydrogen Bond between O (backbone) of V24 and OG of S19. (Although not present in *principal closed* conformer, this hydrogen bond has 29% occupancy throughout all the conformers of the primary cluster of the *closed* heteromeric conformer)
G25-S20	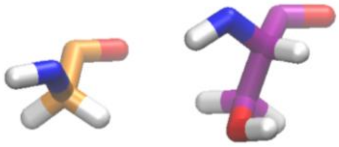	Non-polar interactions between CA (backbone) of G25 and CB of S20
S26-N21	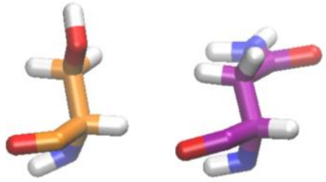	Non-polar interactions between CB of S26 and CB of N21

N27-N22	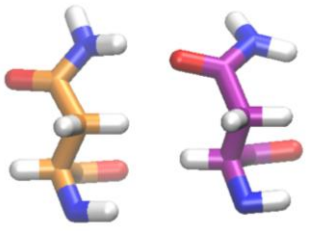	Non-polar interactions between CB of N27 and CB of N22<br><br>Hydrogen Bond between ND2 of N27 and OD1 of N22
N27-S19	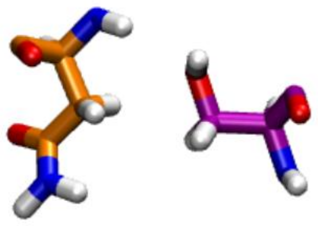	Hydrogen Bond between N (backbone) of N27 and OG of S19
K28-F23	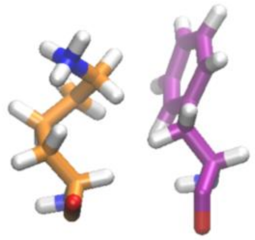	Non-polar interactions between the non-polar moieties of K28 and CB of F23<br><br>Non-polar (hydrophobic-aromatic) interactions between the non-polar moieties of K28 and the ring of F23
G29-G24	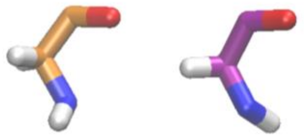	Non-polar interactions between CA (backbone) of G29 and CA (backbone) of G24

A30-A25	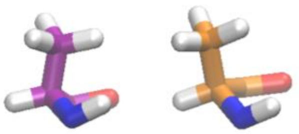	Non-polar (hydrophobic) interactions between CB of A30 and CB of A25
I31-I26	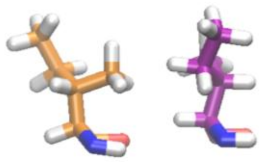	Non-polar (hydrophobic) interactions between the non-polar (hydrophobic) moieties of I31 and I26
I32-L27	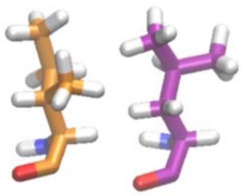	Non-polar (hydrophobic) interactions between the non-polar (hydrophobic) moieties of I32 and L27
G33-S28	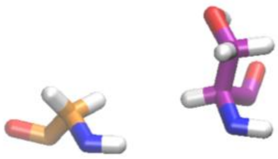	Non-polar interactions between CA (backbone) of G33 and CA (backbone) of S28

G33-Q10	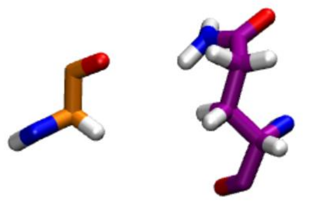	Hydrogen Bond between O (backbone) of G33 and NE2 of Q10
L34-T30	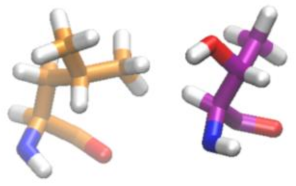	Non-polar interactions between CD1 of L34 and both CB and CG2 of T30
L34-N31, M35-N31	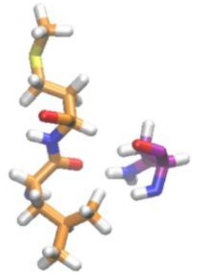	Hydrogen Bond between O (backbone) of L34 and ND2 of N31
M35-N31	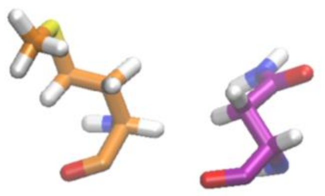	Non-polar interactions between CB of M35 and CB of N31

V36-V32	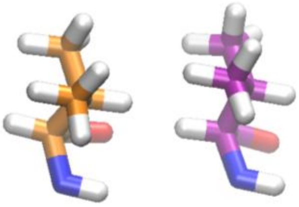	Non-polar (hydrophobic) interactions between the non-polar (hydrophobic) moieties of V36 and V32
G37-G33	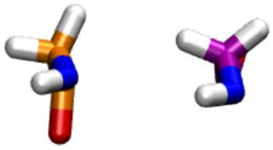	Non-polar interactions between CA (backbone) of G37 and CA (backbone) of G33
G38-S34	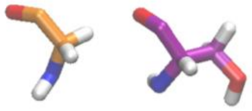	Non-polar interactions between CA (backbone) of G38 and CA (backbone) of S34
V39-N35	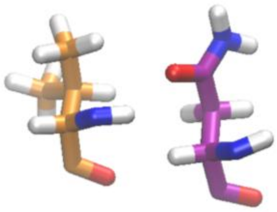	Non-polar interactions between CB of V39 and CB of N35<br><br>Hydrogen Bond between N (backbone) of V39 and OD1 of N35

V40-T36	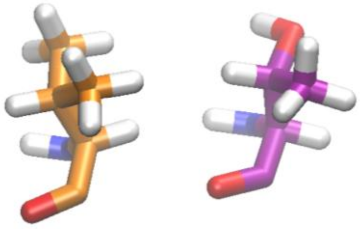	Non-polar interactions between CG1 of V40 and CG2 of T36
I41-Y37	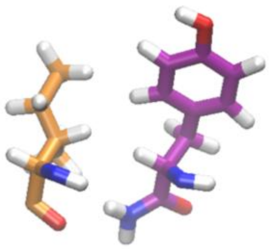	Non-polar (hydrophobic) interactions between the non-polar (hydrophobic) moieties of I41 and CB of Y37<br>Non-polar (hydrophobic-aromatic) interactions between the non-polar (hydrophobic) moieties of I41 and the ring of Y37<br>Hydrogen Bond between O (backbone) of I41 and NT (amide) of Y37

The first column corresponds to the Aβ-IAPP residue pair under investigation. The second column corresponds the structure of the residue pair visualized and captured using VMD[5]. All atoms are shown in licorice representation, with the carbon atoms of Aβ colored in orange, the carbon atoms of IAPP colored in purple and all the other atoms colored by type. The third column corresponds to comments regarding the type of interactions between the residue pairs. All residue pair structures are extracted from the *principal closed conformer* unless otherwise specified in the comments.

Table S8: Side chain-side chain or backbone-side chain hydrogen bonds within neighboring chains of both the homomeric regions (excluding exterior peptides) and the junction of open and closed heteromeric conformers.

open			*closed*		
chain B	chain C	occupancy (%)	chain B	chain C	occupancy (%)
D1-Side-OD1	D1-Main-N	43	D1-Side-OD1	D1-Main-N	43
D1-Side-OD2	D1-Main-N	45	D1-Side-OD2	D1-Main-N	49
H6-Side-ND1	R5-Main-O	41	D1-Main-N	E3-Side-OE1	24
S8-Side-OG	D7-Main-O	24	H6-Main-O	H6-Side-ND1	23
Y10-Side-OH	D7-Side-OD2	21	S8-Side-OG	S8-Side-OG	20
H13-Side-ND1	V12-Main-O	32	Q15-Side-NE2	Q15-Side-OE1	21
Q15-Side-NE2	H14-Main-O	33	N27-Side-ND2	N27-Side-OD1	95
Q15-Side-NE2	Q15-Side-OE1	85	K28-Side-NZ	A42-Side-OT1	70
S26-Side-OG	S26-Main-O	60	K28-Side-NZ	A42-Side-OT2	68
N27-Side-ND2	N27-Side-OD1	97	G38-Main-N	D1-Side-OD1	31
N27-Side-ND2	A30-Main-N	34	G38-Main-N	D1-Side-OD2	23
N27-Side-ND2	A30-Main-N	35			
N27-Side-ND2	A30-Main-O	77			
chain C	chain D	occupancy (%)	chain C	chain D	occupancy (%)
D1-Side-OD2	D1-Main-N	21	D1-Side-OD1	D1-Main-N	25
E3-Side-OE1	R5-Side-NH1	27	D1-Side-OD2	D1-Main-N	28
E3-Side-OE1	R5-Side-NH2	22	S8-Main-N	S8-Side-OG	21
E3-Side-OE2	R5-Side-NH1	32	Q15-Side-OE1	Q15-Side-NE2	40
E3-Side-OE2	R5-Side-NH2	25	N27-Side-ND2	N27-Side-OD1	97
R5-Side-NH1	D7-Side-OD1	26	K28-Side-NZ	A42-Side-OT1	55
R5-Side-NH1	D7-Side-OD2	24	K28-Side-NZ	A42-Side-OT2	40
H6-Side-ND1	R5-Main-O	45	G33-Main-O	Q15-Side-NE2	22
S8-Side-OG	D7-Main-O	28			
Q15-Side-NE2	H14-Main-O	43			
Q15-Side-NE2	Q15-Side-OE1	100			
S26-Side-OG	S26-Main-O	75			
N27-Side-ND2	N27-Side-OD1	99			
N27-Side-ND2	A30-Main-N	33			
N27-Side-ND2	A30-Main-N	37			
N27-Side-ND2	A30-Main-O	66			
chain D	chain E	occupancy (%)	chain D	chain E	occupancy (%)
H14-Side-ND1	T9-Side-OG1	68	Q15-Side-OE1	Q10-Side-NE2	37
Q15-Side-NE2	T9-Main-O	88	V24-Main-O	S19-Side-OG	29
G25-Main-O	S19-Side-OG	22	N27-Main-N	S19-Side-OG	57
S26-Side-OG	N21-Main-O	33	N27-Side-ND2	N22-Side-OD1	94
N27-Side-ND2	N22-Side-OD1	97	G33-Main-O	Q10-Side-NE2	30
N27-Side-ND2	A25-Main-N	27	L34-Main-O	N31-Side-ND2	69
N27-Side-ND2	A25-Main-O	48	V39-Main-N	N35-Side-OD1	99
I41-Main-O	Y37-Side-NT	49	I41-Main-O	Y37-Side-NT	72

chain E	chain F	occupancy (%)
T4-Side-OG1	T4-Main-O	97
T4-Side-OG1	A5-Main-O	23
N14-Side-OD1	N14-Side-ND2	52
S19-Side-OG	H18-Main-O	60
S20-Main-O	S19-Side-OG	53
N21-Side-OD1	S20-Side-OG	33
N21-Side-OD1	N21-Main-N	72
N21-Side-OD1	N21-Side-ND2	64
N22-Main-N	S19-Side-OG	34
N22-Side-ND2	N22-Side-OD1	99
N22-Side-ND2	A25-Main-N	20
N22-Side-ND2	A25-Main-N	21
N22-Side-ND2	A25-Main-O	42
S28-Side-OG	S28-Side-OG	23
S29-Side-OG	S29-Main-O	29
T30-Main-N	Q10-Side-OE1	26
T30-Side-OG1	Q10-Side-OE1	90
N31-Side-ND2	T36-Side-OG1	50
S34-Side-OG	S34-Main-O	81
S34-Side-OG	T36-Side-OG1	71
N35-Main-N	N35-Side-OD1	40
N35-Side-ND2	N35-Side-OD1	40
T36-Side-OG1	T36-Side-OG1	27
Y37-Main-O	Y37-Side-NT	75

chain E	chain F	occupancy (%)
T4-Side-OG1	T4-Main-O	21
T4-Side-OG1	T6-Main-N	40
N14-Side-OD1	N14-Main-N	86
S19-Main-O	S19-Side-OG	86
S20-Main-O	S19-Side-OG	64
N21-Side-OD1	N21-Side-ND2	70
N22-Main-N	S19-Side-OG	71
N22-Side-ND2	N22-Side-OD1	98
S29-Main-O	Q10-Side-NE2	59
N31-Side-OD1	N31-Side-ND2	77
S34-Side-OG	N31-Main-O	59
S34-Side-OG	G33-Main-N	65
S34-Side-OG	G33-Main-N	71
S34-Side-OG	S34-Main-N	98
N35-Main-N	N35-Side-OD1	27
N35-Side-ND2	N35-Side-OD1	40
Y37-Main-O	Y37-Side-NT	77

chain F	chain G	occupancy (%)
T4-Side-OG1	T4-Main-O	25
T4-Side-OG1	A5-Main-O	48
Q10-Side-NE2	T9-Main-O	40
Q10-Side-NE2	Q10-Side-OE1	22
N14-Side-OD1	N14-Side-ND2	67
S19-Main-O	S19-Side-OG	46
S20-Main-O	S19-Side-OG	36
N21-Side-OD1	N21-Main-N	34
N21-Side-OD1	N21-Side-ND2	70
N22-Main-N	S19-Side-OG	39
N22-Side-ND2	N22-Side-OD1	100
S28-Main-O	Q10-Side-NE2	89
T30-Main-N	Q10-Side-OE1	46
T30-Side-OG1	Q10-Side-OE1	67
N31-Side-OD1	N31-Side-ND2	26
S34-Side-OG	N31-Main-O	21
S34-Side-OG	G33-Main-N	28
S34-Side-OG	G33-Main-N	45

chain F	chain G	occupancy (%)
T4-Side-OG1	T6-Main-N	28
T9-Side-OG1	T9-Main-O	35
T9-Side-OG1	T9-Side-OG1	23
Q10-Side-NE2	Q10-Side-OE1	39
N14-Side-OD1	N14-Main-N	89
S19-Main-O	S19-Side-OG	24
S20-Main-O	S19-Side-OG	23
S20-Main-O	N21-Side-ND2	21
N21-Side-OD1	N21-Side-ND2	70
N22-Main-N	S19-Side-OG	38
N22-Side-ND2	N22-Side-OD1	89
S28-Main-O	Q10-Side-NE2	42
S28-Side-OG	L27-Main-O	21
T30-Side-OG1	Q10-Side-NE2	24
T30-Side-OG1	Q10-Side-NE2	34
N31-Side-OD1	N31-Side-ND2	95
S34-Side-OG	N31-Main-O	46
S34-Side-OG	G33-Main-N	46

S34-Side-OG	S34-Main-N	53	S34-Side-OG	G33-Main-N	68
S34-Side-OG	S34-Main-O	32	S34-Side-OG	S34-Main-N	78
S34-Side-OG	T36-Side-OG1	28	N35-Main-N	N35-Side-OD1	32
N35-Side-ND2	N35-Side-OD1	33	N35-Side-ND2	N35-Side-OD1	46
Y37-Main-O	Y37-Side-NT	86	Y37-Main-O	Y37-Side-NT	67

The table shows side chain-side chain or backbone-side chain hydrogen bond occupancies between atom pairs in neighboring chains (i.e., Chain B-Chain C, …, Chain F-Chain G). The occupancies between atom pairs in neighboring chains A-B and G-H are omitted. The occupancies were calculated using simulation snapshots within the main clusters from the *open* heteromeric conformer (three columns on the left), and the *closed* heteromeric conformer (three columns on the right). The first two columns (on the left and on the right) correspond to the atom pairs involved in the hydrogen bonds (e.g., T4-Side-OG1, where T: one-letter code for Threonine, 4: position in the sequence, Side: Side chain (could be Main: Backbone), and OG1: Atom name). The third column (on the left and on the right) corresponds to the percentage occupancy of the hydrogen bonds. Only hydrogen bonds with occupancies ≥ 20% are shown. Hydrogen bonds in the junction (chain D - chain E) formed between pairs of residues participating in β-sheets are highlighted in green, and hydrogen bonds in the junction formed between pairs of residues adjacent to β-sheets are highlighted in light green. The calculations were conducted using VMD[5]. Hydrogen Bond Tool with a cutoff distance of 3.5 Å and cutoff angle of 90°.

Table S9A: Backbone RMSD (Å) between all pairs of chains within the *principal open* conformer.

	chain A	chain B	chain C	chain D	chain E	chain F	chain G	chain H
chain A	0.0	0.9	1.3	1.8	2.6	2.9	3.1	2.9
chain B	0.9	0.0	0.6	1.1	2.3	2.5	2.6	2.5
chain C	1.3	0.6	0.0	0.7	2.1	2.3	2.4	2.4
chain D	1.8	1.1	0.7	0.0	2.1	2.1	2.2	2.2
chain E	2.6	2.3	2.1	2.1	0.0	0.8	1.1	2.0
chain F	2.9	2.5	2.3	2.1	0.8	0.0	0.6	1.8
chain G	3.1	2.6	2.4	2.2	1.1	0.6	0.0	1.7
chain H	2.9	2.5	2.4	2.2	2.0	1.8	1.7	0.0

Each pair of chains was superimposed in the region 12-42 for Aβ and region 7-37 for IAPP. The backbone RMSD calculations were performed in the same superimposed region. Rows and columns both correspond to chains A-H of the *open* heteromeric conformer. The backbone RMSD values reported in the tables are symmetric with diagonal values equal to zero (0.0) as they correspond to the backbone RMSD between a chain and itself.

Table S9B: Backbone RMSD (Å) between all pairs of chains within the *principal closed* conformer.

	chain A	chain B	chain C	chain D	chain E	chain F	chain G	chain H
chain A	0.0	0.7	0.9	1.0	3.1	3.3	3.7	4.1
chain B	0.7	0.0	0.5	0.8	3.0	3.2	3.7	4.1
chain C	0.9	0.5	0.0	0.4	2.8	3.1	3.6	4.0
chain D	1.0	0.8	0.4	0.0	2.7	3.0	3.4	3.8
chain E	3.1	3.0	2.8	2.7	0.0	1.0	1.7	2.3
chain F	3.3	3.2	3.1	3.0	1.0	0.0	1.0	1.6
chain G	3.7	3.7	3.6	3.4	1.7	1.0	0.0	1.0
chain H	4.1	4.1	4.0	3.8	2.3	1.6	1.0	0.0

Each pair of chains was superimposed in the region 12-42 for Aβ and region 7-37 for IAPP. The backbone RMSD calculations were performed in the same superimposed region. Rows and columns both correspond to chains A-H of the *closed* heteromeric conformer. The backbone RMSD values reported in the tables are symmetric with diagonal values equal to zero (0.0) as they correspond to the backbone RMSD between a chain and itself.

Table S9C: Backbone RMSD (Å) between all pairs of chains within the *principal open and closed* conformers.

	closed A	*closed* B	*closed* C	*closed* D	*closed* E	*closed* F	*closed* G	*closed* H
open A	5.0	4.8	4.7	4.6	3.7	3.8	4.0	4.4
open B	4.6	4.5	4.4	4.3	3.4	3.5	3.7	4.1
open C	4.4	4.3	4.2	4.1	3.2	3.2	3.4	3.8
open D	4.1	4.0	3.9	3.8	3.1	3.0	3.2	3.6
open E	5.1	5.0	4.9	4.8	3.3	3.0	3.2	3.5
open F	4.8	4.8	4.7	4.6	3.2	2.9	3.0	3.3
open G	4.6	4.6	4.6	4.5	3.1	2.8	2.9	3.2
open H	4.1	4.1	4.0	3.9	2.8	2.7	3.1	3.4

Each pair of chains was superimposed in the region 12-42 for Aβ and region 7-37 for IAPP. The backbone RMSD calculations were performed in the same superimposed region. Rows and columns correspond to chains A-H of the *open* and *closed* heteromeric conformer, respectively.

Table S10: Backbone RMSD (Å) between experimentally resolved polymorphs of Aβ or IAPP and the Aβ and IAPP structures within the *principal open* and *closed* conformers.

Table S10 is attached as an Excel Spreadsheet, due to its size.

PDBs containing multiple conformers are presented intentionally (i.e., 8EZE models 1-6), as they were all considered for the comparison with the IAPP structures. The "*" and "**" denote that the PDBs corresponded to structures with two fibrils, with "*" corresponding to the fibril with the largest number of residues, and "**" corresponding to the fibril with the smallest number of residues. The table is symmetric with both rows and columns corresponding to the Aβ experimentally resolved polymorphs (yellow headings), IAPP experimentally resolved polymorphs (green headings) and the Aβ and IAPP structures of the *open* and *closed* heteromeric models after the conventional MD simulations (purple headings). Each cell of the table contains the backbone RMSD between the corresponding pair of structures (given column and row). The RMSD values reported in the tables are symmetric with the diagonal values equal to zero (0.0) as they correspond to the backbone RMSD between a structure with itself. Each pair of structures in the tables was superimposed in the region 18-42 for Aβ and 13-37 for IAPP, and backbone RMSD calculations were performed in the same superimposed region. Conditional formatting has been applied to the cells, with blue indicating lower RMSD values and red indicating higher RMSD values, to enhance readability. The selected experimentally resolved polymorphs compared with our models were selected based on criteria explained in the supporting methods (S.2.3).

Supporting References

[1] Gremer L, Schölzel D, Schenk C, Reinartz E, Labahn J, Ravelli RBG, Tusche M, Lopez-Iglesias C, Hoyer W, Heise H, Willbold D, Schröder GF. Fibril structure of amyloid-β(1-42) by cryo-electron microscopy. Science. 2017 Oct 6;358(6359):116-119. doi: 10.1126/science.aao2825. Epub 2017 Sep 7. PMID: 28882996; PMCID: PMC6080689.

[2] Lee M, Yau WM, Louis JM, Tycko R. Structures of brain-derived 42-residue amyloid-β fibril polymorphs with unusual molecular conformations and intermolecular interactions. Proc Natl Acad Sci U S A. 2023 Mar 14;120(11):e2218831120. doi: 10.1073/pnas.2218831120. Epub 2023 Mar 9. PMID: 36893281; PMCID: PMC10089215.

[3] Cao Q, Boyer DR, Sawaya MR, Abskharon R, Saelices L, Nguyen BA, Lu J, Murray KA, Kandeel F, Eisenberg DS. Cryo-EM structures of hIAPP fibrils seeded by patient-extracted fibrils reveal new polymorphs and conserved fibril cores. Nat Struct Mol Biol. 2021 Sep;28(9):724-730. doi: 10.1038/s41594-021-00646-x. Epub 2021 Sep 9. PMID: 34518699; PMCID: PMC10396428.

[4] Li D, Zhang X, Wang Y, Zhang H, Song K, Bao K, Zhu P. A new polymorphism of human amylin fibrils with similar protofilaments and a conserved core. iScience. 2022 Dec 1;25(12):105705. doi: 10.1016/j.isci.2022.105705. PMID: 36567711; PMCID: PMC9772857.

[5] Humphrey W, Dalke A, Schulten K. VMD: visual molecular dynamics. J Mol Graph. 1996 Feb;14(1):33-8, 27-8. doi: 10.1016/0263-7855(96)00018-5. PMID: 8744570.

[6] Sievers F, Wilm A, Dineen D, Gibson TJ, Karplus K, Li W, Lopez R, McWilliam H, Remmert M, Söding J, Thompson JD, Higgins DG. Fast, scalable generation of high-quality protein multiple sequence alignments using Clustal Omega. Mol Syst Biol. 2011 Oct 11;7:539. doi: 10.1038/msb.2011.75. PMID: 21988835; PMCID: PMC3261699.

[7] Nanga RP, Brender JR, Vivekanandan S, Ramamoorthy A. Structure and membrane orientation of IAPP in its natively amidated form at physiological pH in a membrane environment. Biochim Biophys Acta. 2011 Oct;1808(10):2337-42. doi: 10.1016/j.bbamem.2011.06.012. Epub 2011 Jun 23. PMID: 21723249; PMCID: PMC3156962.

[8] Jo S, Kim T, Iyer VG, Im W. CHARMM-GUI: a web-based graphical user interface for CHARMM. J Comput Chem. 2008 Aug;29(11):1859-65. doi: 10.1002/jcc.20945. PMID: 18351591.

[9] Brooks BR, Brooks CL 3rd, Mackerell AD Jr, Nilsson L, Petrella RJ, Roux B, Won Y, Archontis G, Bartels C, Boresch S, Caflisch A, Caves L, Cui Q, Dinner AR, Feig M, Fischer S, Gao J, Hodoscek M, Im W, Kuczera K, Lazaridis T, Ma J, Ovchinnikov V, Paci E, Pastor RW, Post CB, Pu JZ, Schaefer M, Tidor B, Venable RM, Woodcock HL, Wu X, Yang W, York DM, Karplus M. CHARMM: the biomolecular simulation program. J Comput Chem. 2009 Jul 30;30(10):1545-614. doi: 10.1002/jcc.21287. PMID: 19444816; PMCID: PMC2810661.

[10] Lee J, Cheng X, Swails JM, Yeom MS, Eastman PK, Lemkul JA, Wei S, Buckner J, Jeong JC, Qi Y, Jo S, Pande VS, Case DA, Brooks CL 3rd, MacKerell AD Jr, Klauda JB, Im W. CHARMM-GUI Input Generator for NAMD, GROMACS, AMBER, OpenMM, and CHARMM/OpenMM Simulations Using the CHARMM36 Additive Force Field. J Chem Theory Comput. 2016 Jan 12;12(1):405-13. doi: 10.1021/acs.jctc.5b00935. Epub 2015 Dec 3. PMID: 26631602; PMCID: PMC4712441.

[11] Jo S, Cheng X, Islam SM, Huang L, Rui H, Zhu A, Lee HS, Qi Y, Han W, Vanommeslaeghe K, MacKerell AD Jr, Roux B, Im W. CHARMM-GUI PDB manipulator for advanced modeling and

simulations of proteins containing nonstandard residues. Adv Protein Chem Struct Biol. 2014;96:235-65. doi: 10.1016/bs.apcsb.2014.06.002. Epub 2014 Aug 24. PMID: 25443960; PMCID: PMC4739825.

[12] Park SJ, Kern N, Brown T, Lee J, Im W. CHARMM-GUI PDB Manipulator: Various PDB Structural Modifications for Biomolecular Modeling and Simulation. J Mol Biol. 2023 Jul 15;435(14):167995. doi: 10.1016/j.jmb.2023.167995. Epub 2023 Feb 2. PMID: 37356910; PMCID: PMC10291205.

[13] Kong L, Park SJ, Im W. CHARMM-GUI PDB Reader and Manipulator: Covalent Ligand Modeling and Simulation. J Mol Biol. 2024 Sep 1;436(17):168554. doi: 10.1016/j.jmb.2024.168554. Epub 2024 Mar 27. PMID: 39237201; PMCID: PMC11377865.

[14] Wang KW, Lee J, Zhang H, Suh D, Im W. CHARMM-GUI Implicit Solvent Modeler for Various Generalized Born Models in Different Simulation Programs. J Phys Chem B. 2022 Sep 29;126(38):7354-7364. doi: 10.1021/acs.jpcb.2c05294. Epub 2022 Sep 18. PMID: 36117287; PMCID: PMC9551160.

[15] Im W, Lee MS, Brooks CL 3rd. Generalized born model with a simple smoothing function. J Comput Chem. 2003 Nov 15;24(14):1691-702. doi: 10.1002/jcc.10321. PMID: 12964188.

[16] Eastman P, Swails J, Chodera JD, McGibbon RT, Zhao Y, Beauchamp KA, Wang LP, Simmonett AC, Harrigan MP, Stern CD, Wiewiora RP, Brooks BR, Pande VS. OpenMM 7: Rapid development of high performance algorithms for molecular dynamics. PLoS Comput Biol. 2017 Jul 26;13(7):e1005659. doi: 10.1371/journal.pcbi.1005659. PMID: 28746339; PMCID: PMC5549999.

[17] Heinig M, Frishman D. STRIDE: a web server for secondary structure assignment from known atomic coordinates of proteins. Nucleic Acids Res. 2004 Jul 1;32(Web Server issue):W500-2. doi: 10.1093/nar/gkh429. PMID: 15215436; PMCID: PMC441567.

[18] Lee MS, Salsbury Jr FR, Brooks III CL. Novel generalized Born methods. The Journal of chemical physics. 2002 Jun 22;116(24):10606-14.

[19] Lee MS, Feig M, Salsbury Jr FR, Brooks III CL. New analytic approximation to the standard molecular volume definition and its application to generalized Born calculations. Journal of computational chemistry. 2003 Aug;24(11):1348-56.

[20] Orr AA, Wördehoff MM, Hoyer W, Tamamis P. Uncovering the Binding and Specificity of β-Wrapins for Amyloid-β and α-Synuclein. J Phys Chem B. 2016 Dec 22;120(50):12781-12794. doi: 10.1021/acs.jpcb.6b08485. Epub 2016 Dec 9. PMID: 27934063.

[21] Orr AA, Shaykhalishahi H, Mirecka EA, Jonnalagadda SVR, Hoyer W, Tamamis P. Elucidating the multi-targeted anti-amyloid activity and enhanced islet amyloid polypeptide binding of β-wrapins. Comput Chem Eng. 2018 Aug 4;116:322-332. doi: 10.1016/j.compchemeng.2018.02.013. Epub 2018 Feb 21. PMID: 30405276; PMCID: PMC6217933.

[22] Orr AA, Gonzalez-Rivera JC, Wilson M, Bhikha PR, Wang D, Contreras LM, Tamamis P. A high-throughput and rapid computational method for screening of RNA post-transcriptional modifications that can be recognized by target proteins. Methods. 2018 Jul 1;143:34-47. doi: 10.1016/j.ymeth.2018.01.015. Epub 2018 Feb 1. PMID: 29408626.

[23] Gonzalez-Rivera JC, Orr AA, Engels SM, Jakubowski JM, Sherman MW, O'Connor KN, Matteson T, Woodcock BC, Contreras LM, Tamamis P. Computational evolution of an RNA-binding protein towards enhanced oxidized-RNA binding. Comput Struct Biotechnol J. 2019 Dec 27;18:137-152. doi: 10.1016/j.csbj.2019.12.003. PMID: 31988703; PMCID: PMC6965710.

[24] Orr AA, Kuhlmann SK, Tamamis P. Computational design of a β-wrapin's N-terminal domain with canonical and non-canonical amino acid modifications mimicking curcumin's proposed inhibitory function. Biophys Chem. 2022 Jul;286:106805. doi: 10.1016/j.bpc.2022.106805. Epub 2022 Mar 26. PMID: 35417810.

[25] Miller LG, Kim W, Schowe S, Taylor K, Han R, Jain V, Park R, Sherman M, Fang J, Ramirez H, Ellington A, Tamamis P, Resendiz MJE, Zhang YJ, Contreras L. Selective 8-oxo-rG stalling occurs in the catalytic core of polynucleotide phosphorylase (PNPase) during degradation. Proc Natl Acad Sci U S A. 2024 Nov 12;121(46):e2317865121. doi: 10.1073/pnas.2317865121. Epub 2024 Nov 4. PMID: 39495922; PMCID: PMC11572968.

[26] Sawaya MR, Hughes MP, Rodriguez JA, Riek R, Eisenberg DS. The expanding amyloid family: Structure, stability, function, and pathogenesis. Cell. 2021 Sep 16;184(19):4857-4873. doi: 10.1016/j.cell.2021.08.013. PMID: 34534463; PMCID: PMC8772536.

[27] UniProt Consortium. UniProt: the Universal Protein Knowledgebase in 2025. Nucleic Acids Res. 2025 Jan 6;53(D1):D609-D617. doi: 10.1093/nar/gkae1010. PMID: 39552041; PMCID: PMC11701636.
